# Technology-Enabled Recreation and Leisure Programs and Activities for Older Adults With Cognitive Impairment: Rapid Scoping Review

**DOI:** 10.2196/53038

**Published:** 2024-08-08

**Authors:** Kristina Marie Kokorelias, Josephine McMurray, Charlene Chu, Arlene Astell, Alisa Grigorovich, Pia Kontos, Jessica Babineau, Jessica Bytautas, Ashley Ahuja, Andrea Iaboni

**Affiliations:** 1 Division of Geriatric Medicine, Department of Medicine Sinai Health System and University Health Network Toronto, ON Canada; 2 Department of Occupational Science & Occupational Therapy Temerty Faculty of Medicine University of Toronto Toronto, ON Canada; 3 National Institute on Ageing Toronto Metropolitan University Toronto, ON Canada; 4 Rehabilitation Sciences Institute Temerty Faculty of Medicine University of Toronto Toronto, ON Canada; 5 Lazaridis School of Business & Economics/Community Health Wilfred Laurier University Waterloo, ON Canada; 6 Lawrence S. Bloomberg Faculty of Nursing University of Toronto Toronto, ON Canada; 7 KITE Research Institute, Toronto Rehabilitation Institute – University Health Network Toronto, ON Canada; 8 Department of Psychiatry Temerty Faculty of Medicine University of Toronto Toronto, ON Canada; 9 Recreation and Leisure Studies Brock University St. Catherine's, ON Canada; 10 Dalla Lana School of Public Health University of Toronto Toronto, ON Canada

**Keywords:** scoping review, review methods, review methodology, knowledge synthesis, synthesis, syntheses, scoping, rapid review, rapid reviews, gerontology, geriatric, geriatrics, older adult, older adults, elder, elderly, older person, older people, ageing, aging, gerontechnology, technology, recreation, recreational, leisure, hobby, hobbies, cognitive, MCI, Alzheimer, dementia, digital health

## Abstract

**Background:**

Recreational and leisure activities significantly contribute to the well-being of older adults, positively impacting physical, cognitive, and mental health. However, limited mobility and cognitive decline often impede access to these activities, particularly for individuals living with dementia. With the increasing availability of digital technologies, there is a rising interest in using technology to deliver recreation and leisure activities for cognitively impaired individuals, acknowledging its potential to provide diverse experiences. The COVID-19 pandemic further highlighted the need for virtual program delivery, especially for individuals in long-term care settings, leading to the development of tools like the Dementia Isolation Toolkit aimed at supporting compassionate isolation. To better support future implementations of the DIT, our rapid scoping review explores evidence-based, technology-enabled recreation programs for older adults with cognitive impairments, which promote well-being.

**Objective:**

We conducted a rapid scoping review of published peer-reviewed literature to answer the following research question: What recreation and leisure programs or activities are being delivered using technology to adults living with dementia or another form of cognitive impairment?

**Methods:**

In total, 6 databases were searched by an Information Specialist. Single reviewers performed title or abstract review, full-text screening, data extraction, and study characteristic summarization.

**Results:**

A total of 92 documents representing 94 studies were identified. The review identified a variety of technology-enabled delivery methods, including robots, gaming consoles, tablets, televisions, and computers, used to engage participants in recreational and leisure activities. These technologies impacted mood, cognition, functional activity, and overall well-being among older adults with cognitive impairments. Activities for socializing were the most common, leveraging technologies such as social robots and virtual companions, while relaxation methods used virtual reality and digital reminiscence therapy. However, challenges included technological complexity and potential distress during reminiscing activities, prompting recommendations for diversified research settings, and increased sample sizes to comprehensively understand technology's impact on leisure among this demographic.

**Conclusions:**

The findings suggest that technology-enabled recreational activities, such as socializing, relaxation and self-awareness activities, music and dance, exergaming, and art, can positively impact the mood and overall well-being of older adults with cognitive impairment. Future research should embrace a more inclusive approach, integrating design, diverse settings, and a broader sample of older adults to develop technology-driven leisure activities tailored to their unique needs and promote their effective use.

## Introduction

### Background

Participating in recreational and leisure activities is a significant contributor to the health and well-being of older adults [1,2]. Recreation and leisure activities include pursuits such as dancing, walking, singing, or playing a musical instrument, creative pastimes such as painting, pottery or woodworking, and a wide variety of sports and games. Recreation and leisure activities can be enjoyed alone or as part of a group and have been shown to positively benefit older adults’ physical and cognitive function and mental health [3].

In later life, a range of factors can reduce opportunities and access to recreation and leisure pursuits including limited mobility [4] and cognitive loss [5]. People living with dementia, for example, face multiple barriers to continued participation in recreation and leisure activities [6-8] due to progressive cognitive decline. Consequently, limited access to recreation and leisure activities negatively impacts people living with dementia or other forms of cognitive impairment through lack of socialization and stimulation [9,10].

### Technology-Enabled Delivery of Recreation and Leisure Activities

The use of technology to deliver recreation and leisure activities for people living with impaired cognition, is becoming more commonplace [11-15], with the recognition that technology can facilitate in-person leisure as well as new forms of uniquely digital experiences [16,17]. The rise in interest may reflect the increasing availability of digital technologies, from tablets to robots. Touchscreens, for instance, are particularly accessible for people living with dementia as they provide immediate feedback through touch [13]. As such, touchscreen tablets and larger devices have been successfully used to deliver a variety of recreation activities including games [13], reminiscing activities [18,19], and music [20]. Touchscreens have also been tested in the form of telepresence robots—simple, nonhumanoid frames with a touchscreen that can be controlled to move on flat surfaces [21]. Art is another popular target for technology for people living with dementia including virtual reality [22] and virtual reality tours of art galleries [23], making and viewing art together on tablets [24,25] and art therapy [11,26]. More energetic activities using motion-based game systems such as the Wii [27] and Xbox [28] have been shown to not only promote physical activity but also socialization and enjoyment [29].

### COVID-19 Heightened the Need for Web-Based Program Delivery

The impact of a lack of recreation and leisure activities for people living with cognitive disabilities was underscored during the COVID-19 pandemic. Compromised cognitive functioning, language, insight, and judgment associated with dementia impact the ability of individuals to understand and appreciate the necessity of isolation and to voluntarily comply with isolation procedures [30]. The enforcement of isolation protocols to prevent the transmission of the virus during the pandemic drastically reduced recreation and leisure activities for people living with dementia [31]. Long-term care (LTC) home staff faced significant challenges in enforcing these protocols, leading to ethical dilemmas and moral distress as they navigated the balance between ensuring safety and promoting the well-being of residents [31-33]. At this stage of the pandemic, outbreaks of infectious diseases, including COVID-19, remain frequent events in LTC homes, and there is an ongoing need for the delivery of recreation opportunities for residents in isolation.

The Dementia Isolation Toolkit (DIT) was developed to support compassionate, safe, and effective isolation of people living with dementia in LTC settings and contains a series of tools designed to provide ethical, legal, and clinical guidance to support decision-making (34,35). It also includes methods and approaches, including those that are technology enabled, to support safe isolation for individuals living with cognitive impairment, ensuring their dignity and well-being. Given the wide variety of technologies and digital activities being developed and tested for older adults living with impaired cognition [12,15,36], we conducted a rapid scoping review to identify technology-enabled recreation and leisure programs or activities that are being delivered to older adults living with cognitive impairment. Our aim was to identify programs with supporting evidence of efficacy, which might complement the DIT and facilitate its adoption and use in LTC. While reviews exist focusing on technological interventions for individuals living with dementia, they often focus on loneliness rather than other benefits of recreation [15].

## Methods

### Research Design

We conducted a rapid scoping review of published peer-reviewed literature. A rapid review was selected to allow us to generate timely results to inform the design of novel digital interventions to deliver recreation and leisure activities for people living with cognitive impairment [37]. However, we combined rapid review methodology with scoping review methodology to allow us to map the key issues or topics in a research area where the literature has not been reviewed comprehensively, and many different study designs may be applicable [38,39]. This review was conducted following Arksey and O’Malley’s [40] scoping review methodology and was informed by the Cochrane guidance on rapid reviews [41]. The review is reported following the PRISMA-ScR (Preferred Reporting Items for Systematic Reviews and Meta-Analyses extension for Scoping Reviews) framework [42] (see Multimedia Appendix 1).

### Step 1: Identifying the Research Question

Based on the knowledge and experience of the multidisciplinary DIT team and familiarity with the literature on technology for recreation and leisure for persons living with dementia, we identified the following research question: What recreation and leisure programs or activities are being delivered using technology to adults living with dementia or another form of cognitive impairment?

### Step 2: Identifying Relevant Studies

On January 20, 2021, the following health and technology databases were searched using a search strategy developed by the research team, which included an Information Specialist (JB):ACM Digital Library, CENTRAL (Ovid), CINAHL (EBSCO), Embase (Ovid), IEEE Explore, and MEDLINE (Ovid). No limitation was set on publication year. When possible, searches were limited to include only English-language publications and primary research articles.

The searches for CINAHL (EBSCO) and MEDLINE (Ovid) were then updated on April 11, 2024 (see Multimedia Appendix 2), using the same search strategy. The decision for selecting these 2 databases was done by analyzing included studies from the original search to determine from which databases the studies were retrieved. All the studies selected for inclusion were retrieved from these 2 databases, and thus, to expedite the process of the update, an informed decision was made to focus updates on these 2 databases.

In addition to comprehensive database searching, the reference lists of included studies were reviewed for relevant studies.

### Stage 3: Study Selection

The study selection process consisted of 2 stages: first by screening titles and abstracts; and second, by full-text screening. To be eligible for inclusion at both stages, the article must have reported on primary research that included an evaluation of a technology and explored the experiences of older adults with cognitive impairment using the technology. The technology must have been used to deliver or enable recreation and leisure programs. Inclusion and exclusion criteria are presented in Table 1.

Inclusion criteria were refined iteratively throughout each stage of the screening process (title and abstract, full text), as recommended by Levac et al [43]. First, all team members screened the same subset of titles and abstracts to calibrate the inclusion criteria. Then, one team member screened approximately 25% of the titles and abstracts [41]. All team members screened another subset of titles and abstracts to further refine the inclusion criteria [41]. Finally, the remaining approximately 75% of the titles and abstracts were divided amongst 5 team members and individually screened.

All team members reviewed an initial subset of full texts to calibrate our inclusion criteria. Twenty percent of the remaining full-text articles were double reviewed, and discrepancies were resolved through discussion.

**Table 1:** Inclusion and exclusion criteria.

Criteria	Inclusion	Exclusion
Language of the studies	English	Languages other than English
Study design	Empirical research articles (eg, qualitative, randomized controlled trials [RCTs], quasi-experimental designs, observational studies [eg, cohort studies, case-control studies), cross-sectional studies, longitudinal studies, pre-post studies, mixed methods studies)– reporting on an evaluation focused on older adults. Must explore experiences of older adults.	Nonempirical study designs, such as reviews. Exclude conference abstracts. The evaluation should not solely be on the technology or the caregivers.
Intervention	Recreation and leisure (eg, arts-based interventions, music, dance, games, exergaming, recreational activities, recreation, leisure activities, creative, games, exergaming, cognitive stimulation therapy, socializing, and social interactions) “program” or “activity” for adults aged 18 years or older with cognitive impairment.	The intervention should not focus on the assessment, monitoring or detection of cognitive impairment (if no game component or reference to experience).
Mode of delivery	Delivered using technology (eg, app, device, platforms, robot). Technology must have leisure component.	Technology that is used to monitor or detect cognitive impairment.
Population	Adults aged 50 years or older with cognitive impairment including (but not limited to) dementia, Wernicke encephalopathy, delirium, amnestic, Alzheimer disease, organic brain disease or syndrome, benign senescent forgetfulness, Binswanger, Korsakoff syndrome, stroke-related cognitive impairment, Wilhelmsen-Lynch disease, aphasia, Benson syndrome, Huntington's disease, mild cognitive impairment or disorder, Creutzfeldt Jacob disease, or Parkinson disease	The populations cannot be at risk for cognitive impairment prevention (ie, older adults who are not currently cognitively impaired). Internet gaming disorder and addiction or alcoholism-related disorders are excluded.

### Stage 4: Charting the Data

Our data were charted and sorted according to areas of potential relevance to the research questions including (1) country in which the study was conducted; (2) study site; (3) type of activity; (4) sample size; (5) population age range; (6) sex, if available; (7) type of cognitive impairment; (8) research question or aims; (9) study methods; (10) description of technology; (11) outcomes or findings; and (12) feasibility, as described by study authors. Double data extraction was conducted on the final set of articles included in this review. Using Microsoft Excel, 2 research assistant team members charted the data. A third team member reviewed the charting and coded the data extraction into categories, where relevant.

We did not assess the quality of included studies, as quality assessments are neither required nor appropriate for scoping review methodology [39,43].

### Stage 5: Summarizing and Reporting the Data

Data were organized numerically using descriptive statistics and summarized using a narrative descriptive synthesis by members of the research team that included gerontologists, nurses, psychologists, and health researchers who provided their perspectives on the findings [44]. The constructs considered for review included age, patient population, technology used, and outcomes.

## Results

### Overview

**Figure 1:**
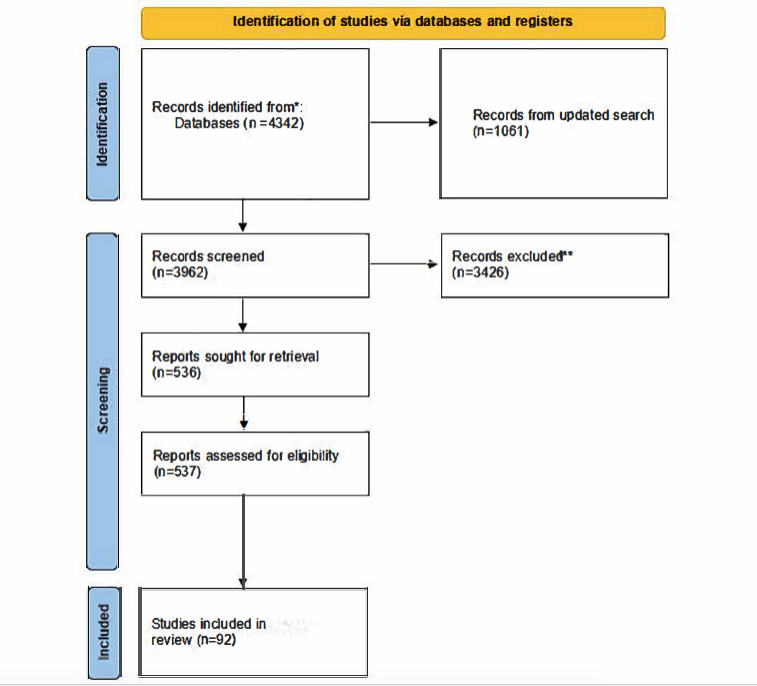
PRISMA (Preferred Reporting Items for Systematic Reviews and Meta-Analyses) flow diagram.

Our initial search yielded 4342 results, with a further 1061 results following a search update. Following deduplication, 3962 results were eligible for screening. The screening process resulted in a total of 92 documents, 61 from the original searches, and an additional 31 documents in the updated search. See Figure 1 for the PRISMA (Preferred Reporting Items for Systematic Reviews and Meta-Analyses) flow diagram [42].

Publication dates ranged from 2000 to 2022, with most (57/92, 62%) published between 2016 and 2021, which confirmed an expected interest in the topic over time. Of these 92 documents, 1 paper [45] reported on 3 studies, resulting in a total of 88 studies for analysis. The studies were conducted in multiple countries; mostly in the United States, Canada, and the United Kingdom. Table S2 in Multimedia Appendix 3 outlines the key characteristics of the included studies.

From these studies, 46 employed mixed methods (46/94, 49%), 28 were qualitative (28/94, 30%) and 21 were quantitative (21/94, 22%). Among the qualitative studies, the most common methods for data collection were interviews (9/28, 32%) [45-53] and observational techniques (15/28, 54%) [28,45,47,48,51,54-63]. Among the quantitative studies, the most common methods of data collection consisted of experimental data collection (16/21, 76%) [64-79] and surveys or questionnaires (4/21, 19%) [74,80-82].

### Participants Targeted

The sample sizes ranged from 1 [54,81,82] to 139 [84] participants. In total, this review contained 2332 participants. Ages ranged from 50 [85] to 104 [86] years. There were inconsistencies with reporting patient demographics with 11 studies (11/94, 12%) failing to report age range or mean [48,51,56,64,65,71,87-91]. Most studies indicated that they recruited both male and female participants (65/94, 69%). The primary clinical indicator for participants was dementia (unspecified) (60/94, 64%), followed by Alzheimer disease (9/94, 9.5%), and mild cognitive impairment (12/94, 13%). An additional 8 studies (10/94, 11%) reported a mixed form of dementia. Most participants were recruited from either residential care (eg, assisted living) facilities (40/94, 42.5%), day care (eg, senior) centers (19/94, 20%), home (13/94, 14%), hospital inpatients (4/94, 4%) or other health care settings (2/94, 2%), and hospice (1/94, 1%). Only a few studies (14/94, 15%) reported on the ethnicity of participants [24,52,54,57,63,69,83,86,92-97], with mostly White participants participating in all but 1 study [92]. Table S3 in Multimedia Appendix 4 outlines the key characteristics of participants.

### Types of Leisure Activities

The types of technology-enabled recreational and leisure activities for older adults with cognitive impairment were categorized as follows: (1) socializing; (2) relaxation and self-awareness; (3) music and dance; (4) exergaming; (5) video or audio (nonmusic) entertainment; (6) playing games; and (7) art. Socializing (46/94, 49%) [48,50,53,58,63,65, 66,70-72,76,80,82,84,86,91-94,96,98-114] was the most commonly used recreational and leisure activity; 20 (20/94, 21%) studies used a combination of activities. Examples of how socializing activities were fostered by technology included the use of social (companion [111]) robots (eg, PARO [82,86,102,106,109,110] and MARIO [99,103,113]), online pet companions [101], social recognition watch [93], and Skype on Wheels [87]. According to some articles, these technologies facilitated social engagement as they recognized the gestures, emotions, stimuli, and speech of older adults and engaged them in active conversation [66,98,104,108].

The prevalence of socialization activities was followed by relaxation and self-awareness activities (22/94, 22%) studies [50,52,63-65,68,75,76,81,88,92,94,98,100,102,111,115-121]. These activities were facilitated through various means including virtual reality [65,115,116], computer activities [94], digital life storybooks [121], digital reminiscence therapy [75,111]). Table S4 in Multimedia Appendix 5 outlines the category of activities used in each study.

### Types of Technological Delivery

There was a wide range of technology-enabled delivery methods. Most technologies were commercially available. Table S5 in Multimedia Appendix 6 outlines the origin of technology used in each study indicating whether it was obtained commercially (57/94, 61%), developed in-house (17/94, 18%), or a combination of both (20/94, 23%). However, it is important to note that the studies included in the analysis did not provide sufficient information regarding the technological development process.

In addition to robots, as previously mentioned, some studies used game consoles, including those that accurately track the participants' arm, hand, and body movement as well as facial expressions [62,74,84,122,123]. Some interventions were rooted in artificial intelligence to address social and emotional needs by engaging with older adults with speech and touch [46,65,80,98,99,113,124]. Some interventions used tablets [24,49-51,58,60,75,83,85,100,101,105,114,116,119,124-128], televisions [47,49,62,64,68,73,88,120,121], or computers [61,87,90,92,94,95,98,115,116,129,130] to engage participants in auditory and visual activities by stimulating cognition such as through memory stimulation. Other studies used technologies that facilitate simulated and interactive experiences with near-eye displays or touchscreen displays to boost active experience among older adults [85,100,119]. Some of the other tablets included iPads or television sets for software-driven visual interfaces or stimulation, such as creating self-portraits or life stories for older adults [52,58-60,81,87,92,93,96,109,121,131]. The tablets were used to connect residents with friends and family [72,105]. Some researchers delivered musical interventions using devices familiar to older adults, including the radio or MP3 players [45,57,66,87,99,120,131-134].

### Outcomes of Interest

The majority of the articles reported positive outcomes (64/92, 69.5%), while a smaller portion had mixed results (28/92, 30%). Table S6 in Multimedia Appendix 7 outlines the measurement of the outcomes, and results. Positive outcome studies generally relied on descriptions of participant experience, such as analysis of interview conversations [50,59,91,135,136], questionnaires [53,78,81,100] and observations [48,49,54,66,68,72,85,97,102,115]. Studies with mixed outcomes tended to rely on the use of measurement tools like physiological tests [87] and observation [47,51,82,94,96,103,124,126,129]. In this context, positive/neutral/negative outcomes refer to the effectiveness and acceptability of the intervention among participants, reflecting both favorable and unfavorable responses.

### Mood and Overall Well-Being

In total, 50 articles explored mood and overall well-being (50/94, 53%) [24,28,46-49,51,55,57,61,64,66-68,70,71,74,76,80-82, 84,86,87,89,95,96,98,100-102,106-112,114-116,118,119,123,124,126,129,132,137,138]. Platforms such as computers and tablets [87,96,100,119] that helped to deliver virtual reality [115,116,118], exergames [28,74,123], and robotic companions [66,70,71,84,98] overall led to an improvement in mood and overall well-being, including feelings of gratitude [128] and behavioral symptoms of dementia [67]. Mood improvement was primarily measured via techniques such as surveys and questionnaires [46,47,81,98,100,108,112,123] including physiological assessment questionnaires [87] such as the UCLA loneliness scale or Geriatric Depression scale [80]. Improvements in mood were caused by either one or a combination of the following technology-enabled activities: socializing [49,91], music and dance [45,48,49,55,59,61,64,67,76,85,114,129,132], video or audio (nonmusic) entertainment [47,57,101], and art [24,81,124]. Where companionship was a targeted outcome, engagement with technology-enabled activities boosted feelings of excitement and belonging while also decreased feelings of depression, anxiety, and loneliness [80,107,108]. For example, robots or online pets facilitated companionship [101,108]. Online companions were largely pets that provided comfort to older adults that increased mood by allowing them to cuddle, play, or pet them [82,86,106,107,109,110], or watching, touching, or caring for the robot [71,101,138]. One study, however, found an increase in anxiety in individuals with mild cognitive impairment when using robotic pets as companions [101], which was contrary to an intervention which used music [76]. Games and activities with satisfying achievements for completion encouraged high self-esteem and validation among participants, especially when the challenges matched their cognitive abilities [28,61,95,119,125,126] or allowed autonomous art creation or viewing [24,51,124]. Another study which used a mobile-reminiscing therapy app found no change in mood [111].

### Cognitive Health

A total of 19 articles (19/94, 20%) explored improvements to cognition facilitated through the use of exergames, tablet and computer applications, and robots or music, which provided stimulation [45-47,52,54-57,59,69,75,81,85,114,121,123,131, 132,139]. Technologies that engaged participants in physical activity led to an outcome of improved cognitive health (although this was not defined) as such leisure activities stimulated motor skills often used in athletics [46,54,56]. Exergames were found to increase activity, only if the individual had sufficient cognitive ability (eg, having mild dementia vs severe) [123]. This was measured by a combination of usability testing processes and semistructured interviews [46], or observation combined with field notes ([54,54]. Opportunities to facilitate memories, often facilitated through videos, photos, and music encouraged expressive community engagement and relationship-building through shared experiences [45,47,52,55,57,59,75,85,132,139], provided beneficial cognitive stimuli that helped with conversation, which in turn was believed to be an indicator of improved cognitive health [81,121,131].

### Functional Activity

Five articles (5/94, 5%) explored improvements to functioning in daily life. These were facilitated through exercising (via exergames [73,78]), robotic stimulation, and general time management and behavioral strategies [57,73,98,112]. One study classified improved functioning according to the World Health Organization’s International Classification of Functioning, Disability and Health [70]. One study found that improved functioning included being able to maintain a schedule [98] and reduce fidgeting [57]. The studies found negative, or no improvement to sleep [57] and memory [93]. There are contrary findings around physical activity through exergames, with 2 studies suggesting negative or no improvement [73,131], and 1 study found that virtual reality cycling improved physical activity [122].

## Discussion

The use of technology to deliver recreation and leisure activities for people living with impaired cognition is becoming more commonplace [11-15]. This rapid scoping review identifies and describes the existing literature that describes technologies used in recreation and leisure programs or activities that are delivered to older adults living with cognitive impairment. Our review found a diverse range of activities for older adults with cognitive impairment aged 50 [85] to 104 [86] years old, related to (1) socializing, (2) relaxation and self-awareness, (3) music and dance, (4) exergaming, (5) video or audio (nonmusic) entertainment, (6) playing games, and (7) art. Numerous technologies supported these activities and programs including the use of tablets [24,49-51,58,60,72,75,83,85,100,101,105, 114,116,119,124-128], televisions [47,49,52,58-60,62,64,68,73, 81,87,88,92,93,96,111,120,121,131], radio and MP3 players [45,57,66,87,99,120,131,132,134] or computers [6,7,61,87,90-92,94,95,98,115,116,129,130]. Touchscreen displays were frequently used to engage older adults in their activities [85,100,119], and some incorporated the use of artificial intelligence [48,49,80]. The technologies focused on obtaining various outcomes, including improving mood [24,28,46-49,51,55,57,61,64,66-68,70,71,74,76,80-82,84, 86,87,89,95,96,98,100-102,106-112,114-116,118,119,123,124,126,129,132,137,138] and cognitive stimulation [45-47,52,54-57,59,69,75, 81,85,114,121,123,131,132,139]. Many of the included studies reported positive results, supporting the use and effectiveness of some technologies to support recreation and leisure activities. However, study results should be interpreted within the context of their small sample size [56,71,84,92,99-101,107,111,128,131,134] and the lack of consideration for older adults with diverse cognitive and physical disabilities [71,74,84,100].

### Technology-Related Challenges Within the Context of Recreation and Leisure Activities for Older Adults Living With Cognitive Impairment

Across the articles, authors raised numerous concerns about the use of technology to facilitate recreation and leisure activities for older adults with cognitive impairment. For instance, authors cautioned that technologies that focus on social interactions are not replacements for human companionship [98,100]. In the context of activities focused on reminiscing, some studies found that older adults may experience distress while observing photographs of deceased family members [99]. Technologies that use multimodal interactions (ie, verbal and visual) may be challenging and confusing for some people living with advanced stages of dementia [49,113]. Likewise, older adults’ interest in, and acceptance of Wii and exergames games varied based on their cognitive health; people living with severe dementia were more likely to reject the games; whereas people living with mild dementia enjoyed exergaming but still needed supervision [123]. However, exergaming may not be cost-effective compared to usual treatment [78].

With reminiscence therapy, the process of obtaining relevant artifacts was time-consuming, and required commitment from family members [83]. The use of gaming systems for older adults also raised technical and ethical concerns for some scholars [92,125], as they may perpetuate low self-esteem, insecurity, and annoyance due to a lack of familiarity with the technology and lack of digital literacy [125,126]. Lastly, radios may experience reception issues (eg, static, signal dropping out) that can bother the participants [134]. Beyond the challenges with the technology itself, some studies reported that the technology was expensive, which may present a barrier to wide-spread implementation [78,82,84,109,138].

Many interventions discussed in this review, relied on costly robots, such as PARO [82,86,102,106,109,110], MARIO [99,103,113], and pet companions [101]. While pet companions increase older adults’ mood, and robots allow them to care for these robotic pets which in turn increases their enjoyment, robot pets can also cause anxiety in individuals with mild cognitive impairment [101]. These results suggest the need for future research using similar interventions and may provide ideas for testable hypotheses to further investigate the benefits of using robots, including the target population and optimal timing during the illness trajectory. In comparison to tablets, robots can be prohibitively expensive for many older adults and care settings [140-142]. However, robots can often be customized to older adults’ preferences [143,144] unlike other off-the-shelf technologies, and thus might provide broader assistance in their daily lives and overall quality of life. Exploring options to adapt or customize lower-cost technologies like tablets to older adults’ preferences may support wider adoption. Moreover, future studies are encouraged to provide more detailed information on how customizations occurred, and the development process for novel technologies. Engaging older adults with cognitive impairments and other stakeholders, such as care partners and health care providers, through a co-design design approach could add value when developing new technologies to support appropriate leisure and recreational activities. This approach helps researchers and technology developers gain in-depth insights into the preferences of different targeted populations [145-147].

### Facilitators to Using Technology Within the Context of Recreation and Leisure Activities for Older Adults Living With Cognitive Impairment

Although there were some challenges with the technologies, many studies identified facilitators to their use across a variety of settings, such as having technical support easily accessible to older adults who were not familiar with technology [50]. In addition, hosting the technology in friendly spaces (eg, supportive environment, praise, and freedom to ask questions) helped older adults feel welcome to learn about new technologies [100]. A study found that the technical skills for gaming activities such as Nintendo Wii were learned, retained, and transferred to other leisure activities [54]. The availability of both technical support and emotional support is critical for older people who may not be as comfortable with the technologies as younger people [100]. Moreover, it is important for trainers to know how to communicate with and teach new skills to people living with dementia [28]. For example, certain types of prompts such as verbal prompts might not work well with some older adults [28]. Therefore, trainers must be capable and have a broad range of knowledge translation experience and problem-solving abilities so that people living with dementia will be optimally positioned to learn these new skills [28]. The use of animation and video might also make training processes more effective [81].

One study used robots that included infrared cameras that sent alerts to caregivers and nursing stations in case of emergency, and reminders for scheduled activities [112]. Robots with humanlike characteristics, including variable expressions, helped to engage older adults in recreational activities [108].

Researchers also found that older adults with mild cognitive impairment engage more in computer-based applications if they are provided in a gamified environment [50]. Computer systems with wheels were convenient for residents living in LTC homes as they could be transported from one room to another [92]. One system included a computer, webcam, microphone, speakers, hand or foot pedal for exercise and therapy, joystick, headset, and adjustable height unit for residents to allow play when standing or sitting [92]. Other computer systems could be set up using the existing television in resident rooms [121]. When feasible, setting up a new telephone line specifically for technology can help overcome connectivity and reception issues [134]. It is important to note that when computer activities match the interests and cognitive abilities of residents living with dementia in LTC homes, there is an increase in participation and satisfaction [61]. Verbal encouragement from LTC staff can also facilitate the use of technology [45]. Additionally, technology can help support staff deliver reminiscence therapy without additional training [111].

Few studies in our review reported information about participant ethnicity and comorbid conditions. Studies have shown that the digital divide (ie, the gap between those who have access, knowledge and use of technology, and those who do not [148]) is most pronounced for some racial and ethnic groups [149-151] and older adults [152]. Relying on technology to facilitate connection and belonging, and socialization and enjoyment between residents or with their families, may, therefore, exacerbate existing disparities in the well-being of older adults [153]. To address these disparities and promote inclusivity, research should explore the experiences of diverse older adults when using technology to support social and recreational leisure activities. Other barriers to be overcome include cognition [154], physical ability [154], low research literacy [155], lack of cultural competency [155], and speech- and language barriers [154,155]. These barriers occur in dementia research more broadly and have led to the underrepresentation of certain groups in research [155], limiting the generalizability of existing research.

To overcome these barriers, collaboration with community partners can be instrumental in ensuring inclusive recruitment and data collection strategies [154,156]. Such efforts can increase the representativeness of research samples and improve the translation of research findings to diverse populations and settings, into more effective and equitable technology interventions for engaging older adults in social and recreational leisure activities.

### Methodological Recommendations for Researchers

The following 3 recommendations for scientists conducting research in this domain emerged from our analysis of the included articles:

1. Conduct research across settings: Most studies focused on a single setting, but it is suggested that research should be conducted in multiple settings such as home, community care, and health care institutions, because outcomes may vary due to the specific characteristics of each setting [45,99,115]. In addition, including individuals at various stages of cognitive impairment is crucial, as outcomes may vary between early-stage and advanced cognitive impairment [47,115,116]. In fact, few studies recommend prioritizing people with more advanced stages (moderate to severe) of cognitive impairment since there are severe challenges in managing symptoms and improving quality of life [106,115,116].
2. Increase the sample size and representation: Authors emphasized the need to increase sample size, which would allow greater demographic diversity in the research of older adults using technology for leisure and recreation [56,71,84,92,99-101,107,111,128,131,134]. This would include people with various types of disabilities [71,74,84,100], and a greater number of male participants to gain a better understanding of gender in technology adoption [75]. Furthermore, some scholars have argued for an increase in caregiver samples to help explore how technologies could support them in their caregiving duties and help alleviate their stress, which is often overlooked within existing research [59,100,118].
3. Increase the use of experimental study design: Many studies recommended the use of different research methods, particularly experimental designs that use a control group to understand potential confounding factors [46,82,86,92,99,101,104,129,132]. This will help address questions about the validity of clinical outcomes [101,132]. Additionally, obtaining time series data on adoption and efficacy of technology will also help obtain deeper insights [46,56,57,96,108,118].

This review demonstrates that many existing technologies can support the socialization, relaxation, self-awareness and meaningful recreation and leisure activities of older adults, including playing games and creating art. Existing research has highlighted that engaging older adults living with dementia in meaningful activities can improve their quality of life [157-159]. An existing review explored the use of technology to promote engagement in adults with dementia living in residential aged care [159], whereas another explored technological interventions such as robots, tablets, and computers in the context of loneliness among individuals with dementia [15]. Our review expands on this existing knowledge by incorporating the diverse settings in which older adults engage in social activities, including hospices and community settings. However, a previous review noted that the benefits of engagement are not caused by the technologies themselves but rather in the opportunities the technologies provided to facilitate connection and belonging [159]. Therefore, more research is needed to understand the impact and benefits of technologies to facilitate connection and belonging, in comparison to standard care. Thus, a critical lesson from this review is the need to explore the existing barriers to connection and belonging, as well as the unique functions that technology can provide compared to those that can be provided by individuals such as formal and informal care partners.

In summary, our review confirms the growing interest among researchers in integrating technology into recreational and leisure activities for older adults, with most articles being published in the last 7 years. However, while there is interest in using technologies, there is a lack of large-scale, experimental studies, over time. Several factors may contribute to the limited experimental research in this area including the upfront costs of technology for older adults [160], older adults’ training needs [161], and concerns regarding the long-term sustainability of these technology-enabled programs [162,163]. Implementation research is crucial to the scalability of technologies that might support adoption and sustainability [164,165]; its scarcity is notable in the existing body of literature. Additionally, the literature rarely described technologies being used across multiple care settings or the progression of diseases or conditions. Future research with different stages and settings will provide more insight into the diverse perspectives and values that participants bring when considering leisure activities [51].

### Limitations

This study had several limitations. First, we only included English-language literature and excluded gray literature and conference abstracts, which may present preliminary findings. Consequently, it is possible that relevant literature was not captured by our search. Lastly, while we ran comprehensive electronic searches and adhered to an established methodology [40], the nature of a rapid scoping review including only 1-screener may have resulted in missed articles.

### Conclusions

Technology has continued to emerge as a way to help engage older adults living with dementia in social and recreational leisure activities. Despite the availability of various digital technologies and their evaluation studies in the context of older adults, the literature is very sparse regarding how and how well they are developed, adopted, sustained, and evaluated. Current studies focus on the use of tablets, robots, televisions, computers, exergames, and radios, but little is known about the acceptability and feasibility of them in diverse settings, or about their clinical effectiveness. Moreover, included articles lack discussion on the adaptation of these technologies for older adults living with cognitive impairment and various forms of disabilities. Future research should take a more inclusive approach, incorporating design and development (ie, co-design approaches), testing, and implementation of technologies in diverse settings including home, community care, and health care institutions, and include a more diverse sample of older adults. By considering the specific needs and challenges faced by older adults living with cognitive impairment and other types of disabilities, researchers can develop technology-enabled recreation and leisure activities that are better suited to their unique requirements and promote their effective use in different contexts.

## Appendix

Multimedia Appendix 1 PRISMA checklist.

Multimedia Appendix 2 Search strategy.

Multimedia Appendix 3 Characteristics of included peer-reviewed studies.

Multimedia Appendix 4 Target and actual sample.

Multimedia Appendix 5 Types of activities.

Multimedia Appendix 6 Creation of technology.

Multimedia Appendix 7 Study outcomes and measurements.

## Abbreviations

DIT: Dementia Isolation Toolkit
LTC: long-term care
PRISMA: Preferred Reporting Items for Systematic Reviews and Meta-Analyses
PRISMA-ScR: Preferred Reporting Items for Systematic Reviews and Meta-Analyses extension for Scoping Reviews

## Data Availability

The data sets used in this study are available upon request. Access to these data sets can be requested by contacting KK at kristina.kokorelias@sinaihealth.ca. While not publicly accessible, all reasonable requests for data will be considered in compliance with our institutional policies and regulatory obligations.
