# Supplementary material for: Technology-Enabled Recreation and Leisure Programs and Activities for Older Adults With Cognitive Impairment: Rapid Scoping Review
Source: JMIR Neurotechnol. 2024 Aug 8;3:e53038. doi: 10.2196/53038 (PMC12671325; doi:10.2196/53038)
Supplement: Multimedia Appendix 2 [file neuro_v3i1e53038_app2.doc]

**Database: Ovid MEDLINE(R) ALL <1946 to April 11, 2024>**
**Search Strategy:**
**1**  exp Dementia/ (213702)
**2**  Wernicke Encephalopathy/ (1930)
**3**  Delirium, Dementia, Amnestic, Cognitive Disorders/ (9848)
**4**  dement*.tw,kf,jw. (160708)
**5**  alzheimer*.tw,kf,jw. (204871)
**6**  (lewy* adj2 bod*).tw,kf. (12483)
**7**  (chronic adj2 cerebrovascular).tw,kf. (842)
**8**  ('organic brain disease' or 'organic brain syndrome').tw,kf. (818)
**9**  ('normal pressure hydrocephalus' and 'shunt*').tw,kf. (1667)
**10**  'benign senescent forgetfulness'.tw,kf. (18)
**11**  (cerebr* adj2 deteriorat*).tw,kf. (266)
**12**  (cerebral* adj2 insufficient*).tw,kf. (96)
**13**  (pick* adj2 disease).tw,kf. (3776)
**14**  binswanger*.tw,kf. (606)
**15**  korsako*.tw,kf. (1780)
**16**  (frontotemporal adj3 degeneration?).tw,kf. (3692)
**17**  FTD.tw,kf. (5459)
**18**  FTDP.tw,kf. (493)
**19**  DDPAC.tw,kf. (7)
**20**  FTLD.tw,kf. (2398)
**21**  FLDEM.tw,kf. (0)
**22**  wilhelmsen lynch disease?.tw,kf. (1)
**23**  HDDD?.tw,kf. (19)
**24**  exp Aphasia, Primary Progressive/ (1223)
**25**  primary progressive aphasia?.tw,kf. (1898)
**26**  mesulam* syndrome?.tw,kf. (3)
**27**  (non-fluent adj3 aphasia?).tw,kf. (470)
**28**  (nonfluent adj3 aphasia?).tw,kf. (541)
**29**  (Logopenic adj3 aphasia?).tw,kf. (214)
**30**  posterior cortical atrophy.tw,kf. (605)
**31**  benson* syndrome?.tw,kf. (8)
**32**  huntington*.tw,kf. (21757)
**33**  CADASIL.tw,kf. (1606)
**34**  CADASILm.tw,kf. (1)
**35**  "Cerebral autosomal dominant arteriopathy with subcortical infarcts and leukoencephalopathy".tw,kf. (1055)
**36**  "Cerebral arteriopathy with subcortical infarcts and leukoencephalopathy".tw,kf. (7)
**37**  Familial vascular leukoencephalopathy.tw,kf. (1)
**38**  creutzfeldt ja#ob.tw,kf. (7098)
**39**  ja#ob creutzfeldt.tw,kf. (113)
**40**  CJD.tw,kf. (3289)
**41**  subacute spongiform encephalopath*.tw,kf. (162)
**42**  or/1-41 [Dementia and Subterms] (368916)
**43**  mild cognitive impairment?.tw,kf. (26683)
**44**  mild cognitive disorder?.tw,kf. (140)
**45**  MCI.tw,kf. (24754)
**46**  mild neurocognitive impairment?.tw,kf. (43)
**47**  mild neurocognitive disorder?.tw,kf. (277)
**48**  ((cognit* or neurocognit* or memory or cerebr* or mental*) adj3 (declin* or deteriorat* or degenerat*)).tw,kf. (55080)
**49**  exp cognition disorder/ (120484)
**50**  ((cognit* or neurocognit*) adj3 (impairment? or disorder?)).tw,kf. (122524)
**51**  or/43-50 [MCI and select Cognitive Decline Terms] (228323)
**52**  42 or 51 [ALL CI Terms] (504689)
**53**  exp Technology/ (508123)
**54**  exp Telemedicine/ (47135)
**55**  Artificial Intelligence/ (45148)
**56**  exp Software/ (187563)
**57**  computer simulation/ or augmented reality/ or virtual reality/ (220951)
**58**  exp Computers/ (87787)
**59**  exp Technology Assessment, Biomedical/ (12298)
**60**  technolog*.tw,kf,jw. (909856)
**61**  ((remote or remotely) adj3 (stream* or deliver*)).tw,kf. (2232)
**62**  artificial* intelligen*.tw,kf. (49850)
**63**  (telehealth* or tele-health* or telemed* or tele-med* or mhealth or m-health).tw,kf,jw. (52834)
**64**  ((virtual or augment*) adj2 realit*).tw,kf. (23357)
**65**  (computer* or smartphone? or phone? or cellphone? or tablet? or handheld or hand-held or iphone? or ipad? or android? or touchscreen? or touch-screen?).tw,kf. (503318)
**66**  (mobile adj3 (application? or app? or device? or technolog* or computer*)).tw,kf. (25604)
**67**  (portable adj3 (application? or app? or device? or technolog* or computer?)).tw,kf. (8094)
**68**  or/53-67 [Technology Terms] (2159926)
**69**  exp Sensory Art Therapies/ (56002)
**70**  exp Leisure Activities/ (283311)
**71**  Recreation Therapy/ (145)
**72**  (recreation? or recreational).tw,kf. (34357)
**73**  (leisure or leisurely).tw,kf. (20861)
**74**  (arts or artistic*).tw,kf. (14192)
**75**  (art adj2 (intervention? or therap* or activit* or interact* or perform* or visual* or enrich* or creat* or install* or program*)).tw,kf. (25034)
**76**  art?-based.tw,kf. (1254)
**77**  (music* or dance? or dancing).tw,kf. (36262)
**78**  (create or creative* or creativity).tw,kf. (215380)
**79**  (game? or gaming or exergame? or exergaming).tw,kf. (55081)
**80**  "cognitive stimulation therap*".tw,kf. (176)
**81**  (socializ* or socialis*).tw,kf. (17708)
**82**  (social adj2 (interact* or connect* or relationship?)).tw,kf. (52474)
**83**  or/69-82 [Leisure and Art Terms] (745240)
**84**  52 and 68 and 83 (2037)
**85**  84 not ((adolescent/ or exp child/ or exp infant/) not exp adults/) (1943)
**86**  limit 85 to english language (1885)
**87**  ("20221019" or 2022102* or 2022103* or 202211* or 202212* or 2023* or 2024*).dt,ez,da. (2553249)
**88**  86 and 87 (311)

Launch search (Available to TeamUHN only): <https://uhn.idm.oclc.org/login?url=http://ovidsp.ovid.com/ovidweb.cgi?T=JS&NEWS=N&PAGE=main&SHAREDSEARCHID=5sGbX4NunNxbiWrEtvKD7wyA8fEbPoVaYSTg9V6UZaABiemvxka7lOzAa1TLVdfRq>

Database: CINAHL Ultimate (EBSCO)
Date searched: 2024-04-12

Top of Form

| **#** | **Query** | **Limiters/Expanders** | **Last Run Via** | **Results** |
| --- | --- | --- | --- | --- |
| S1 | (MH "Dementia+") | Expanders - Apply equivalent subjects Search modes - Boolean/Phrase | Interface - EBSCOhost Research Databases Search Screen - Advanced Search Database - CINAHL Ultimate | 86,094 |
| S2 | (MH "Wernicke's Encephalopathy") | Expanders - Apply equivalent subjects Search modes - Boolean/Phrase | Interface - EBSCOhost Research Databases Search Screen - Advanced Search Database - CINAHL Ultimate | 498 |
| S3 | (MH "Delirium, Dementia, Amnestic, Cognitive Disorders") | Expanders - Apply equivalent subjects Search modes - Boolean/Phrase | Interface - EBSCOhost Research Databases Search Screen - Advanced Search Database - CINAHL Ultimate | 144 |
| S4 | TI dement* OR AB dement* OR SO dement* | Expanders - Apply equivalent subjects Search modes - Boolean/Phrase | Interface - EBSCOhost Research Databases Search Screen - Advanced Search Database - CINAHL Ultimate | 73,399 |
| S5 | TI alzheimer* OR AB alzheimer* OR SO alzheimer* | Expanders - Apply equivalent subjects Search modes - Boolean/Phrase | Interface - EBSCOhost Research Databases Search Screen - Advanced Search Database - CINAHL Ultimate | 50,742 |
| S6 | TI lewy* n2 bod* OR AB lewy* n2 bod* | Expanders - Apply equivalent subjects Search modes - Boolean/Phrase | Interface - EBSCOhost Research Databases Search Screen - Advanced Search Database - CINAHL Ultimate | 2,841 |
| S7 | TI chronic n2 cerebrovascular OR AB chronic n2 cerebrovascular | Expanders - Apply equivalent subjects Search modes - Boolean/Phrase | Interface - EBSCOhost Research Databases Search Screen - Advanced Search Database - CINAHL Ultimate | 209 |
| S8 | TI ( 'organic brain disease' or 'organic brain syndrome' ) OR AB ( 'organic brain disease' or 'organic brain syndrome' ) | Expanders - Apply equivalent subjects Search modes - Boolean/Phrase | Interface - EBSCOhost Research Databases Search Screen - Advanced Search Database - CINAHL Ultimate | 136 |
| S9 | TI ( 'normal pressure hydrocephalus' and 'shunt*' ) OR AB ( 'normal pressure hydrocephalus' and 'shunt*' ) | Expanders - Apply equivalent subjects Search modes - Boolean/Phrase | Interface - EBSCOhost Research Databases Search Screen - Advanced Search Database - CINAHL Ultimate | 207 |
| S10 | TI 'benign senescent forgetfulness' OR AB 'benign senescent forgetfulness' | Expanders - Apply equivalent subjects Search modes - Boolean/Phrase | Interface - EBSCOhost Research Databases Search Screen - Advanced Search Database - CINAHL Ultimate | 3 |
| S11 | TI (cerebr* n2 deteriorat*) OR AB (cerebr* n2 deteriorat*) | Expanders - Apply equivalent subjects Search modes - Boolean/Phrase | Interface - EBSCOhost Research Databases Search Screen - Advanced Search Database - CINAHL Ultimate | 76 |
| S12 | TI (cerebral* n2 insufficient*) OR AB (cerebral* n2 insufficient*) | Expanders - Apply equivalent subjects Search modes - Boolean/Phrase | Interface - EBSCOhost Research Databases Search Screen - Advanced Search Database - CINAHL Ultimate | 22 |
| S13 | TI pick* n2 disease OR AB pick* n2 disease | Expanders - Apply equivalent subjects Search modes - Boolean/Phrase | Interface - EBSCOhost Research Databases Search Screen - Advanced Search Database - CINAHL Ultimate | 415 |
| S14 | TI binswanger* OR AB binswanger* | Expanders - Apply equivalent subjects Search modes - Boolean/Phrase | Interface - EBSCOhost Research Databases Search Screen - Advanced Search Database - CINAHL Ultimate | 58 |
| S15 | TI korsako* OR AB korsako* | Expanders - Apply equivalent subjects Search modes - Boolean/Phrase | Interface - EBSCOhost Research Databases Search Screen - Advanced Search Database - CINAHL Ultimate | 268 |
| S16 | TI subacute spongiform encephalopath* OR AB subacute spongiform encephalopath* | Expanders - Apply equivalent subjects Search modes - Boolean/Phrase | Interface - EBSCOhost Research Databases Search Screen - Advanced Search Database - CINAHL Ultimate | 2 |
| S17 | TI CJD OR AB CJD | Expanders - Apply equivalent subjects Search modes - Boolean/Phrase | Interface - EBSCOhost Research Databases Search Screen - Advanced Search Database - CINAHL Ultimate | 525 |
| S18 | TI ja?ob creutzfeldt OR AB ja?ob creutzfeldt | Expanders - Apply equivalent subjects Search modes - Boolean/Phrase | Interface - EBSCOhost Research Databases Search Screen - Advanced Search Database - CINAHL Ultimate | 5 |
| S19 | TI creutzfeldt ja?ob OR AB creutzfeldt ja?ob | Expanders - Apply equivalent subjects Search modes - Boolean/Phrase | Interface - EBSCOhost Research Databases Search Screen - Advanced Search Database - CINAHL Ultimate | 1,200 |
| S20 | TI ( "Cerebral arteriopathy with subcortical infarcts and leukoencephalopathy" ) OR AB ( "Cerebral arteriopathy with subcortical infarcts and leukoencephalopathy" ) | Expanders - Apply equivalent subjects Search modes - Boolean/Phrase | Interface - EBSCOhost Research Databases Search Screen - Advanced Search Database - CINAHL Ultimate | 1 |
| S21 | TI ( CADASIL or CADASILm ) OR AB ( CADASIL or CADASILm ) | Expanders - Apply equivalent subjects Search modes - Boolean/Phrase | Interface - EBSCOhost Research Databases Search Screen - Advanced Search Database - CINAHL Ultimate | 386 |
| S22 | TI ( "Cerebral autosomal dominant arteriopathy with subcortical infarcts and leukoencephalopathy" ) OR AB ( "Cerebral autosomal dominant arteriopathy with subcortical infarcts and leukoencephalopathy" ) | Expanders - Apply equivalent subjects Search modes - Boolean/Phrase | Interface - EBSCOhost Research Databases Search Screen - Advanced Search Database - CINAHL Ultimate | 285 |
| S23 | TI huntington* OR AB huntington* | Expanders - Apply equivalent subjects Search modes - Boolean/Phrase | Interface - EBSCOhost Research Databases Search Screen - Advanced Search Database - CINAHL Ultimate | 2,638 |
| S24 | TI benson* syndrome* OR AB benson* syndrome* | Expanders - Apply equivalent subjects Search modes - Boolean/Phrase | Interface - EBSCOhost Research Databases Search Screen - Advanced Search Database - CINAHL Ultimate | 2 |
| S25 | TI posterior cortical atrophy OR AB posterior cortical atrophy | Expanders - Apply equivalent subjects Search modes - Boolean/Phrase | Interface - EBSCOhost Research Databases Search Screen - Advanced Search Database - CINAHL Ultimate | 223 |
| S26 | TI (nonfluent n3 aphasia*) OR AB (nonfluent n3 aphasia*) | Expanders - Apply equivalent subjects Search modes - Boolean/Phrase | Interface - EBSCOhost Research Databases Search Screen - Advanced Search Database - CINAHL Ultimate | 267 |
| S27 | TI mesulam* syndrome* OR AB mesulam* syndrome* | Expanders - Apply equivalent subjects Search modes - SmartText Searching | Interface - EBSCOhost Research Databases Search Screen - Advanced Search Database - CINAHL Ultimate | 4 |
| S28 | TI primary progressive aphasia* OR AB primary progressive aphasia* | Expanders - Apply equivalent subjects Search modes - Boolean/Phrase | Interface - EBSCOhost Research Databases Search Screen - Advanced Search Database - CINAHL Ultimate | 776 |
| S29 | TI HDDD# OR AB HDDD# | Expanders - Apply equivalent subjects Search modes - Boolean/Phrase | Interface - EBSCOhost Research Databases Search Screen - Advanced Search Database - CINAHL Ultimate | 2 |
| S30 | TI wilhelmsen lynch disease* OR AB wilhelmsen lynch disease* | Expanders - Apply equivalent subjects Search modes - Boolean/Phrase | Interface - EBSCOhost Research Databases Search Screen - Advanced Search Database - CINAHL Ultimate | 0 |
| S31 | TI wilhelmsen lynch disease* OR AB wilhelmsen lynch disease* | Expanders - Apply equivalent subjects Search modes - SmartText Searching | Interface - EBSCOhost Research Databases Search Screen - Advanced Search Database - CINAHL Ultimate | 1 |
| S32 | TI ( FTD or FTDP or DDPAC or FTLD or FLDEM ) OR AB ( FTD or FTDP or DDPAC or FTLD or FLDEM ) | Expanders - Apply equivalent subjects Search modes - Boolean/Phrase | Interface - EBSCOhost Research Databases Search Screen - Advanced Search Database - CINAHL Ultimate | 1,450 |
| S33 | TI (frontotemporal n3 degeneration*) OR AB (frontotemporal n3 degeneration*) | Expanders - Apply equivalent subjects Search modes - Boolean/Phrase | Interface - EBSCOhost Research Databases Search Screen - Advanced Search Database - CINAHL Ultimate | 777 |
| S34 | TI mild neurocognitive impairment? OR AB mild neurocognitive impairment? | Expanders - Apply equivalent subjects Search modes - Boolean/Phrase | Interface - EBSCOhost Research Databases Search Screen - Advanced Search Database - CINAHL Ultimate | 6 |
| S35 | TI mild neurocognitive disorder* OR AB mild neurocognitive disorder* | Expanders - Apply equivalent subjects Search modes - Boolean/Phrase | Interface - EBSCOhost Research Databases Search Screen - Advanced Search Database - CINAHL Ultimate | 86 |
| S36 | TI mild cognitive disorder* OR AB mild cognitive disorder* | Expanders - Apply equivalent subjects Search modes - Boolean/Phrase | Interface - EBSCOhost Research Databases Search Screen - Advanced Search Database - CINAHL Ultimate | 24 |
| S37 | TI MCI OR AB MCI | Expanders - Apply equivalent subjects Search modes - Boolean/Phrase | Interface - EBSCOhost Research Databases Search Screen - Advanced Search Database - CINAHL Ultimate | 6,991 |
| S38 | TI mild cognitive impairment* OR AB mild cognitive impairment* | Expanders - Apply equivalent subjects Search modes - Boolean/Phrase | Interface - EBSCOhost Research Databases Search Screen - Advanced Search Database - CINAHL Ultimate | 10,147 |
| S39 | TI ( ((cognit* or neurocognit*) n3 (impairment? or disorder?)) ) OR AB ( ((cognit* or neurocognit*) n3 (impairment? or disorder?)) ) | Expanders - Apply equivalent subjects Search modes - Boolean/Phrase | Interface - EBSCOhost Research Databases Search Screen - Advanced Search Database - CINAHL Ultimate | 40,429 |
| S40 | TI ( ((cognit* or neurocognit* or memory or cerebr* or mental*) n3 (declin* or deteriorat* or degenerat*)) ) OR AB ( ((cognit* or neurocognit* or memory or cerebr* or mental*) n3 (declin* or deteriorat* or degenerat*)) ) | Expanders - Apply equivalent subjects Search modes - Boolean/Phrase | Interface - EBSCOhost Research Databases Search Screen - Advanced Search Database - CINAHL Ultimate | 18,225 |
| S41 | (MH "Mild Cognitive Impairment") OR (MH "Cognition Disorders") | Expanders - Apply equivalent subjects Search modes - Boolean/Phrase | Interface - EBSCOhost Research Databases Search Screen - Advanced Search Database - CINAHL Ultimate | 37,074 |
| S42 | S1 OR S2 OR S3 OR S4 OR S5 OR S6 OR S7 OR S8 OR S9 OR S10 OR S11 OR S12 OR S13 OR S14 OR S15 OR S16 OR S17 OR S18 OR S19 OR S20 OR S21 OR S22 OR S23 OR S24 OR S25 OR S26 OR S27 OR S28 OR S29 OR S30 OR S31 OR S32 OR S33 OR S34 OR S35 OR S36 OR S37 OR S38 OR S39 OR S40 OR S41 | Expanders - Apply equivalent subjects Search modes - Boolean/Phrase | Interface - EBSCOhost Research Databases Search Screen - Advanced Search Database - CINAHL Ultimate | 172,542 |
| S43 | (MH "Technology+") | Expanders - Apply equivalent subjects Search modes - Boolean/Phrase | Interface - EBSCOhost Research Databases Search Screen - Advanced Search Database - CINAHL Ultimate | 91,752 |
| S44 | (MH "Telemedicine+") OR (MH "Communications Media") OR (MH "Telecommunications+") | Expanders - Apply equivalent subjects Search modes - Boolean/Phrase | Interface - EBSCOhost Research Databases Search Screen - Advanced Search Database - CINAHL Ultimate | 181,088 |
| S45 | (MH "Artificial Intelligence") OR (MH "Computer Simulation") OR (MH "Virtual Reality") OR (MH "Augmented Reality") | Expanders - Apply equivalent subjects Search modes - Boolean/Phrase | Interface - EBSCOhost Research Databases Search Screen - Advanced Search Database - CINAHL Ultimate | 39,675 |
| S46 | (MH "Software+") | Expanders - Apply equivalent subjects Search modes - Boolean/Phrase | Interface - EBSCOhost Research Databases Search Screen - Advanced Search Database - CINAHL Ultimate | 487,428 |
| S47 | (MH "Computers and Computerization+") | Expanders - Apply equivalent subjects Search modes - Boolean/Phrase | Interface - EBSCOhost Research Databases Search Screen - Advanced Search Database - CINAHL Ultimate | 877,663 |
| S48 | TI technolog* OR AB technolog* | Expanders - Apply equivalent subjects Search modes - Boolean/Phrase | Interface - EBSCOhost Research Databases Search Screen - Advanced Search Database - CINAHL Ultimate | 147,290 |
| S49 | TI ( ((remote or remotely) n3 (stream* or deliver*)) ) OR AB ( ((remote or remotely) n3 (stream* or deliver*)) ) | Expanders - Apply equivalent subjects Search modes - Boolean/Phrase | Interface - EBSCOhost Research Databases Search Screen - Advanced Search Database - CINAHL Ultimate | 994 |
| S50 | TI artificial* intelligen* OR AB artificial* intelligen* | Expanders - Apply equivalent subjects Search modes - Boolean/Phrase | Interface - EBSCOhost Research Databases Search Screen - Advanced Search Database - CINAHL Ultimate | 9,318 |
| S51 | TI ( (telehealth* or tele-health* or telemed* or tele-med* or mhealth or m-health) ) OR AB ( (telehealth* or tele-health* or telemed* or tele-med* or mhealth or m-health) ) | Expanders - Apply equivalent subjects Search modes - Boolean/Phrase | Interface - EBSCOhost Research Databases Search Screen - Advanced Search Database - CINAHL Ultimate | 19,374 |
| S52 | TI ( ((virtual or augment*) n2 realit*) ) OR AB ( ((virtual or augment*) n2 realit*) ) | Expanders - Apply equivalent subjects Search modes - Boolean/Phrase | Interface - EBSCOhost Research Databases Search Screen - Advanced Search Database - CINAHL Ultimate | 7,445 |
| S53 | TI ( (computer* or smartphone# or phone# or cellphone# or tablet# or handheld or hand-held or iphone# or ipad# or android# or touchscreen# or touch-screen#) ) OR AB ( (computer* or smartphone# or phone# or cellphone# or tablet# or handheld or hand-held or iphone# or ipad# or android# or touchscreen# or touch-screen#) ) | Expanders - Apply equivalent subjects Search modes - Boolean/Phrase | Interface - EBSCOhost Research Databases Search Screen - Advanced Search Database - CINAHL Ultimate | 111,475 |
| S54 | TI ( (mobile n3 (application# or app# or device# or technolog* or computer*)) ) OR AB ( (mobile n3 (application# or app# or device# or technolog* or computer*)) ) | Expanders - Apply equivalent subjects Search modes - Boolean/Phrase | Interface - EBSCOhost Research Databases Search Screen - Advanced Search Database - CINAHL Ultimate | 10,108 |
| S55 | TI ( (portable n3 (application# or app# or device# or technolog* or computer#)) ) OR AB ( (portable n3 (application# or app# or device# or technolog* or computer#)) ) | Expanders - Apply equivalent subjects Search modes - Boolean/Phrase | Interface - EBSCOhost Research Databases Search Screen - Advanced Search Database - CINAHL Ultimate | 1,506 |
| S56 | S43 OR S44 OR S45 OR S46 OR S47 OR S48 OR S49 OR S50 OR S51 OR S52 OR S53 OR S54 OR S55 | Expanders - Apply equivalent subjects Search modes - Boolean/Phrase | Interface - EBSCOhost Research Databases Search Screen - Advanced Search Database - CINAHL Ultimate | 1,115,898 |
| S57 | (MH "Art Therapy") OR (MH "Dance Therapy") OR (MH "Music Therapy") | Expanders - Apply equivalent subjects Search modes - Boolean/Phrase | Interface - EBSCOhost Research Databases Search Screen - Advanced Search Database - CINAHL Ultimate | 11,824 |
| S58 | (MH "Leisure Activities+") | Expanders - Apply equivalent subjects Search modes - Boolean/Phrase | Interface - EBSCOhost Research Databases Search Screen - Advanced Search Database - CINAHL Ultimate | 81,291 |
| S59 | (MH "Recreational Therapy") | Expanders - Apply equivalent subjects Search modes - Boolean/Phrase | Interface - EBSCOhost Research Databases Search Screen - Advanced Search Database - CINAHL Ultimate | 1,816 |
| S60 | TI ( recreation# or recreational ) OR AB ( recreation# or recreational ) | Expanders - Apply equivalent subjects Search modes - Boolean/Phrase | Interface - EBSCOhost Research Databases Search Screen - Advanced Search Database - CINAHL Ultimate | 13,876 |
| S61 | TI ( leisure or leisurely ) OR AB ( leisure or leisurely ) | Expanders - Apply equivalent subjects Search modes - Boolean/Phrase | Interface - EBSCOhost Research Databases Search Screen - Advanced Search Database - CINAHL Ultimate | 10,906 |
| S62 | TI ( arts or artistic* ) OR AB ( arts or artistic* ) | Expanders - Apply equivalent subjects Search modes - Boolean/Phrase | Interface - EBSCOhost Research Databases Search Screen - Advanced Search Database - CINAHL Ultimate | 40,766 |
| S63 | TI ( (art n2 (intervention? or therap* or activit* or interact* or perform* or visual* or enrich* or creat* or install* or program*)) ) OR AB ( (art n2 (intervention? or therap* or activit* or interact* or perform* or visual* or enrich* or creat* or install* or program*)) ) | Expanders - Apply equivalent subjects Search modes - Boolean/Phrase | Interface - EBSCOhost Research Databases Search Screen - Advanced Search Database - CINAHL Ultimate | 11,174 |
| S64 | TI art#-based OR AB art#-based | Expanders - Apply equivalent subjects Search modes - Boolean/Phrase | Interface - EBSCOhost Research Databases Search Screen - Advanced Search Database - CINAHL Ultimate | 940 |
| S65 | TI ( music* or dance# or dancing OR create or creative* or creativity ) OR AB ( music* or dance# or dancing OR create or creative* or creativity ) | Expanders - Apply equivalent subjects Search modes - Boolean/Phrase | Interface - EBSCOhost Research Databases Search Screen - Advanced Search Database - CINAHL Ultimate | 91,394 |
| S66 | TI ( game# or gaming or exergame# or exergaming ) OR AB ( game# or gaming or exergame# or exergaming ) | Expanders - Apply equivalent subjects Search modes - Boolean/Phrase | Interface - EBSCOhost Research Databases Search Screen - Advanced Search Database - CINAHL Ultimate | 22,870 |
| S67 | TI "cognitive stimulation therap*" OR AB "cognitive stimulation therap*" | Expanders - Apply equivalent subjects Search modes - Boolean/Phrase | Interface - EBSCOhost Research Databases Search Screen - Advanced Search Database - CINAHL Ultimate | 158 |
| S68 | TI ( socializ* or socialis* ) OR AB ( socializ* or socialis* ) | Expanders - Apply equivalent subjects Search modes - Boolean/Phrase | Interface - EBSCOhost Research Databases Search Screen - Advanced Search Database - CINAHL Ultimate | 8,617 |
| S69 | TI ( (social n2 (interact* or connect* or relationship#)) ) OR AB ( (social n2 (interact* or connect* or relationship#)) ) | Expanders - Apply equivalent subjects Search modes - Boolean/Phrase | Interface - EBSCOhost Research Databases Search Screen - Advanced Search Database - CINAHL Ultimate | 24,256 |
| S70 | S57 OR S58 OR S59 OR S60 OR S61 OR S62 OR S63 OR S64 OR S65 OR S66 OR S67 OR S68 OR S69 | Expanders - Apply equivalent subjects Search modes - Boolean/Phrase | Interface - EBSCOhost Research Databases Search Screen - Advanced Search Database - CINAHL Ultimate | 266,379 |
| S71 | S42 AND S56 AND S70 | Expanders - Apply equivalent subjects Search modes - Boolean/Phrase | Interface - EBSCOhost Research Databases Search Screen - Advanced Search Database - CINAHL Ultimate | 1,895 |
| S72 | S42 AND S56 AND S70 | Expanders - Apply equivalent subjects Search modes - Boolean/Phrase | Interface - EBSCOhost Research Databases Search Screen - Advanced Search Database - CINAHL Ultimate | 1,895 |
| S73 | ZD(2022102* or 2022103* or 202211* or 202212* or 2023* or 2024*) | Limiters - English Language Expanders - Apply equivalent subjects Search modes - Boolean/Phrase | Interface - EBSCOhost Research Databases Search Screen - Advanced Search Database - CINAHL Ultimate | 358,725 |
| S74 | S72 AND S73 | Expanders - Apply equivalent subjects Search modes - Boolean/Phrase | Interface - EBSCOhost Research Databases Search Screen - Advanced Search Database - CINAHL Ultimate | 253 |

Bottom of Form
