# Supplementary material for: Technology-Enabled Recreation and Leisure Programs and Activities for Older Adults With Cognitive Impairment: Rapid Scoping Review
Source: JMIR Neurotechnol. 2024 Aug 8;3:e53038. doi: 10.2196/53038 (PMC12671325; doi:10.2196/53038)
Supplement: Multimedia Appendix 3 [file neuro_v3i1e53038_app3.docx]

**Table 2— Characteristics of Included Peer-Reviewed Studies**

| **First Author, Year, Country & Design** | **Objective** | **Setting** | **Description of Technology** | **Key Results and Conclusions** |
| --- | --- | --- | --- | --- |
| Abdollahi et al., 2017 [44]  USA  Qualitative | To evaluate the likelihood of long-term companionship, likeability and acceptance, and preferred features of the Ryan Robot. | Residential care facility & institution | Ryan Companionbot is based on the Expressionbot. Developed in DreamFace Technologies, LLC. This robot has an emotive and expressive face with accurate visual speech. Ryan can maintain a spoken dialog, recognize expressions on the user’s face, and it is equipped with a screen on its torso with features such as cognitive games, music player, narrated photo album, and video player. Ryan was customized for each participant. Surveys also conducted. | The subjects also spent approximately two hours and ten minutes per day interacting with Ryan in different tasks such as playing cognitive games, having conversations, viewing family photo albums, listening to music, etc. They have spent a great amount of time with the robot and their interests in speaking with the robot did not decay over time. The survey revealed that the subjects liked interacting with Ryan and accepted the robot as a companion although it cannot replace human companionship. They also believed the robot helped them maintain their schedule, improved their mood, and stimulated them mentally. |
| Álvarez, 2022 [45]  USA  Prospective, single-anonymized, crossover-  group design | Aimed to understand whether Neurologic Music Therapy (NMT)  techniques can be implemented with a habilitative approach  for older adults with moderate to severe dementia. | Assisted living community | NMT is an impairment-focused behavioral intervention system based on the clinical neuroscience of music perception, cognition, and production | It had a positive effect on NMT on general cognitive functioning for residents with moderately severe cognitive decline was remarkable. These results suggest that NMT with a habilitative approach is a viable tool to engage older adults with dementia. |
| Appel et al., 2020 [46]  Canada  Mixed Methods | To evaluate the feasibility of using immersive VR (virtual reality) with a sample of older adults within hospital in- and out-patient settings and long-term care residences, and to determine whether these experiences can be beneficial | Day care center & long-term care facility | Head mounted Samsung GearVR HMD immersive VR Technology with 360-degree video of nature.  The VR hardware system consisted of the following components: (1) Samsung S7 smartphone to view the VR films, (2) Samsung VR HMD that housed the smartphone/viewing screen and restricted the view of the real world, (3) Sennheiser HD 221 headphones to present the sound of the films and minimize the sound of the surrounding environment, and (4) VRology sanitary replaceable face-pads for individual use. | All participants completed the study with no negative side-effects reported (e.g., No dizziness, disorientation, interference with hearing aids); the average time spent in VR was 8min and 76% of participants viewed the entire experience at least once. Participants tolerated the HMD very well; most had positive feedback, feeling more relaxed and adventurous; 76% wanted to try VR again. Better image quality and increased narrative video content were suggested to improve the experience |
| Appel et al., 2021 [47]  Canada  Mixed Methods | Participants used a Samsung Gear VR HMD and Sennheiser HD 221 headphones. | Inpatient hospital care | Participants used a Samsung Gear VR HMD and Sennheiser HD 221 headphones. | The main finding of this pilot study was that the VR intervention was well accepted by the patient participants, consisting of individuals with a multitude of sensory, cognitive, and physical health conditions, including advanced dementia, limited mobility, and use of hearing and vision aids. In addition, despite many observed BPSD symptoms during their hospitalizations, none of the patients displayed aggressive or agitated symptoms during VR therapy, and none were actively averse to its application. |
| Assche et al., 2021  [48]  Belgium  Qualitative | To investigate the experiences of older adults with mild cognitive impairment in the use of socially-assistive robots (SARs) during the first COVID-19 pandemic lockdown. | Independent living facilities | An existing SAR called James developed by Zora Robotics LLC in Belgium was used. It was known that these SARs would perform both assistive functions and socialized with the user. No other details or descriptions of the technology were provided. | The thematic analysis revealed four themes in the post-intervention interviews with the participants: companionship, meaningful activities, technology and technical aspects, and barriers or areas of improvement. Overall, participants found the robot to be quite a nice companion that provided them company; specifically, the presence alleviated feelings of isolation and loneliness and brought comfort during difficult times. In addition to this, the presence of the robot aided in cognitive exercises, physical activity, entertainment, and more. Moreover, it seemed to motivate the participants to engage in new and meaningful activities more frequently. While the technical aspects of the robot are certainly less impressive than computers and smartphones, their interactiveness and simple interface were welcomed. Furthermore, the human-like nature of the robot was also very welcoming and comforting. The most mentioned challenges and barriers include finding a place for it after the lockdowns, being unable to communicate meaningfully for an extended period of time, and potential cost and price concerns. Overall, the authors concluded that SARs are a beneficial and promising avenue of “treatment” for social isolation and loneliness and further research is warranted. |
| Astell et al., 2016 [49]  UK  Mixed Methods | To investigate the effect of familiarity on usability and enjoyment by people with dementia, focusing on apps identified as games (as opposed to sensory experiences). | Residential care facility & day care center. | Apple iPad (fourth generation) running iOS 7 was used in all data collection. Operating system settings were set to provide an optimal experience for the user and to prevent unnecessary or accidental interference during gameplay. Brightness and volume were both set to maximum; app notifications were all turned off; and multitasking gestures were turned off. The free version of the app Solitaire (by MobilityWare) was the game presented to participants in Group 1. The premium version of the app Bubble Xplode (by Spooky House Studios) was presented to participants in Group 2. | Outcome measures included independently initiating gameplay, independently advancing through a game, gameplay length & enjoyment.  Ninety percent of participants attempted gameplay independently with 17% of participants in the familiar group reaching the checkpoint compared with 93% playing the novel game. Regardless of which game was played or whether the checkpoint was reached, 88% of all participants reported enjoyment of the gaming sessions. |
| Barrett et al., 2019 [50]  Ireland  Mixed Methods | To evaluate the acceptability, functionality, and usability of MARIO for people living with dementia (PWD) in a nursing home, as well as any potential ethical issues, from the perspective of the PWD interacting with MARIO and the researcher observing the interactions; and (b) to explore the short-term effect of MARIO on quality of life, depression, and perceived social support in PWD. | Nursing home | Mario uses the Kompaı2 robot platform developed by Robosoft. It is a robot equipped with a camera, a Kinect, and two LiDAR sensors for indoor navigation, object detection, and obstacle avoidance. A tablet PC is located on the robot torso for inter-action that is loaded with apps, including a music app, a reminiscence app, news app, etc. | All chosen applications were generally enjoyed by the PWD. While using MyMusic, PWD were observed to be singing, dancing, smiling, and telling stories about their youth. In MyGames, the most commonly selected games were painting and coloring. However, two of 10 PWD did not like playing games and were not interested in this application. Using MyMemories, the majority of PWD were observed to enjoy looking at old photographs, particularly those containing family members. However, two PWD experienced negative emotions at times while observing photographs containing family members who had since passed away. The researcher provided support to PWD at these times. PWD frequently initiated interactions with staff members to tell them about or show them MARIO and the applications. For example, they sometimes chose to show their old photographs, displayed in MyMemories, to other people around them. |
| Benham et al., 2022 [51]  Country not specified  Exploratory study | To examine the effects of immersive virtual reality (VR)  on self-identified daily activities and quality of life (QOL) of older adults with and without cognitive impairments and identify their preferred VR apps. | Not specified | VR has allowed participation in  activities that pose greater physical safety risks in the real world. | In a community-based setting, leisure-oriented  immersive VR may improve daily activity perceptions for older  adults, regardless of mild cognitive impairments. |
| Berge et al., 2022 [52]  Country not specified  Mixed methods | This study (1) describes the development of a prototype  of a music application for patients in a gerontopsychiatric  hospital following an expert-driven participatory design process  and (2) evaluates the acceptability, adoption, and feasibility  of use for dyads of home-dwelling persons with dementia  and their informal caregivers in a clinical trial in municipal  dementia care. | Home | The application “Alight” was developed following an iterative,  expert-driven participatory design approach, which includes a requirement  elicitation phase and two rounds of prototyping and testing in real-world  settings. | The feasibility and adoption of the application were high and  accepting dyads did not differ on demographic and clinical variables from those not reached. This suggests a high potential for utilization in dementia care. This study contributes methodologically to the field of participatory  design and mHealth interventions by demonstrating a specific design approach that throughout the process successfully involved researchers, industry partners, health care practitioners, and end users. |
| Chen et al., 2021 [53]  China  Mixed Methods | To understand the acceptance of tablets among cognitively impaired older adults when used in cognitive training. | Residential care facility | Tablets offered on loan by the service providers in the current study. | Tablets were perceived as beneficial on cognition, enjoyment, learning, social relationships, and communication. When implementing tablet-based interventions, stakeholders should note that technology is used for encouraging and facilitating social interactions and is not meant to replace or reduce human contact. The current study found that tablet-based interventions provided by volunteers could encourage social relationships and increase well-being, and hence potentially facilitate aging-in-place. |
| Cheung et al., 2023 [54]  China  Clustered randomized controlled trial | To evaluate the clinical efficacy of the intervention and to examine the  effectiveness of the implementation strategies in promoting home-based adoption. | Home | Not specified | There was a significant reduction in the anxiety and the symptoms of depression of those in the intervention group. There was also a significant reduction in the perceived stress level of the caregivers. Music-with-movement interventions showed promise for improving the well-being of people with dementia and their caregivers. Effective strategies facilitated the implementation process, such as integrating communication technology to provide instant support and involving volunteers in engaging the collaborating centres and families. |
| Chidester et al., 2016 [55]  USA  Mixed Methods | Not specified - described as a study on the effects of computer technology use on increasing socialization and improving mental health in nursing home and memory support patients. | Long-term care facility | Touch-screen computer. The content was standardized with 10 core applications as well as additional content customized based on participant’s individual interests, such as sports, videos, music or brain games to name just a few. Inside the customized home screen, iN2L created a custom button that has the community’s audio files of the Chaplain sermons, town hall meeting videos, weekly menus, activity calendars and campus news to keep the participants engaged in the local happenings around the community. | 40% of patients, when engaged with the computer had a clinically significant reduction of total doses of antipsychotic medications then when they did not have the computer. Behavioral episodes became less frequent for 54% of participants, ceasing entirely for 30% of those. They became less intense for 75% of participants. Depression decreased 40%. Cognitive / brain power scores rose for nearly 23.5%, with an average increase of 4.86 points on a 30-point scale. Stress indicators for staff caregivers dropped by 47%. The Affect Balance Scale, which indicates mood, increased significantly for all study participants (test and group) over the entire 24 weeks. |
| Chu et al., 2017 [56]  Australia  Mixed Methods | To show how the engagement between two social robots and PwD in Australian residential care facilities can improve care quality. | Residential care facility | Sophie and Jack are designed specifically for emotional and intentional communication and interaction purposes. They have been designed in collaboration with Nippon Electric Company (NEC), Japan. Both robots with baby-face deliver diversion therapy services that involve functions such as face recognition, subject registration and tracking, emotion change recognition, voice vocalization, gestures, emotive expressions, singing, and dancing. | The majority of PwD experienced positive engagement when interacting with the robots. These PwD were very interested to engage in group activities, such as playing bingo, with Jack. |
| Chu et al., 2021 [57]  Canada  Qualitative | To design and develop a novel, user-centered, evidence-based exergaming system for use among OAs in LTC homes. In addition, we aim to identify facilitators and barriers to the implementation of our exergaming intervention, the MouvMat, into LTC homes according to staff input. | Long-term care facility | Exergaming, defined as a combination of exercise and gaming, has the potential to engage OAs in exercise and encourage social interaction. | The iterative use of validated scales (System Usability Scale, 8-item Physical Activity Enjoyment Scale, and modified Treatment Evaluation Inventory) indicated an upward trend in the acceptability, usability, and enjoyment scores of MouvMat over 4 rounds of usability testing, suggesting that identified areas for refinement and improvement were appropriately addressed by the team. A qualitative analysis of semi structured interview data found that residents enjoyed engaging with the prototype and appreciated the opportunity to increase their PA. In addition, staff and stakeholders were drawn to MouvMat’s ability to increase residents’ autonomous PA. The intended and perceived benefits of MouvMat use, that is, improved physical and cognitive health, were the most common facilitators of its use identified by study participants. |
| Cruz-Sandoval & Favela, 2019 [58]  Mexico  Quantitative | To assess the effectiveness of the conversational strategies for caregivers proposed in the literature during PWD-robot interactions:  1) Can the use of the conversational strategies increase communication between PWD and a social robot?  2) Can the use of conversational strategies make the interaction more enjoyable? | Geriatric residential care facility | We designed Eva as a semi-autonomous robot capable of handling simple interactions without human intervention, but requiring an operator to engage in more complex conversations. A web  application is used to operate the behavior of the robot remotely. Using this remote app, the human operator  can (1) send personalized utterances, (2) display emotions in Eva’s face, (3) trigger predefined activities  (greetings, jokes, farewell), and (4) search and play songs. | The number of utterances made from a people living with dementia to the robot increased significantly when the conversational strategies were included in the robot. In addition, people living with dementia engaged in more sustained conversations. Additionally, people living with dementia a enjoyed  conversing with the robot Eva, as much as listening to music. These results indicate that the use of these conversational strategies is effective at increasing the interaction between people living with dementia and a SAR. |
| Cunningham et al., 2019 [59]  UK Mixed Methods Cohort Study | To investigate the effect and impact of song-task association and musical reminiscence techniques, delivered via a mobile or tablet app, upon the wellbeing of a sample from the population of people living with dementia in UK residential care, as well as the care home staff supporting them. | Residential care facility | Main technology used was the Memory Tracks Mobile application. Memory Tracks is a care platform that utilizes music associated with daily tasks. It is a technology platform that supports those people living with dementia, those caring for them, and their families. The application aims to help trigger memory, manage agitation, assist with care, and support daily routines through the benefit of song-task association. In its present form, MemoryTracks is an Android app. | Quantitatively, The statistical test showed no significant effect, indicating that the use of the mobile app had neither a positive nor a negative impact upon the six combined indicators of participants’ wellbeing. Qualitatively, Residents may make a general association between the Memory Tracks app and the music that is played. This would be a general association that shows a positive impact and indication that a specific connection between a song and a task could be made. |
| Dahms et al., 2021 [60]  Germany  Pilot Study | This pilot study examined the frequency of use and acceptance of Music Therapy (MT) and technology-based music  interventions (TBMI) as well as the influence of high and low usage of  both interventions of dementia on behavioral and psychological symptoms (BPSD) at two timepoints. | Nursing home | Music therapy can be used as an alternative therapy and can function as  a medium for people with dementia (PwD) to engage more actively with their environment and to better express their emotions | Descriptive results showed higher frequencies of use and acceptance of the music interventions delivered during personnel-guided MT and TBMI (such as individual MT, group MT, and group music with movements) compared with the music interventions that were only technology based and played without guidance. This result seems to indicate that actively guided and individual-based music  interventions delivered by qualified personnel are better accepted by PwD than technique-based music interventions only. |
| Damianakis et al., 2010 [61]  Country not specified  Qualitative | To observe Alzheimer's disease (AD) and mild cognitive impairment (MCI) patients' responses to personalized multimedia biographies (MBs). We developed a procedure for using digital video technology to construct DVD-based MBs of persons with AD or MCI, documented their responses to observing their MBs, and evaluated the psychosocial benefits. | Residential care facility | Multimedia biography delivered via television. The family caregivers or participants used workbooks to structure the biography's storyline. We outlined criteria for selecting biographic content (e.g., stimulating memories and emphasizing personal qualities, strengths, and abilities; leaving a legacy for family members; generating an empathic dialogue). The workbook also helped families to structure content (a) within time periods (childhood, youth, education, career, middle age, life today), (b) with reference to personhood (personality characteristics, values, daily rituals, health history, losses or tragedies), and (c) with regard to interests, accomplishments, and social factors (family, friends, community, celebrations). For each biography, the participants made decisions whether to structure their storyline chronologically (childhood, adolescence, adulthood) or by significant life events (marriage, children, travel, etc.). In most cases, family members and the patients were filmed as they provided narration that located images and other assets of the MBs into the context of a story. | Triggered memories for AD participants, enriched memories for MCI participants. Both AD and MCI participants and their families had various verbal and nonverbal emotional responses to the videos with “enjoyment,” smiling or laughing most frequently observed. Families expressed the impact of the MB in facilitating intergenerational communication and in preserving a “family legacy,” further facilitating patient’s generativity or desire to give of oneself to future generations. |
| D’Cunha et al., 2021 [62]  Australia  Mixed methods | To evaluate whether the  Virtual Cycling Experience was physically safe and feasible and if it provided benefits over usual exercise activities on  mood, apathy and engagement. Also, to explore the perception of virtual cycling users and facilitators to identify benefits and concerns associated with it. | Residential aged care facilities | Virtual Cycling Experience (VCE) to evaluate whether the VCE was physically safe and feasible and if it provided benefits over usual exercise activities on mood, apathy and engagement. | Participants reported the virtual cycling experience to be immersive and challenging and reminisced about cycling earlier in life. The activity manager observed that the virtual cycling experience was an overall positive experience and emphasised  benefits of safety screening and preparation prior to the activities. The findings of this study support the use of the virtual cycling experience as an immersive and engaging alternative to usual activities, which might encourage higher levels of physical activity in residential aged care facilities. |
| Demiris et al., 2016 [63]  USA  Mixed Methods | To examine the feasibility of digital companion systems in real world settings used by older adults with mild cognitive impairment in their natural environment and for a longer period of time. For this purpose we utilized a system that is comprehensive in its functionalities (including conversation ability, use of pictures and other media, and reminders) to explore the system’s impact on older adults’ social interactions, anxiety and depressive symptoms, and participants’ acceptance of the system. | Home | The virtual pet companion is displayed on a tablet as part of the GeriJoy service provided by a company called “care.coach.” It interacts with the older adult through voice and expression. It stays plugged into a dock by the older adult’s bedside or other preferred location in their residence. On the other end of the device is a trained staff member of a 24/7 call center who listens to the older adult and types responses that are then converted to audible speech. Thus, the “pet” is fully controlled by a human who uses both scripted text and unscripted spontaneous exchange for all the interactive sessions with the end user. The call center staff summarize their interactions with the older adult and send a log to a family caregiver. The caregiver can also send pictures to the device to share with the older adult (see Figure 1). In this study, we provided older adults with the option to share this log with a family member, but it was not automatically transmitted to anybody besides the research team. The device allows the human on the other side of the tablet to see the older adult in their room (when the pet is “awake,” signified by its eyes being open) to determine if the older adult is interested in engaging in conversation and to add a dimension to the interaction. In this study, we allowed the older adult to opt-in to having this video feature | Overall, participants utilized the tool regularly and appreciated its presence and their interactions. Participants scored higher at the end of the study in cognition and social support scales, and lower in presence of depressive symptoms. There was an increase noted in both the MoCA and the MOS-SS, with the largest benefit seen, not surprisingly, in the positive social interaction subscale. There was a small increase in anxiety at study exit. We explored the association between change in anxiety and attachment to the digital companion (recognizing that such a sub-analysis is weakened by our small sample size). Interestingly, those with higher attachment to the pet had a baseline GAD of 3.75, increasing to 5 at end while those with lower attachment (not very/not attached) actually had a decrease on the GAD from 1.75 to 0. 75. |
| Dinesen et al., 2022 [64]  Denmark  Exploratory Study | This study aimed to explore how the social robot LOVOT interacts with persons with dementia and how health care  professionals experience working with LOVOT in their interaction with persons with dementia. | Nursing Homes | LOVOT is built with  artificial intelligence, which makes it move in real time and act  like a human being. LOVOT uses multiple sensors all over its  body, including touch and distance sensors. The touch sensors  are used to make LOVOT recognize stimulations on the body  and can be warm or cold; it can even “fall asleep” when a person  is stimulating the sensors. | LOVOT had positive effects, opened up communication, and  facilitated interpersonal interaction. Although LOVOT did not create noticeable effects on social well-being, it gave individual  persons a respite from everyday life. Some residents were overstimulated by emotions after interacting with LOVOT. |
| D'Onofrio et al., 2019 [65]  Ireland, Italy, & UK  Mixed Methods | 1) To illustrate the key results and evidence obtained in the final evaluation phase of the project across the three different pilot sites; 2) To assess the engagement dimensions of the PLWD who interacted with the MARIO robot;  3) To assess the acceptability and efficacy of the MARIO companion robot on clinical, cognitive, neuropsychiatric, affective and social aspects, resilience, quality of life in PLWD, and burden level of the caregivers | Residential care homes; Inpatient hospitals; Home | MARIO uses the Kompai 2 robot platform developed by Robosoft. It is a robot equipped with a camera, a Kinect, and two LiDAR sensors for indoor navigation, object detection, and obstacle avoidance. A tablet PC is located on the robot torso for inter-action that is loaded with apps, including a music app, a reminiscence app, news app, etc. | Attitudes toward MARIO were captured by the amount of excitement or expressiveness displayed during engagements with MARIO (smiling, frowning, energy or excitement in their voice). In Ireland the mean attitude scores towards MARIO, most of the time, ranged from 4 to 6 (5.52±0.55), indicating that participants had a positive attitude towards MARIO. In Italy, the mean amount of excitement or expressiveness displayed during engagements with MARIO(smiling, frowning, excitement in voice) range from 4 to 5 (4.64±0.10), showing participants were neutral to somewhat positive in their attitude toward MARIO during engagements. In the UK, the mean amount of excitement or expressiveness displayed during the three participants' engagement with MARIO ranged from 1 to 6.00 (5.29±0.15),indicating that participants' attitudes toward MARIO during engagements were conflicting. |
| Dove & Astell, 2019 [28]  Canada  Qualitative | To explore the use of motion-based technology (Xbox Kinect) as a group activity for people with dementia who attend an adult day programme. The day programme owned the Xbox Kinect and used it once a week as the basis of a group activity offered to the people attending on that day. Our aim was to gain a detailed under- standing of (a) the day programme clients with dementia, (b) the group facilitators, (c) the activity, (d) the environment and (e) the interaction between them. | Day care center | The Xbox 360 Kinect (Microsoft) and the bowling game from the Kinect Sports package (Microsoft) were used in this study. | Thematic analysis revealed three predominant themes relating to the use of motion-based  technology as a group activity for people with dementia who attend adult day programmes. The themes are (a) the importance of having a trained trainer, (b) learning versus mastery and (c) playing ‘independently together.’ |
| Evans et al., 2016 [66]  UK  Qualitative | To collect key information about the way in which personal message cards were used, including the goals that were set for them and the extent to which they would be perceived as being successful in meeting these goals. | Nursing home; Home | Personal Messages cards were created by carers of persons with dementia with the support of librarians and utilizing a commercially available talking greetings card, with a facility to record a short recorded audio message and insert photographs. | The way in which the cards were used was described by an art therapist who had worked with Val, whose husband Fred was in a nursing home. Val wanted to use the cards to let Fred know that she would see him soon, as she worried that when she was not with him Fred felt that she was never coming back. As Fred was a keen artist, Val put pictures of herself and Fred’s paintings on the card. She also incorporated a brief extract of Fred’s favorite piano music in the message. In her feedback, Val described the impact of the card ‘‘I played apiece on the piano and he enjoys it. He opens it and he understands it. There is a photo of me on the front.’’ Val described the reaction of Fred ‘‘his eyes light up...maybe it’s the music, but that's what it’s about...I am not sure that he recognises me, but when he looked at the painting he looked for a very long time.” |
| Faw et al., 2021 [67]  UK | To illustrate advantages of a user-centered, co-design  approach when developing VR experiences with community partners and older adults. | Not specified | VR is an increasingly-accessible and moderately-priced technology that can enable frequent exposure to symphonic  performances and provide access to those unable to attend live  events. VR head-mounted displays (HMDs) enable dynamic  immersion in digitally created experiences | Participants expressed their desires for enhanced experiences  in future VR interventions. PWD and CPs experienced greater  technology challenges and lower levels of immersion, indicating  that additional co-design research with these populations is  needed to produce to more effective interventions that attend to  their unique needs. |
| Ferguson et al., 2020 [68]  USA  Quantitative | To establish acceptability and safety of VR via a commercially available immersive headset for therapeutic recreation in hPLWD. | Hospice | VR headset (Lenovo’s Mirage Solo with Daydream Business Edition).  The VR experience was a short YouTube VR 360 beach scene video, a 3.5-minute video looped for up to 12 times. The empty beach scene video was a neutral immersive environment that did not involve crowds, sudden noises, or sudden scene changes. The participant could rotate his or her head 360 degrees and remain immersed in the scene. An audio recording of background beach noise was initiated from the researcher's phone speaker. | On average, participants wore the VR headset for 12.4 minutes (SD 11.6 minutes, range 0-31minutes). Study found VR to be generally acceptable in our sample of hPLWD, which is consistent with the literature.12 The “wear time” results seem to coincide with the findings from the perceived experience. Affirmation and enjoyment themes seem to denote relatively positive perceived experiences that support the use of VR in hPLWD. |
| Fields et al., 2019 [69]  USA  Quantitative | Hypothesized that (1) the use of a social robot integrated with a creative engagement activity (e.g., participatory theatrical arts intervention Shakespeare) will have a positive impact on the mood, loneliness, and depression of older adults; and (2) such positive impact will differ for participants who have dementia and those who do not. | Residential care facility | The intervention incorporated Shakespearean text, and the social robot, NAO, performing and concurrently encouraging the older adult to perform as a function of a participatory performance arts model. This study uses a human-robot model of participatory arts. | Depression, loneliness, and face scores significantly decreased across six time periods and these declines differed between people with dementia and those without dementia. In addition, only significant changes of depression before and after the intervention were found between persons with and without dementia. |
| Givon Schaham et al., 2020[70]  Finland  Mixed Methods | To describe the development of TECH, examine feasibility and satisfaction from users with MCI. | Home | TECH: ‘‘Tablet Enhancement of Cognition and Health,’’ a novel cognitive intervention utilizing touchscreen tablet application [mostly puzzle game apps] for cognitive self-training for people with MCI, facilitated by weekly group sessions. | Twenty-eight community-dwelling older adults with MCI participated in the study and received TECH. Participants attended at least 80% of group sessions, they self-trained a mean (standard deviation [SD]) 20.9 (7.2) sessions for 5 weeks, mean (SD) total training time of 24.4 (11.9) hours, 4.9 h/week. Very high satisfaction with the intervention was reported by 78% of the 23 participants who filled in the questionnaire. |
| Groenewoud et al., 2017 [71]  The Netherlands  Mixed Methods | To gain insight in how people with dementia feel about playing one-player casual games and how to make these games available to people with dementia. This study focuses on the experiences and views of the iPad games by people with dementia. | 2 day care centers and 5 small scale living facilities (residential care). | Ten existing iPad games and three new game prototypes were evaluated. The iPads were protected by a lightweight cover that allowed them to stand in portrait or landscape mode on a table or clients' lap. On off switch and volume switches covered to avoid confusion. The existing games included:  -Four traditional board and card games for the iPad (Shuffleboard or iSjoel,  Draughts, Dominoes, and Solitaire);  -One musical instrument app (Xylophone);  -Three interactive visual or sound apps (Tesla Toy, Line Art and Soundrop);  -One virtual fish pond (Fish  Line Art and Soundrop);  -One virtual fish pond (Fish Pond);  -One virtual pet (Talking Tom). | 177 observation sessions with 54 clients (88% with no previous computer experience). Users spoke positively about the game - need for achievement, self-esteem, sense of connection and belonging, identity, having something to do and admiration for the game. Negative experiences related to low self-esteem, annoyance and a sense of insecurity, mismatch between tame and clients' skills. |
| Hashim et al., 2015 [72]  Malaysia  Quantitative | To establish an evidence-base for the acceptability and efficacy of using multimedia digital life storybooks with people with dementia in care homes, in comparison with conventional life storybooks. | Home | myBook is a personalized digital memory book that is designed and developed with the elements that can help users enhance their reminiscence of the past and stimulate cognitive function. It consists of daily routine reminders, photographs and games. The system is installed on an android smartphone where the user will be able to access the application anywhere and anytime. | This result indicates that the patient is highly accepting of the application and feels that it is a useful tool to assist her in enhancing her reminiscence, stimulating her cognitive function as well as helping her with her daily activities. The user claimed that she felt motivated to use the application and seemed to be excited during the sessions. |
| Hebert et al., 2018 [73]  USA  Mixed Methods | The aims of the study were to: (a)explore the barriers to and facilitators of making IM (individualized music) available to LTC residents through a structured implementation process, guided by the PARiHS elements of evidence, context, and facilitation; and (b) describe IM use by LTC residents following implementation. The overarching goal of the study was to provide a comprehensive description of IM implementation. | Long term care facility | Personalized playlist on an MP3 player. | “Music reminds me of my love affairs and happy days.” “This music reminds me of washing dishes at my grandma’s house when we used to sing church songs.” “Music makes me feel closer to Jesus. I listen to it every night.” Validating resident identity and agency. Staff prided themselves in honoring the preferences and routines of residents and several actively joined the facilitators in developing playlists. As one resident shared, “My favorite music is tattooed on my heart; I wouldn’t want to live without it. Everyone has music that touches their soul. You just have to find it.” Having music based on their personal preferences readily available in their room facilitated resident agency and identity. As one resident stated, “We are the soldiers; we know what we want.” IM provides residents with an opportunity to listen to music of their choice, at a time of their choosing. One resident stated, “I like having the music right here where I can have it anytime. It’s like a muscle relaxant; it calms me down.” A person-centered approach was enriched by facilitating resident agency through access to a meaningful activity. |
| Hird et al., 2024  [74]  Japan  Mixed methods pilot study | To evaluate the acceptance, efficacy, and potential of a digital application (Aikomi) for cognitive stimulation in residents with dementia living in Japanese care homes. Furthermore, this exploratory study aims to collect data that will enable the delivery of AI-driven *personalized* cognitive stimulation and can ultimately improve communication between patients and their caregivers (care home staff, family, etc.). | Care home | Aikomi is a modular application developed by a multidisciplinary team of psychiatrists, digital health experts, engineers, data scientists, occupational therapists, and more. It is designed to deliver personalized cognitive stimulation by simultaneously collecting individualized data from patients (and their family) and applying it to AI machine learning models (future direction – AI element is still in the works). Ultimately, the content is delivered as an audiovisual “STIM” that promotes communication between the resident and their caregivers. Some users also received scent stimulation in addition to the audiovisual elements. | The study found that most patients were highly engaged and participated meaningfully with the intervention and there were zero incidences of negative reactions. Care home staff reported improved concentration and increased incidences of spontaneous communication beyond the patients’ norm during and after the intervention. There were no significant changes in the wellbeing of patients according to the Mental Function Impairment Scale (MENFIS). The type and amount of data collected varied between the patients but were all meaningful and can aid in the future development of the AI module of the program. In summary, Aikomi is a successful and effective application that is accepted by the studied patients and can help promote communication and engagement. The data collected by the platform is also meaningful and can be used to train machine learning models. |
| Hoel et al., 2022 [75]  Country not specified  Mixed methods | Aimed to evaluate the usability of the I-CARE system in terms of usefulness  and user-friendliness to inform a future, large-scale trial. | Homes | I-CARE is a tablet-based technology providing leisure activities  specifically designed for people living with dementia to do in tandem with caregivers. | I-CARE is a feasible tool to facilitate enriching experiences in dementia caregiving dyads. Important relationship outcomes for the participating dyads were enrichment in social interactions, facilitated communication, having a shared activity and relationship sustenance. |
| Hung et al., 2021 [76]  Canada  Qualitative | The purpose of this study was to answer two research questions: (1) ‘How do hospitalized  patients with dementia respond to the social robot PARO?’ and (2) ‘How can the social robot be used most effectively to support the needs of patients with dementia in the hospital setting?’ | Hospital inpatient | PARO, a robotic pet seal, was designed to provide emotional and social support for older people. PARO is one of the more used socially assistive robots, having been successfully utilized for dementia care in multiple countries since 2003. | Our findings suggest that patients with dementia in the hospital unit perceived the social robot PARO as helpful for supporting their psychosocial needs. This study increases our understanding of the experience of older people with dementia using PARO in hospital. |
| Jøranson et al., 2016 [77]  Norway | To systematically investigate behaviors seen in people with dementia during group activity with the seal robot Paro, differences in behaviors related to severity of dementia, and to explore changes in behaviors | Residential care facility; Nursing home | PARO is a social emotional robot shaped like a baby seal. | Participants with mild to moderate dementia paid significantly more attention toward Paro compared to participants with severe dementia. There were no observations of negative behaviors in the recordings. Conversations with Paro were correlated towards smiles and laughter towards others in the group. Paro could be a medium to increase social interactions, as added value from the group activity for those who are able to participate in activities together with others. |
| Kajiyama et al., 2007 [78]  Country not specified  Qualitative | To comment on the effectiveness of this procedure when family or formal caregivers are working with dementia patients. | Home | Reminiscence therapy that does in fact use both visual and auditory stimuli. It combines the presentation of old photographs and favorite music pieces of individuals with dementia, combined in personalized video channels via a television monitor. | The caregivers considered the intervention to be a positive experience for the AD patients and themselves; reported that AD patients were more engaged than usual; and in situations where the AD patients were agitated, said that AD patients were less agitated following the presentation, which persisted for some time. Care recipients displayed a high level of positive emotion and a low level of negative emotion during and after presentation of the music and pictures. |
| Kalantari et al., 2022 [79]  USA  Mixed Method Feasibility study | To evaluate the use of virtual reality (VR) for delivering interactive nature-based content with the goal of prompting active engagement and improving mood  states in older adults. | Virtual environment | The use of VR has liabilities, particularly in regard to its lack of tactile engagement and its inability to fully replicate the deep complexity and material interconnectedness of actual  organic environments. | The findings indicated significant improvements in “good” mood and “calm” mood dimensions after exposure to  the VR, as well as improvements in attitudes toward the technology. These positive outcomes were significantly greater for participants with physical disabilities compared to those without disabilities. |
| Kelly et al., 2021 [80]  USA  Mixed Methods | To evaluate the feasibility of PARO interventions for hospitalized older adults with dementia, determine the physiological effects, and describe participant social-affective interactions. | Hospital inpatient | The PARO is an 8th generation, FDA-approved, neuro-therapeutic device. With sensors for sound, light, temperature, touch, and posture, the PARO engages with individuals through movement of its tail and flip- pers, opening and closing of eyes, and sounds that are similar to a live baby seal reflecting emotions of surprise, happiness, and anger. | The PARO was favorably accepted for 212 (95%) of the 223 PARO interventions. |
| Khosla et al., 2021 [81]  Australia  Mixed Methods | To study the engagement and robot experience of older people with dementia while interacting with a social robot named Betty in the context of home-based care. | Home | Betty is developed in collaboration with NEC, Japan, specifically for emotional and intentional communication and interaction purposes. The upper part of these social robots contains two light sensors (each of which is a 120-degree wide-angle color camera), two microphones for speech recognition, and voice localization in the front. Its head has two axes of movement, nodding/panning ± 180 degrees and tilting ± 45 degrees. The embodiment of the interactional environment in the robot involves modeling of human characteristics like gesture, emotional expressions, voice, and motion. In each participant’s home, a robot is installed for about 3 months. | The results of this research show that social robots like Betty are useful to positively engage with the old people at their homes. The outcomes of robot experience show that most participants enjoy the interaction with Betty, and feel Betty’s usefulness and have no anxiety to interact with it. |
| Kim et al., 2020 [82]  USA  Case Study | To examine the effects of viewing a nature-based video with classical music on tranquility level among memory care residents with dementia. | Memory Care facility | classical music is generally considered to be the most effective because it often  incorporates soothing melodies resulting in peaceful and esthetic experiences. | Residents’ tranquility levels from both residents’ and staff’s perspectives showed significant improvement after the intervention compared to their baseline. |
| Koh & Kang , 2018 [83]  Korea  Quantitative | To investigate the effects of the PARO intervention on cognitive function, emotion, problem behaviors and social interactions in elderly people with dementia. | Residential care facility; Nursing home | PARO is a social emotional robot shaped like a baby seal. | The total score for social interaction significantly increased from 43.88±14.34 points before intervention to 53.40±14.22 points after intervention. Regarding the subdomains of social interaction, there was no statistically significant difference between the pre- and post-intervention measures of facial expression (t=-1.18, p=.312) and talking to PARO (t= -1.94, p=.070). In contrast, the levels of looking at PARO (t=-3.14, p= .017) and interaction with PARO (t=-3.05, p<.001) were statistically significantly increased. |
| Kontos et al., 2021 [84]  Canada  Qualitative | To explore the potential of dance to enhance social inclusion by supporting embodied  self-expression, creativity, and social engagement of persons living with dementia and their families. | Long term care and community settings | Sharing Dance Seniors is an innovative, community-oriented, dance  program that is intended to support social inclusion by making dance accessible to older adults with a range of  physical and cognitive abilities, including people living with dementia, and by emphasizing the importance of creative self-expression and prioritizing expressive capacities and social interaction. | The playful and imaginative nature of how persons living with dementia engage with dance and demonstrate how this has the potential to challenge the stigma associated with dementia and  support social inclusion. This underscores the urgent need to make dance programs such as Sharing Dance Seniors more  widely accessible to persons living with dementia everywhere. |
| Kosurko et al., 2022 [85]  Canada  Qualitative | To examine how the multimodal streaming (live, pre-recorded, blended in-person) of the Sharing Dance Older Adults program developed by Canada’s National Ballet School and Baycrest influenced social inclusion processes and outcomes in rural settings. | Community and Long-term Care | Multimodal streaming (live, pre-recorded, blended in-person) of the Sharing Dance Older Adults | Meaningful engagement in dynamic interactions in the dance was achieved by involving local staff and volunteers in facilitation of and feedback on the program and its delivery. Different streaming technologies influenced social inclusion in different ways: live-stream enhanced connectedness, but constrained technical challenges; pre-recorded was reliable, but less social; blended delivery provided options, but personalization was unsustainable. |
| Kouroupetroglou et al., 2017[86]  Ireland & Italy  Mixed Methods | To address:  1) How PWD, who are residing in long term care nursing homes and in hospitals, react to the presence of a companion robot, and  2) Whether PWD can interact with the companion robot using simple apps to accomplish tasks, such as listening to music, playing games, and reading news headlines | Long term care; Inpatient hospital care | MARIO robot: multimodal interaction combing touch and verbal input with visual cues on a screen on the chest of the robot and a text to speech system that speaks to the user; applications running on MARIO use a UI component that provides developers with an API that allows them to present a set of specific UI patterns. | The results show that the PWD who engaged in the above testing phase were accepting of MARIO and liked his appearance but the multimodal interaction combining verbal and visual cues was, in some cases, challenging. MARIO employs a conversational mode of interaction with the user and, through these dialogues, seeks to help them to identify and use applications as they like (play music, games etc.). In this initial round of testing, the interaction was designed based on a question spoken by MARIO accompanied with a set of options to select from. However, the fact that MARIO presents verbally all options in a series of questions seem to confuse the PWD, especially those in more advanced stages of the disease. |
| Kuot et al., 2021 [87]  Australia  Qualitative | To investigate how integrating personalized digital music playlists into daily care plans of residents living with dementia in a rural nursing home in South Australia (Australia) would influence behaviors, well-being and clinical management of the residents, the nursing home environment and culture, and the social interactions between residents, family members or relatives and aged-care staff. | Residential care facility | Personalized iTunes libraries of digital music playlists were created by the research assistant (GT) and registered nurse (TM) for use in the music intervention. | Three themes emerged: quality of life, personalized care and better aged-care environment. Personalized music positively influenced resident's behavior and well-being, social interaction and the workplace environment and culture, and served as a useful tool for personalized care. Personalized music program is an effective, low-cost intervention to improve quality of life and personalized care of residents living with dementia, staff well-being, and a workplace and culture in low-resourced or rural aged-care settings. |
| Lancioni et al., 2015 [88]  Country not specified  Mixed Methods | Assessing the effects of response-related music stimulation versus general (response-unrelated) music stimulation on positive participation of 11 new patients with Alzheimer's Disease. | Residential care facility | The technology used during the baseline and the active intervention sessions included a microswitch, a laptop computer, and an interface to connect the microswitch to the computer. During the active intervention sessions, the computer was used to (a) present a 15-s song segment after each microswitch response (successive responses allowed the patient to listen to the entire song available), (b) present a verbal reminder of the response for getting music if the patient did not have a new response within about 15s from the end of the previous song segment, and (c) record the responses and reminders. A new response was recorded only if it was carried out after the end of the song segment presented as a consequence of the previous response. | The results of this study indicate that the active (response-related) music condition was more effective than or as effective as the passive music condition (i.e., with six and five patients, respectively) in promoting positive participation. |
| Lancioni et al., 2015 [89]  Country not specified  Observation | Not specified. | Day care centers | Study 1 - technology used for music selection and activation included a laptop computer with sound amplifier, a microswitch, and basic software. At each step, four options were presented. Their first step involved classical music, folk music, male singers, and female singers. The options appeared as specific picture–word combinations in separate cells of the computer screen and each of them was scanned (lit) for about 4s and verbally identified in the process. The patients could select any option by activating the microswitch (a pressure device in front of them) while it was being scanned. Study 2 - The technology included a microswitch, a computer with sound amplifier, and basic software.; Study 3- The technology included a static bicycle’s pedaling unit, a microswitch, a computer with sound amplifier, and basic software. | All 3 studies found encouraging results - the technology was associated with "positive participation" which was described as smile and verbalizations. The patients of Study I learned to choose and activate their preferred music pieces. The patients of Studies II and III enhanced their performance of the target movements and increased their indices of positive participation (e.g., smiles and verbalizations) during the sessions. |
| Lazar et al., 2016 [90]  USA  Mixed Methods | To assess whether and how a multipurpose technology system designed for dementia care could benefit people with dementia in a memory care unit and the people in their care network, such as family members and staff. A secondary goal was to assess the feasibility and acceptance of such a system | Residential care facility | A commercially available computer system designed for older adults in community settings (iN2L Mobile FLEX Lite Package; It’s Never 2 Late). The system included: (1) a commercially available standard interface designed for use with older adults, as well as (2) a prototype interface designed specifically for use with people with memory impairment/dementia. The system included access to freely available web resources such as search engines as well as programs developed specifically for the system. Features of the unit include a touch-screen monitor which can be plugged into an external monitor. In addition, the unit can be wheeled from room to room and has a webcam, microphone, and speakers. It also comes with additional peripherals such as a video camera, hand/foot pedal for exercise and therapy, joystick, and headset. The height of the unit can be adjusted to allow the unit to be used by people seated or standing. | Staff and family members reported benefits for residents such as enjoyment, inter- actions and connections with others, and mental stimulation. Findings also highlight challenges such as technical and ethical concerns. |
| Lazar et al., 2016 [91]  Country not specified  Qualitative | To examine what it means to design for agency in online sharing involving individuals with dementia. | Residential care facility | Moments incorporates an interactive art frame with physical buttons that supports creating and navigating an art  portfolio. The system detects tagged physical objects and associates their meaning with pages in the portfolio. The  the therapist selected a subset to bring into each session portfolios of work (i.e., collections of images or other media). | Moments created opportunities for individuals with dementia to participate in digital social sharing of their art work with friends and family. |
| Leahey & Singleton, 2011 [92]  Canada  Qualitative | To examine the impact of an ADP recreation therapy on the focus and leisure engagement of an adult male with dementia. | Day care center | Nintendo Wii Bowling - participated at least once a week with a group of 3-5 participants over a span of nine weeks. | Participants learned and retained skills to participate in the bowling, and were able to transfer the skills to other leisure pursuits. Wife reported the home case was easier for her, that the husband was more focused and alert. |
| Leuty et al., 2013 [93]  Canada  Mixed Methods | To address:  1. What is the usability of ePAD for therapists who work with OAs diagnosed with mild-to-moderate dementia.  2. What is the usability of ePAD for OAs diagnosed with mild-to-moderate dementia in an individual art therapy context?  3. What features and functions should be improved in future versions of ePAD? | Not specified | Computer based art device - Engaging Platform for Art Development (ePAD). ePAD uses artificial intelligence (i.e., software that allows a computer to make human-like decisions) to monitor a client’s (i.e., an older adult [OA] with dementia) level of engagement in order that ePAD can actively engage the client in an art activity, using prompts should the client become disengaged. | OA participants reported high satisfaction with ePAD and its novelty. They stated that they enjoyed working with the device and were pleased with the art they were able to create. But they also expressed frustration with some of the functions and the prompts were distracting rather than effective. |
| Li et al., 2022 [94]  USA  Feasibility trial | This study examines the feasibility, acceptability, and safety of a newly developed cognitive-enhancing  Tai Ji Quan training intervention, delivered via remote videoconferencing, for older adults with mild cognitive impair‐  ment (MCI). | Not specified | Tai Ji Quan training approaches, which  primarily emphasize training participants to master Tai Ji Quan forms and movements, do not explicitly inte-grate the cognitively demanding features of this mind-body therapy into a multitasking exercise that could be  cognitively stimulating and mentally beneficial for the MCI population, which experiences increasing difficulties in performing dual-task functions in activities of daily living | 55%. Feasibility was demonstrated by the overall successful  online program implementation, with good fidelity, acceptable compliance (76%), and excellent retention (94%). |
| Liang et al., 2017 [95]  New Zealand  Mixed Methods | To study the affective, social, behavioral, and physiological effects of the companion robot Paro for people with dementia in both a day care center and a home setting. | Day care center; Home | PARO is a social emotional robot shaped like a baby seal. | There were statistically significant differences in affective and social outcomes between care recipients in the Paro and control group at day care. Care recipients in the Paro group not only showed significantly more positive facial expressions (t=-2.22; p=0.43), they also talked more to staff and researchers compared to those in the control group (t=-2.30; p=0.42). No significant differences in negative facial expressions and other social responses were observed between care recipients in either condition. The results are based on 13 participants who attended an average of 6.15 (standard deviation = 2.97) Paro sessions. |
| Mandzuk et al., 2018 [96]  Canada  Qualitative | To examine how an older adult living with dementia, delirium, or depression would respond to one-to-one personalized music sessions. | Hospital inpatient | Portable music player with personalized music library. | Nine out of ten of the older adults were engaged and responded positively to the familiar music. Their positive responses included eye contact, smiling, humming, singing, tapping of fingers, toe-tapping, and reminiscing. As the sessions progressed they developed a relationship with the recreation therapist. Even if the older adults were calling out prior to their session, they were calmer with no calling out while they were listening to music. Prior to the music, frequent behaviours included: restlessness, calling out, disinterest in their surroundings, drowsiness or sleep. During the music session, they smiled, sang, maintained eye contact, moved in time to the music, expressed gratitude, talked, closed their eyes, requested songs, or drifted off to sleep. |
| Masoud et al., 2021 [97]  USA  Qualitative | To give insight into the experiences of individuals living with dementia and family care partners who regularly attend Memory Cafés, and explores how these gatherings affect perceptions of social connectedness. | Residential care facility; Home | Memory Cafés, sometimes referred to as Alzheimer's or Dementia Cafés, are a widely implemented program that provide individuals living with dementia and their care partners an opportunity to socialize with others. These spaces, whether virtual or in-person, provide individuals living with dementia a place to socialize without fear of stigma or judgment due to behavioral symptoms of their diagnosis. Memory Cafés focus on the capacities that individuals living with dementia maintain, including the ability to connect with others, express themselves creatively, and participate in group activities. For care partners, these programs allow them to socialize with their family members, rather than just focusing on care-related responsibilities, and to connect with other caregivers. | Five overarching themes were identified from the interviews: (1) Reprieve; (2) What is still possible; (3) Connectedness; (4) Inclusivity; (5) Value added, with ten sub-themes supporting these main themes. In supporting the social well- being of families living with dementia, our findings indicate that virtual Memory Cafés can also address other practical needs, including the opportunity to learn through observing others in attendance, engaging in cognitively stimulating activities, and being connected to community resources. While findings reveal that virtual models can be effective in facilitating these added advantages, there seems to be a need for increased intentionality in the planning and implementation of these online programs in order to facilitate these benefits. |
| Massimi et al., 2008 [98]  Country not specified  Mixed Methods | To examine how novel “off the desktop” technologies may help remediate identity through the provision of an external aid to memory and conversation through an exploratory case study. | Home | In-home ambient display called Biography Theatre that cycles through music, photographs, movies, and narratives drawn from the patient’s past and current life. | Overall, scores on the psychometric tests indicate an increase in self-identity and a decrease in apathy. However, due to the single-subject case study design and lack of appropriate norms for measuring identity in patients with Alzheimer’s disease, we were unable to apply satisfactory inferential statistics. Overall, the experience of building the DLH was a positive one for the caregivers and Mr. H. The family appreciated the opportunity to reminisce about the past with their father. However, the process was time-consuming, and required commitment from members of the family in order to make a high-quality account of Mr. H’s life possible. Mr. H was, at times, fatigued by the process of reminiscing, and during some sessions, he would stand up and leave. |
| McCarron et al., 2019 [99]  USA  Mixed Methods | To ascertain (1) the feasibility and utility of the SSA, (2) whether the outcomes of SSA use suggest potential benefits for persons living with memory loss and their care partners, and (3) how study design components could inform subsequent RCTs. | Home | The Social Support Aid (SSA) is a mobile phone-based app that employs facial recognition software. It was designed to assist persons with memory loss remember the names and relationships of the people they interact with to promote social engagement. The technology consists of a mobile phone equipped with a facial recognition software app and a smartwatch. Up to 1000 individuals can be “enrolled” in the facial recognition app database. Enrollment includes typing an individual’s name and relationship to the person with memory loss into the app and taking pictures of the individual’s face from multiple angles. Once enrolled and in view of the mobile phone’s camera, the SSA app recognizes the individual’s face and alerts the smartwatch. The watch then vibrates and displays the individual’s image and text with their name and relationship to the person with memory loss. | Use of the SSA was not significantly associated with changes in quality of social interactions or quality of life measures over the 6 months of follow-up. The absence of empirical intervention effects is supported by the qualitative analysis, which revealed that the majority of participants did not find the SSA useful. Anecdotally, many participants mentioned they were not using the SSA, and a number of participants in the intervention group have contacted the study staff wishing to return the technology since the study ended. The qualitative analysis provides insight into why the SSA had few significant effects and provides recommendations for improving the technology. The majority of the participants interviewed did not feel their use of the SSA had any impact on the person with memory loss’s social interactions or quality of life. A few did note positive outcomes such as increased confidence and independence. Conversely, others mentioned negative outcomes such as increased frustration, agitation, tension between the caregiver and person with memory loss, and caregiver burden. |
| Merilampi et al., 2018 [100]  China  Qualitative | To introduce the developed activation game and its platform, and investigate its use as a self- managed activation and rehabilitation tool. | Residential care facility | The Heart Collection Game played on a gaming tablet with a Texas Instrument SensorTag game controller. Combines physical movement and cognitive stimuli - some light physical exercise | In general, the developed game received a warm welcome by the participants and nursing staff, and the overall feedback from the trial was positive. The developed game was seen as interesting, useful and evidently catching the participants’ interest. All participants were thinking the game is for wellbeing purposes instead of just for fun. This was commented to be one of the reasons for participation. All players commented that the game was useful for more than just entertainment and many players said that they would like to continue playing in the future. The game controlling handle was seen as convenient for gaming as the residents considered active use of both hands to be important. The game was also commented to be “good for the head” because of the cognitive stimuli. |
| Moon & Park, 2020 [101]  Korea  Quantitative | To evaluate the effect of digital RT and develop a strategy for larger RCTs, following convention. | Day care center | Digital reminiscence therapy. The app allows users to immediately conduct digital RT sessions onsite with pre-downloaded media and to add personalized material. Video and image files stored on the device or YouTube URLs can be added to the app. | Depression was significantly decreased and engagement was significantly increased at the last session in the digital RT group compared to the control group. However, cognition and BPSD were not significantly different between groups and time points. |
| Nijhof et al., 2013 [102]  The Netherlands  Mixed Methods | To explore:  1) What are the differences in behavioral outcomes between leisure games that do or do not use technology?  2) Which types of behavior occur during a CC session, and what are the differences in social behavior differentiated for MMSE and gender?  3) What are the experiences of activity facilitators with the CC in relation to their professional tasks, its usability, and the (observed) behavioral outcomes seen in PwD? | Nursing home; Day care center | The Chitchatters is a technology-based leisure activity that includes four interactive objects: a television, a radio, a telephone and a treasure box. Each of these objects trigger memories in its own specific way: the television shows videos, the radio plays music, the telephone tells poems or songs, and the treasure boxes reveal objects chosen as a source for reminiscence and promote tactile stimulation. The CC requires the participants to play while seated in a circle, in which the participants are surrounded by the objects. | Social behavior was found to occur more often than non-social behavior during the sessions, in particular, due to commenting during the game. Overall, participants made a lot of comments during the game, which is positive as they are actively involved in the game. Participants with a low MMSE score, scored higher for non-social and non-verbal behavior. Female participants scored higher for social behavior than males. Activity facilitators stated that the technology-supported leisure activity helps them with their professional tasks. |
| Obayashi et al., 2020 [103]  Japan  Quantitative | To determine the impact of age, gender and the stage of dementia on the results of an assistive technology intervention that makes use of communication robots (com-robots). | Residential care facility | Robot A: Cota. Speaks and encourages people to do certain tasks. Monitors people using infrared cameras, which sends alerts to caregivers and nursing stations in case of emergency or falls. Used to remind participants of scheduled activities. Robot B - Communicates and interacts more freely, with a greater degree of freedom and vocabulary. Robot B can perform music and lead physical exercises. | In each of the seven categories (Disability and health, communication, movement, self-care, domestic, interpersonal activities, performing tasks in a major life area, and tasks in social and civic life) when comparing the results from people at different stages of dementia, the study found that people with moderate/severe dementia showed greater improvement than those with mild dementia. |
| Olsen et al., 2000 [104]  USA  Qualitative | To systematically evaluate the inventions to determine if music and videos from the past were more effective in engaging clients, stimulating positive behaviors (such as smiling/laughing and interacting,) and inhibiting negative ones (such as agitation, wandering, sleeping etc.) than traditional day care activities. | Day care center | Music and videos were constructed in two custom designed Media Memory Lanes. The Musical Memory Lane (MML) was built in a 1930s Philco radio cabinet. The Vintage cabinet was deliberately chosen to trigger visual memory. The MML contains 12 tracks of nostalgic music. Individual tracks are activated by pushing a back-lit picture button that represents the theme of the track. The final selection contained a total of 28 songs presented in 12 different groupings. These included two tracks of big band melodies, two tracks of Broadway musicals (“The King and I,” and “Oklahoma”) and one track each of spirituals, Italian favorites, patriotic music, the Andrews Sisters, Rosemary Clooney, Kate Smith, Doris Day and sing-along medley. The Video Memory Lane (VML) operates on a simi-lar principle. It is housed in a 1950’s television cabinet and contains 12 tracks of nostalgic video clips from the movies and the early days of television. Each track is activated by pushing a picture button that represents its content. activity staff to stop a song or a video, initiate a discussion and then resume the piece. The operating systems in both Memory Lanes tabulate the date and time that each track is played. | The evaluation of the Memory Lane instruments indicated that they had a favor-able impact on engagement, stimulated positive affect and activity-related talking, while also reducing fidgeting. Finally, clients chose a Memory Lane activity significantly more often than other activities, and remained engaged with it for longer, during free time conditions. The findings that the MML was more engaging than live music and that the VMLs stimulated more discussion than the reminiscence activity were particularly striking. The highly significant degree to which clients chose a Memory Lane activity over other options during free-time was also a notable finding. However, the Memory Lanes were not particularly effective in reducing sleeping or stimulating general interaction. |
| Park et al., 2023  [105]  USA  Qualitative | To identify and assess the benefits, challenges, motivators, and considerations involved in telehealth-based chair yoga (CY) interventions in older adults living with dementia. This study takes these factors into consideration from both the patient and their caretaker’s perspective. | Home | The actual CY sessions were led by a certified yoga interventionist delivered via a 60 minute Zoom session on the patient or caretaker’s personal electronic device. These hour-long sessions occurred twice a week for 8 weeks (for a total of 16 sessions). During each session, both the patient and their caretaker were asked to participate; specifically, the caretaker assisted the patient in achieving the CY pose as instructed by the yoga interventionist. All poses were done either sitting or standing on a chair. These sessions were conducted in groups and patients and caretakers all had access to video feeds of other participants, which allowed for a sense of social connection. Furthermore, a ten-minute socialization period was implemented before the start of each session. | The authors classified their results into one of three themes:   1. Benefits: feasible and convenient, promoted better sleep, improved emotional regulation and mental health, improved concentration, better physical health and less pain, improved relationship between caregiver and patient, etc. 2. Challenges: the primary challenge faced by the participants was technical difficulties and confusion with Zoom – having a research assistant as live technical support helped the process significantly. Other challenges included attention span (participant unable to focus for the entire session) and socialization difficulties with a virtual platform. 3. Some of the considerations and lessons learned for the future include: importance of including the caregiver, having visual cues in addition to audio instructions, importance of promoting socialization, importance of having an actual yoga instructor live and present, and the fact that the online delivery provided a safe environment and setting, |
| Peeters et al., 2016 [106]  The Netherlands  Mixed Methods | To address:  1. How do participants respond to the music?  2. Are PwD and their relatives able to use the prototype?  3. What differences can be observed between participants?  4. What features were appreciated by the participants?  5. What additional features would be welcomed by the participants? | Day care center | App on a touchscreen tablet (Music ePartner) 1) managing playlists, 2) flipping through a music collection as a musical photo-album, and 3) listening to music while watching a photo slideshow. | We observed humming, singing, smiling, participant pairs making eye contact with each other and with the researchers, and all of the participants had stories to tell about the music. The most important effects of the Music ePartner seem to be: yielding a sense of recognition and safety, recalling old memories, creating shared experiences and evoking a positive mood. |
| Perugia et al., 2017 [107]  Spain  Mixed Methods | 1) To study the different engagement and arousal states that the two proposed activities brought about in people with dementia using observational rating scales and electrodermal activity;  2) To uncover correlations between participants' physiological states and observed engagement in activities | Residential care facility | Cognitive games were analogue;  Pleo robot: animatronic pet robot commercialized by UGOBE; appearance of baby dinosaur; equipped with touch sensors, microphones, ground foot sensors, force-feedback sensors, orientation tilt sensors, infrared mouth sensors, camera-based vision system, and a beat detection system; able to display a wide range of behaviors (e.g., walk), express internal drives (e.g., sleep), and moods (e.g., happy); provides a very prompt interaction which is important when working with PWD. | Results from OME and OERS show differences in engagement and affect between the two different activities. Cognitive games elicit significantly higher cognitive exertion (i.e. cognitive difficulty), whereas robot play elicits significantly higher positive affect (i.e. pleasure). These findings are in line with our hypotheses. However, in addition to the hypothesized effects, we found a significantly higher alertness of participants during cognitive games with respect to robot play. This might be due to the fact that cognitive games have a very precise flow and rules to attain at each step of the activity and consequently require participants to be always present and attentive. |
| Perugia et al., 2017 [108]  Spain  Quantitative | Two different studies aiming to reach a comprehensive inventory of behaviors accounting for engagement in dementia.  The first one is an exploratory study aimed at modeling engagement in cognitive board games. The second one is a longitudinal study to investigate how people with dementia express engagement in cognitive games and in interactions with social robots. | Nursing home | Pleo is an animatronic pet  robot commercialized by UGOBE, which has the appearance of a dinosaur. It is equipped with an array of sensors: touch sensors, microphones, ground foot sensors, force-feedback sensors, orientation tilt sensors, infrared mouth sensors, a camera- based vision system, and a beat detection system. Pleo is not only able to display a wide range of behaviors (e.g. sing, dance, walk, howl), but also to express its internal drives (e.g. hunger or sleep), and moods (e.g. happy, scared, curious). | Results revealed that cognitive games were perceived as significantly more difficult (M=2.07, SD=.730) with respect to interactions with Pleo (M=1.00, SD=.000, t(13)=5.491, p <.001). Moreover, they outlined that participants felt considerably more pleasure during social robot interactions (M=3.93, SD=1.141) compared to cognitive games (M=1.93, SD=1.141, t(13)=-5.508, p <.001), but were also less alert during interactions with Pleo (M=4.64, SD=.497) with respect to cognitive games (M=5.00, SD=.00, t(13)=2.687, p <.05). |
| Prophater et al., 2021 [109]  United States  Qualitative | The purpose of this project is to assess the effectiveness  of technology, specifically tablets, in reducing feelings of social  isolation and increasing mood among residents during the  COVID-19 pandemic. | Not specified | Project VITAL (Virtually Inclusive Technology for ALl) is  a unique project that leverages technology and community  resources to create a network for connection, engagement,  education, and support of individuals with ADRD and their  caregivers. This combination of components aims to positively  impact social isolation, stress, and well-being. | The tablets were shown to be an effective  way to engage residents and connect them with friends and family, as well as being a  useful tool for staff members. A |
| Šabanovic et al., 2013 [110]  USA  Mixed Methods | To evaluate the effect of PARO in the context of multi-sensory behavioral therapy in a nursing home. | Residential care facility | PARO is a social emotional robot shaped like a baby seal. | The significant takeaway is that the growth found in our analysis provides evidence of a particular modality of social interaction that PARO affects, which differs in PR and Non-PR participants. With non-primary participants, PARO served as a critical focal object in the room (evidenced by the growing incidence of looking at PARO) that correlated with vocalizations toward both PARO and other people present. For primary interactors, PARO appeared to enhance engagement with other participants, as evidenced by the greater increase in verbal interaction (toward PARO and others) than physical interaction (with PARO). In other words, even when the participant was directly holding or facing PARO, the principal effect over time was an increase in their interaction with other people. |
| Santen et al., 2020 [111]  Netherlands  Randomized controlled trial | To assess the cost-effectiveness of exergaming compared to regular activities from a societal perspective in day-care centres (DCC) for people with dementia and their informal caregivers (IC) alongside a cluster randomised controlled trial. | Day-care centers | An innovative form of physical exercise is exergaming, which  we define as physical exercise that is interactively combined with  cognitive stimulation in a gaming environment. Exergaming can make it easier for people with dementia to engage in physical exercise, because they do  not have to worry about becoming lost. | This study found that exergaming in DCC was not  cost-effective compared to treatment as usual for participants with dementia and informal caregivers regarding the primary outcomes of this study, i.e., QALYs, physical activity and mobility (based on SPPB scores). |
| Samuelsson & Ekström, 2019 [112]  Sweden  Qualitative | To further understand how digital communication support may be used in inter- action involving people with dementia. In the study, communication between persons with dementia and professional carers in conversations with and without the use of communication devices is examined. A further aim is to contribute information on how participants experienced communication with and without the use of communication aids. | Home | In this study, conversational activities based on two web-based applications, CIRCA (Computer Interactive Reminiscence and Communication Aid) and CIRCUS (Computer Interactive Reminiscence and Communication University of Sheffield), specifically designed to support inter- action involving people with dementia, were analyzed. Whereas CIRCA is populated with generic material that could potentially be of interest to “anyone”, CIRCUS is designed with an uploading function, and users will populate the application with their own materials. CIRCA is connected to a large database of pictures, videos, and music files belonging to six main categories of which three are randomly displayed in the beginning of a CIRCA session. | Overall, people with dementia were more active when using CIRCA and CIRCUS than during conversations without digital support. Increased activity of people with dementia leads to more symmetric interaction between participants, indicating that the conversational partner does not need to take a large part of the responsibility in the conversation as without the digital support. This view is also supported by the interviews made in this study, where conversational partners to people with dementia wit- ness that both CIRCA and CIRCUS were useful for stimulating topics and that they felt less pressure to uphold the conversations. The current study supports previous results arguing that digital communication support positively affects conversations involving people with dementia by facilitating for people with dementia to both take conversational initiatives and contribute with conversational topics. In this sense, conversations become more symmetrical, and the conversational responsibility becomes more evenly distributed, something that is arguably beneficial for people with dementia and the conversational partners alike. |
| Scase et al., 2018 [113]  Italy & UK  Qualitative | The aims of this study were two-fold: first to design a gamified environment through which applications could be delivered to promote cognition, exercise, social interaction and healthy eating; and second to test the adherence to this technology solution through an intervention where older people were asked to play the serious games over a 47 day period. | Home | DOREMI delivered a lifestyle intervention on a tablet computer and aimed to help participants improve their nutrition and increase physical activity, socialization and cognitive function and so encourage active aging. | Following the intervention phase 435 distinct sessions on the tablet computers were recorded for all participants with the number of sessions per participant varying considerably from 2 to 59 sessions of engagement with the cognitive games and/or exercise area. An independent-samples t-test was conducted to compare the number of sessions between the two groups of participants. There was a significant difference in the mean number of sessions for retirement village participants (mean=29.1, SD=14.8) and those living separately (mean=8.8, SD=7.5), adjusted t(14.3)=4.1, p=0.001. These results suggest that community living tends to promote adherence to the tablet-delivered intervention. The session length whilst participants interacted on the tablet computers ranged from 32 to 13611 seconds. There was no significant difference in the mean  participant session length between the retirement village participants and those living separately. |
| Sixsmith et al., 2010 (a, b, c) [114]  UK  Qualitative | To describe the development of a device to help people with dementia to be able to access and listen to music within their every-day lives and living circumstances. | Residential care facility | Modified mp3 music player with a small handle at the front of the lid and different graphics on the button. In addition a simple illuminated display was built at the back of the housing, which periodically displayed a prompt message and simple instructions to encourage the person to switch on the device. | Many of the residents visibly enjoyed having music on and were observed singing along, tapping the furniture and tapping their feet. The observed use of the device, the feed-back from participants and care-workers indicated a positive reaction. A key part of both initial use and continued use of these devices was installing music selected by the individual. Playing such music ensured that the device played personally meaningful music, making it more likely for an individual to use the player in the future. In two cases care workers noted that the participant was regularly listening to music via the player, and that appeared to be a change from their normal experience of life prior to the trial. In these cases, listening to their own music had previously reduced to a complete stop. However, as a result of installing the player, these two participants had regained an interest and desire to listen to the music. The introduction of music into the daily life of a person with dementia, was received positively by all participants to varying degrees. The device also acted as a prompt for social interaction and was seen as ‘sparking off’ interactions between residents and visitors in the home. |
| Smith et al., 2009 [115]  Country not specified  Qualitative | Not specified. | Home | Multimedia biography. We use the term multimedia biography (MB) to refer to a collection of personal media assets including photographs, film clips, audio narration, and music that are compiled in digital video format to tell the story of a life. We also often use the term digital life histories interchangeably to refer to MBs. The MBs in our project averaged 39 minutes in length and were delivered in DVD format. They were developed by members of our research staff in collaboration with the individuals with cognitive impairments and their family caregivers, and then they were viewed repeatedly by the participants with AD or MCI. | Participants and family members enjoyed both the production process and the series of screenings and reported that the MBs helped them reminisce and engage in stimulating conversations about past experiences. |
| Smith & Argentia, 2020 [116]  USA  Qualitative | To identify key issues with utility and appeal of specific electronic mobile apps for people with MCI;  To explore whether people with MCI could effectively use an electronic tablet as evidenced by little to no assistance in operating the tablet and playing the apps through observation. | Residential care facility | 10" ASUS tablet; consumer based cognitive training and physical activity apps found on the Google Play store: Elevate, Fit Brains Trainer, 7 Minute Workout, Sworkit. | Most participants agreed that Sworkit, Elevate and 7 minute workout were easy to use and fun, less frustrated, and would recommend to a friend; most participants had questions of clarification about how to play the game; all participants smiled and appeared happy when playing the physical games, whereas only 35% smiled during cognitive training activities. |
| Subramaniam & Woods, 2016 [117]  UK  Mixed Methods | To establish an evidence-base for the acceptability and efficacy of using multimedia digital life storybooks with people with dementia in care homes, in comparison with conventional life storybooks. | Residential care facility | Multimedia digital life storybook. Follows a chronological order from childhood until the current time, divided into six segments, e.g., childhood, teenage life, career, mid-life, etc., based on the photographs and other visual materials included in the conventional book, augmented with background music, participants’ favorite songs, and narration of the story. | Quality of life and autobiographical memory improved. All participants showed improvement or stability in depression scores. Not only did participants report the movie helped them in triggering their past memories, but this was accompanied by (largely) positive emotions and “feeling good”. Feelings of sadness were expressed, and some tears shed, but these were seen as natural expressions of loss, mitigated by the overall narrative of the life story. |
| Sweeney et al., 2021 [118]  UK  Qualitative | To address:  1. How do couples experience co-creating a digital life story book?  2. Does co-creating a digital life story book support couples’ well-being, and if so, what experiences support couples’ well-being? | Home | Digital life stories were completed using Book of You, a community interest company who provide a resource for creating a digital life story for individuals living with dementia (www.bookofyou.co.uk). The book can be accessed online via a laptop, tablet, or smartphone. | Whilst the process required more effort than expected, creating a life story was seen as a valuable way to formalize reminiscence; enjoyment and gratitude of a shared life together was often experienced not only in relation to reliving memories but also to the process of creating the book together. Couples reported laughing together when creating their life story books and humor was also present throughout the interview process; when difficult emotions were experienced and spoken about in a narrative of overcoming and resilience, positive emotions such as gratitude ensued. |
| Swinnen et al., 2023  [119]  Belgium  Mixed methods | While this feasibility study’s primary purpose was to determine the practicality and acceptability of an exergame prototype in patients with major neurocognitive disorder (MNCD), they also had secondary objectives of studying its effects on cognitive, motor, and quality of life outcomes. | Long-term care facility | The VITAAL exergame prototype is a novel and relatively simple intervention that can be run with limited technical expertise. It is a web-based intervention (the application is stored on a central server) that can be accessed by anyone with a laptop that has internet connection. The laptop is then projected onto a television screen for enhanced visibility and participants are required to wear two inertia sensors – one on each foot (these sensors are connected to the computer via Bluetooth). The actual exercises the exergame conducted included both classical strength exercises and Tai Chi-inspired exercises, the latter of which focus more on the lower limbs as these are mostly done in the semi-squat position. There were a total of 10 minigames (two are Tai Chi-inspired strength training games, four that targeted balance, two on cognitive training, one on task-switching, and one on short term memory training). Patients participated in three weekly sessions for 12 weeks, resulting in a total of 36 sessions. Each session consisted of 30 minutes of exergaming with five minutes of walking between the ward and the exercise room in which the exergames were conducted. | Thematic analyses of the interviews revealed three major themes: health effects, motivators, and barriers. In general, patients noted significantly improved mental health outcomes in line with that of traditional exercises. However, they found no significant changes in cognitive (including attention and memory) and physical health. Most patients expressed that they greatly enjoyed these exergames, found them to be safe, accessible, and not too difficult to understand and execute. The most commonly mentioned barriers and challenges to these exergames were unpleasant physical symptoms (such as pain) after squatting and that some of the older participants had a harder time understanding the instructions and performing them appropriately. Comparison of various quantitative test scores before and after the intervention revealed improved Mini Mental State Examination (MMSE) scores only. Quantitative tests looking at physical health, cognitive health, and quality of life did not reveal any significant changes. Overall, the authors concluded that the VITAAL exergame prototype was a relatively enjoyable and acceptable intervention that can improve mental health outcomes in patients with MNCD living in long-term care facilities and that future studies are warranted. It is relatively user-friendly and can be an effective intervention as long as participants are using it under guidance. |
| Tak et al., 2013 [120]  USA  Qualitative | To examine the feasibility of providing a 12-week computer activity program (CAP) for nursing homes residents with dementia. | Residential care facility | The activity content of CAP included sets of slideshows with music, and games. The slide shows consisted of a series of nature scenes (e.g., mountains, lakes, flowers) accompanied by calm and familiar musical pieces. Visual scenes designed to elicit positive emotion were selected from a pool of more than 3000 photos from the National Geographic Photo Gallery software (2003). The slide shows used songs from two music CDs (Reminiscing Moments and Quiet Moments from Music and Motion, Inc, 2003) that others had found effective for persons with AD. A computer technician created the slide shows using the Microsoft® PowerPoint program. Free or commercially available computer games included solitaire, tidy-up, balloon kaboom, blocks, jacks, pinball, and sticks that were considered appropriate for elders with dementia. | The findings suggest that it is important to match computer activities to interests and cognitive ability in order to increase participation and satisfaction of NH residents with dementia.  On average, participants completed 33 sessions amounting to 936.5 minutes over 12 weeks. Participants with mild and moderate dementia preferred playing a cognitively challenging game such as solitaire, while those with severe dementia enjoyed watching slideshows with music. The findings suggest that it is important to match computer activities to interests and cognitive ability in order to increase participation and satisfaction of NH residents with dementia. |
| Tak et al., 2015 [121]  USA Mixed Methods | To identify individual characteristics that are critical for the success of a tailored computer activity intervention among persons with dementia. Therefore, the purpose of this study was to examine participant’s experience and individual characteristics during a 7-week CAP for persons with dementia. | Residential care facility | A computer activity room was set up in a private room at each nursing home and equipped with laptop computers with touch screens and other accessories (e.g., mouse, trackball, touch pen, headphone, and printer). Computer activities included email, internet search, computerized games, and slideshow modules to promote cognitive stimulation and positive emotion as expressed in a visual and auditory manner. An email account was set up for each participant. The internet was used to search information that was interesting to participants. Various computer games that were free or commercially available were used: solitaire; balloon pops; tidy-up; block-match; greeting card-making (PrintMaster software); and a board and puzzle game collection (Galaxy software). | Each participant averaged 23 sessions and 591 min for 7 weeks. Computer activities included slideshows with music, games, internet use, and emailing. On average, they had a high score of intensity in engagement per session. Women attended significantly more sessions than men. Higher education level was associated with a higher number of different activities used per session and more time spent on online games. Older participants felt more tired. Feeling tired was significantly correlated with a higher number of weeks with only one session attendance per week. More anticholinergic medications taken by participants were significantly associated with a higher percentage of sessions with disengagement. The findings were significant at p < .05. Qualitative content analysis indicated tailoring computer activities appropriate to an individual's needs and functioning is critical. All participants needed technical assistance. |
| Tamura et al., 2004 [122]  Japan  Qualitative | To evaluate the effectiveness of an entertainment robot called AIBO in occupational therapy with demented patients and compare its effectiveness with a toy dog. | Long term care | The toy dog was a battery-driven toy that weighed 650 g and was 32 cm long from head to tail. The toy could wag its tail and sit, and was covered with thick polyester fur. AIBO was a metal entertainment robot that weighed 1500g and was 24 cm in length. It recognized and responded to up to 75 spoken commands. It communicated using four senses: touch, sight, hearing, and balance. | Experiment 1: patients responded with 985 different reactions to toy dog and 608 for AIBO; most frequent reaction was talking and watching; touching and caring reactions for toy dog and AIBO  Experiment 2: effect of dressing AIBO was not significant; watching and talking were frequent responses; care for AIBO increased on second and third days; few participants touched AIBO |
| Taylor et al., 2021  [123]  Australia  Mixed methods | To evaluate the user’s engagement in and enjoyment of digital musical instruments (DMIs) created using user-centred design approaches in older adults living in an aged-care home in Sydney, Australia. Secondarily collected feedback regarding potential points during which improvements could be made. | Care home | The research team developed a variety of proprietary gadgets and apps that would record physical gestural inputs from users. These were both physical devices with buttons or apps on mobile devices with touch interfaces. These recorders recorded gestures and translated them into musical outputs. The combination of the recorder and the musical output was referred to as a DMI. | Residents participated in bi-weekly group musical sessions playing and interacting with these DMIs under the supervision and guidance of music therapists in the presence of facilitators and care home staff. The study duration was 10 weeks for a total of 20 sessions, however, participants did not participate in all sessions (4 participated in more than 10 sessions). In general, the study found that participant enjoyment and engagement increased over the course of the study duration. Furthermore, improvements and changes made to the DMIs (both hardware and software) led to increased engagement (measured by the Music in Dementia Assessment Score – MiDAS) but providing feedback did not lead to significant increases in engagement. The researchers further found that increased complexity led to decreased enjoyment. This authors concluded that their user-centred DMIs were beneficial to residents of aged-care homes and that in general, they become more engaged and enthused the more time they spent with these DMIs. Furthermore, a simpler and less complex user interface may positively benefit participant enjoyment and engagement. Additional areas and points for improvement were also identified. |
| Topo et al., 2004 [124]  Finland, Ireland, Norway, & UK  Mixed Methods | To find out whether the program(s)was used, whether it was useful for the users and how it could be further developed. One general aim was to find possible associations between PG use and the well-being of the participants. During the first three weeks of use the data collection focused on impact on mood and social interaction. The main focus of the assessment was the actual use of PG and the outcome measures were closely connected to this. | Day care center | The Picture Gramophone (PG) multimedia program was designed to be used by people with dementia, to stimulate them and to give them pleasure. A new utility, ‘Editor’, was added to the original program to make it easier to develop individual PGs. The aim of Editor was that it should be fairly simple to use and that people with little experience of word processing and the use of Microsoft Windows could use it with a manual. When the Editor Program is used, a PC keyboard and a mouse are needed, but when the PG program itself is used, only a touch screen and the PC itself are needed. The PC needs to have a CD-ROM drive for playing music from a CD. | According to both the responses of staff members and the users’ own comments, the oldest users and users with severe dementia had more problems with using the PG than the other participants, but the difference was not statistically significant. No statistically significant difference was found in difficulties in use of the PG between men and women, between different diagnoses of dementia or use or non-use of medication in the study population. But those who did not have problems with the PG were also more likely to benefit from its use. According to staff, use of PGs had a very positive or positive impact on the mood of most participants in all countries except the UK, where two out of four users were reported not to have any impact ‘because they are happy people already’. Negative impact was reported in one person whose mood changed very quickly, but this participant was also said to benefit because sometimes the impact was very positive. In addition, PG use had a positive or very positive impact on most users’ social interaction in all countries except the UK where all the users were socially very active already. |
| Travers & Bartlett, 2010 [125]  Australia  Mixed Methods | To obtain feedback regarding experience with the radio program Silver Memories. | Residential care facility; Home | Silver Memories: a radio service designed for the older listener that broadcasts music and programs from the 1920s to the 1950s. | Influenced participants’ well-being and morale, had a relaxing effect on participants, including reducing agitation, reduced depression, increased energy, and looked forward to listening to the program. Some difficulties with the reception (e.g., static, signal dropping out) - bothered RAC participants more than community-dwelling participants. Chat shows are most frequently disliked followed by classical music and opera. |
| Tyack et al., 2017 [24]  UK  Mixed Methods | To investigate:  1. How does viewing art on a tablet-style computer impact the wellbeing of people with dementia?  2. What are informal caregivers’ impressions of this activity’s impact on the people with dementia they care for?  3. How does a person with dementia experience viewing art on a tablet style computer? | Day care center | Art viewing app on a tablet. | Well-being subdomains generally increased with number of sessions. Qualitative findings included changes in cognition (stimulating thoughts triggered through app), behavior (stimulated other activities, increased engagement with the arts), mood (enjoying sessions, or certain images boosting mood, although some images lowered mood), and relationships (provoked conversation, became a focus for joint attention, brought couples closer together). |
| Ulbrecht et al., 2012 [126]  Germany  Mixed Methods | To examine:  a) number of people who accept the games and  b) in which factors the participants who accept the exergames differ from those who do not accept them;  c) experience of therapists in introducing and supervising the games and  d) any potential evidence of effectiveness that might suggest this category of intervention is worthy of further analysis | Residential care facility | Wii game console, bowling, tennis, and darts; television-based. | Acceptance of exergames depends on cognitive performance - those with dementia were more likely to reject the games; people with mild dementia can experience exergames as a positive activity, but sufficient supervision is necessary; acceptance depends on practicability, relationship to participants' experiences with non electronic games (they were most responsive to the bowling game), and the variety of games |
| Unbehaun et al., 2018 [127]  Germany  Qualitative | To examine the impact of a suite of exergames that were expressly designed to support the daily life activities of PWD and their caregivers. | Day care center; Home | Technology is centered around a TV; system runs on a minicomputer that includes several exergames and is connected to a MS Kinect; a PlayStation 3 buzzer was used as a simple input device during games; training content included strength training and assessment, Balance and coordination, and Creativity and recognition. | Exergames affected the social life and environments of PWD on an individual and community level. PWD seemed to improve their self confidence as their body language improved. Overall PWD enjoyed using the system. It was used with grandchildren and informal caregivers to follow up on leisure activities that were previously set aside. Overall exergames was seen as valuable to PWD and positively affected and enhanced their daily life routines |
| van Santen et al., 2020 [111]  The Netherlands  Quantitative | To investigate whether exergaming has positive effects on performance of physical activities, mobility, physical, cognitive, emotional and social functioning, and quality of life as compared to activities usually offered to community dwelling PWD visiting day care centers. | Day care center | Interactive cycling using a stationary bicycle connected to a screen; while cycling, PWD sees a route on the screen; they can pick a route and it mimics the experience of cycling outside; day care centers had to buy or lease one of the following systems at a discount: DiFiets, Fietslabyrint, PraxFit or SilverFit Mile. | No statistically significant effects on primary outcomes of physical activity and mobility However, some small to moderate positive effects on cognitive and social functioning in PwD and small effects on distress and subjective burden and sense of competence of ICs were observed. There were clinically relevant effect sizes on physical activity of PwD, and their interest or enjoyment and perceived choice of physical exercise. This is the first study to show that exergaming for PwD indirectly also has positive effects for the ICs, that is, while the ICs do not perform exergaming themselves. No improvements were found on emotional functioning of PwD, quality of life of PwD and ICs, or positive care experiences of ICs. |
| Weybright et al., 2010 [128]  USA  Quantitative | Aim was to examine effects of Wii Sports bowling on attention to task and positive effect of 2 older women with MCI - participants were predicted to increase attention to task during the interventions and increase positive effects. | Residential care facility | Nintendo Wii Bowling & Wii Sports. | Attention to task and positive affect were documented by observing videotapes - by fixed eye gaze to the TV and active participation in the game through upper body movements in sync with the game and observing facial response in the form of smiles [cross culturally reliable]. Two interviews and three surveys were completed. Both women increased their focus and smiled during the activity - this was supported by their comments during the interview. |
| Yu et al., 2019 [129]  USA  Mixed Methods | To evaluate the preliminary efficacy of Memory Matters (MM) that was delivered one on one versus in a group format on mood, social interaction, QoL, and BPSD in people with dementia | Home | Memory Matters (MM), an iPad reminiscence game on mood, social interaction, quality of life, and behavioral and psychological symptoms of dementia. | Mood did not differ, except for apathetic mood between group MM and control arm at 12 weeks (P 5 .051). Social interaction improved for individual MM compared with group MM (t 5 2.38, P 5.017) and control (t 5 2.84, P 5.005) at six weeks, but not 12 weeks. Other outcomes did not differ. |
| Zamir et al., 2020 [130]  UK  Qualitative | (1) To assess the feasibility and acceptability of using video calls to conduct an intercare home quiz through Skype on Wheels (SoW) and/or Skype TV with older people living in care homes  (2) To determine whether non-familial social contact groups of the same age cohort are  useful in increasing socialization | Residential care facility | iPads & Samsung Galaxy tablets with Skype installed. | Documented observations and consistent feedback from care staff revealed the importance of ‘technology type’, ‘checking equipment’, ‘competitive activities’ and ‘peer interactions’ to ensure that the activity would be successful over a long period. As the sessions progressed, many of the same residents would continue to participate, but also fellow residents (from within the care home) would observe and decide to participate in the next session if they had not yet done so. This improved peer interactions within each care home to help build friendships and recruit residents to future sessions. Peer interactions across care homes improved vastly from session three to session eight as residents began to remember each other and engage in more meaningful small talk, for example, asking about each other’s families, their fashion and the way their care homes were different or similar. |
| Zamir et al., 2021 [131]  UK  Qualitative | To address a principal barrier toward implementation of a video-call intervention, ‘Skype on Wheels’(SoW). | Residential care facility | Our Skype on Wheels (SoW) device was intended as a ‘budget version’ with no robotics, moved around the care home by staff. The long-term aim of SoW was to improve socialization and reduce loneliness by helping residents to connect to distant loved ones and create new social contacts. | The results from this focus group study suggest that the interactive methodology employed enabled older people to describe and demonstrate what they preferred a new technology to look like. Dressing up the device using low cost materials improved residents’ understanding of what the technology was, improved the acceptability of a new technology, and increased the likelihood of the new technology being used in the near future. |
| Zhu et al., 2023  [132]  Taiwan  Quantitative quasi-experiment | Cognitive frailty is defined as a combination of physical frailty with cognitive impairment and is seen as a precursor to full dementia. This study aimed to determine how exergames could affect cognitive functions and loneliness in adults with cognitive frailty. | Community | An exergaming software (HappyGoGo) was run on a laptop connected to a television screen equipped with an infrared motion sensing device. The software ran a number of interactive games that were projected onto the TV where participants were able to engage physically and complete game tasks. The exergames were designed to utilise both upper and lower limb movements. Patients participated in two 40-minute sessions weekly for a total of eight weeks. During each session, there was an initial 5-minute warmup followed by the 30-minute exergame and a 5-minute cooldown. | The exergames performed by the intervention group were able to significantly improve cognitive functions (as assessed by the Montreal Cognitive Assessment – MoCA) but did not lead to significant changes in loneliness (as assessed by the Chinese version of the Loneliness Scale) compared to the control group. The study concludes with the suggestion to implement exergames in cognitively frail persons as an effort to improve cognitive function. |
