# Supplementary material for: Technology-Enabled Recreation and Leisure Programs and Activities for Older Adults With Cognitive Impairment: Rapid Scoping Review
Source: JMIR Neurotechnol. 2024 Aug 8;3:e53038. doi: 10.2196/53038 (PMC12671325; doi:10.2196/53038)
Supplement: Multimedia Appendix 4 [file neuro_v3i1e53038_app4.docx]

**Table 3: Target and Actual Sample**

| **First Author** | **Recruitment strategy (e.g., Where are participants recruited from? Sampling strategy?)** | **Is there a justification for inclusion criteria (Yes (Y)/No (N)?) If Y, *describe justification provided*** | **Other details of inclusion criteria** | **Other details of exclusion criteria** | **Total sample size (n=)** | **Age** | **Sex, n (%)** | **Ethnicity, n (%)** | **Cognitive Impairment** | **Other details of sample** |
| --- | --- | --- | --- | --- | --- | --- | --- | --- | --- | --- |
| Abdollahi et al., 2017 [44]  USA  Qualitative | Elderly individuals with dementia and depression living in the Eaton Senior Community in Denver, Colorado | N | Live alone, early-mid stage of dementia, availability for a period of at least 4 weeks to house and interact with the robot | *NR | 6 | Mean age 75  Range 63-86 | 5F, 1M | NR | Dementia | 3 were independent living and 3 were assisted living |
| Álvarez, 2022 [45]  USA  Prospective, single-anonymized, crossover-  group design | Participants were recruited from an assisted living community in Massachusetts. | N | Inclusion criteria were clinical diagnosis of AD or related  dementia with a stage 5–7 on the Global Deterioration Scale  which indicates moderately severe  to very severe cognitive decline, and capacity to tolerate social  interactions, musical stimuli, and testing. | Residents with very severe hearing impairment and with delirium or psychosis were excluded. | n=29 | NR | NR | NR | Dementia | 26 of them (89.7%) lived in the two floors of the  Memory Care Assisted Living (MAL) households, which are not  designated by level of dementia severity. Three residents lived in  the Traditional Assisted Living (TAL) community. |
| Appel et al., 2020 [46]  Canada  Mixed Methods | 4 locations in Toronto: Day Treatment Center at Baycrest Health, Runnymede Healthcare Centre, Kensington Gardens Health Centre, Dotsa Bitove Wellness Academy | Each study site had an appointed site research coordinator (RC), a healthcare professional (Registered Nurse, Activationists, Therapeutic Recreation Specialists) who would initially identify eligible participants and indicate to the research assistants (RAs) which individuals were interested. The RA would then explain the details of the study to potential participants and obtain informed consent. A shared decision-making process was employed when participants were not able to provide consent on their own, in which case both the participant and their substitute decision maker (SDM) were consulted, which occurred in 14/66 (21%) participants. | Over 18, spoke English, able to consent/have SDM who could consent for their participation | Vision impairment at a level that would impede the VR, open wounds or skin conditions on the face, or chronic neck pain/injury unable to wear VR headset | 66 | Mean age 80.5 | 60.6% F  39.4% M | NR | MCI | Thirty-nine percent of participants were married, 45% had a bachelor’s degree or Postgraduate degree. Nine participants wore hearing aids during the VR experience. Fifty participants reported wearing glasses for any purpose (near and/or distance correction) and seventeen of these participants chose to wear their glasses during the VR experience |
| Appel et al., 2021 [47]  Canada  Mixed Methods | Community teaching hospital affiliated with the University of Toronto in Toronto, Canada. Participants were recruited from the General Internal Medicine Department. | N | 65+, diagnosed with dementia, inpatient at the study site hospital | Open facial wounds, cervical conditions to make use of VR headset unsafe, or had no contactable SDM | 10 | Mean age 86.5 | 8F, 2M | NR | Dementia | Dementia severity ranged from mild (2/10, 20%), moderate (1/10, 10%), and advanced (4/10, 40%), with some unspecified (3/10, 30%). Half (5/10, 50%) the participants lived at home—3 (30%) lived alone, 1 (10%) lived with family members, and 1 (10%) had another arrangement. The other half (5/10, 50%) of the participants lived in senior housing—4 (40%) lived in long-term care or assisted living and 1 (10%) lived in a retirement home or independent living. The majority of participants (8/10, 80%) were not in a relationship—3 (3/10, 30%) were widowed, 1 (1/10, 10%) was single, 1 (1/10, 10%) was separated, and 1 (1/10, 10%) had another arrangement. Of the 10 participants, 2 (20%) were married |
| Assche et al., 2021  [48]  Belgium  Qualitative | This study was part of a multi-phase project called ReMIND with the specific goal of studying SARs. The authors first reached out to three independent living facilities that were already part of ReMIND and asked for verbal and written consent. A list of prospective participants were then drawn up from each facility by resident assistants there. It is worth noting that all participants were those that participated in a previous phase of ReMIND. | N | 1) already involved in a previous phase of ReMIND, 2) 70 years old or more, 3) have a score between 19-25 on the Montreal Cognitive Assessment (MoCA), 4) able to ambulate independently, and 5) living in an independent living facility | 1) insufficient Dutch language skills, 2) have a recent history of neurologic or psychiatric disorders, 3) severe vision impairment or hearing loss, and 4) health conditions that prevent them from participating in physical interventions | n = 4 | Median: 86 years  Range: 70-90 years | 2 males and 2 females (50% female) | NR | Mild cognitive impairment (MoCA 19-25) | 6 potential participants were contacted in the beginning with 1 refusing to join and 1 dropping out due to psychological difficulties with the COVID-19 pandemic |
| Astell et al., 2016 [49]  UK  Mixed Methods | Recruited from residential, specialist dementia and day care services. A member of the research team obtained consent from each participant. Members of staff from the care services supported the consent process as gatekeepers by identifying potential participants and providing them with an information sheet. The researcher only approached individuals if they were agreeable to finding out more information about the study. Those who consented were visited individually within their care service and the study was explained to them in full. | N | Living with dementia | NR | 30 | Median age 86  Range 78-100 | 25F, 5M | NR | Dementia | The severity of their cognitive impairment was assessed using the Montreal Cognitive Assessment (MoCA) with a score of <21 required to meet the criteria for dementia. The participants’ mean score on the MoCA was 13.4 out of 30 (range 8-21; SD 3). |
| Barrett et al., 2019 [50]  Ireland  Mixed Methods | The Director of Nursing (DON) in the nursing home or their nominee identified residents who fulfilled the study eligibility criteria, while also considering the potential benefit and burden that participation may have on the person. These residents were approached first by a member of care staff, who asked them if they would be interested in meeting the researcher. If they agreed, the care staff introduced the researcher to each potential participant. The researcher took time building a rapport with the potential participant and discussed the research with them using an information leaflet. The researcher returned after 1 week, at which time she re-oriented the potential participant to the research project and answered questions. | The consent process was undertaken in close liaison with the key nurse responsible for each person's care, and who was most familiar with each person's capacity to provide consent. | To be included in the study, participants must have resided in the nursing home for at least 1 month, be likely to remain in the nursing home for the duration of the study, and have a diagnosis of dementia, determined in any of the following ways: (a) formal diagnosis, (b) an assumed diagnosis assessed by a medical clinician, (c) prescription of a cholinesterase inhibitor medication, or (d) nurses' judgement and/or nursing records advising that the resident has dementia. | Residents were excluded if they had a significant sensory impairment or acute physical illness that, in the judgment of professional nursing staff, impairs their ability to interact with MARIO. | 10 | Mean age 83 | 7F, 3M | NR | Dementia | Eight of 10 participants completed the standardized questionnaires at 4-week follow up and were included in the pre-post statistical analysis. All 10 participants contributed to the MARIO bespoke questionnaires to some degree, as they all had at least one interaction with MARIO. |
| Benham et al., 2022 [51]  Country not specified  Exploratory study | Recruitment occurred at a senior centre through convenience sampling methods of flyer  distributions. | N | (1) members who attended the center at least  two days a week and (2) score at least 18 out of 30 on the MoCA (Montreal  Cognitive Assessment), as researchers suggest a score of 17 or less on  the MoCA is associated with possible Alzheimer’s Disease. (3) aged 55 years and older, (4) ability to travel to the site, and (5)  participation in the center programming without caregiver assistance. | self-reported history  of seizure, epilepsy, dizziness, vertigo, motion sickness, contagious skin  and eye conditions, sensitivity to light, and individuals who had hearing  aids, pacemakers, and/or defibrillators. | n=16 | NR | NR | NR | NR | NR |
| Berge et al., 2022 [52]  Country not specified  Mixed methods | NR | N | NR | NR | n=63 | Mean age: 82 | 62% were female | NR | Alzheimer’s disease | NR |
| Chen et al., 2021 [53]  China  Mixed Methods | This study was nested within a larger study that aimed to examine  the effectiveness of a tablet-based volunteer-mediated cognitive intervention for older people. The tablet-based training was provided by  trained volunteers living in the same community, and the trainer-trainee  dyad was carefully matched by registered social workers. | N | Inclusion criteria for participants of the program were: (1) being  older than 60 years of age; (2) living in the community; (3) cognitively  impaired, and (4) not having any neuropsychiatric behavioral problems. | NR | 57 | Mean age 86.69  range 76-98 | 44F, 13M | NR | MCI | The majority was female (77.2%) with limited education (average  years of education = 2.63, SD = 3.98) and living with family members  (29.8%) or living alone (29.8%). |
| Cheung et al., 2023 [54]  China  Clustered randomized controlled trial | The participants were PWD and their primary family caregiver who were referred to the researchers by the participating  collaborators, which provided community services to the neighbourhood. | N | 60 and above, community-dwelling, with cognitive impairment at stage 4 or 5 on the Global Deterioration Scale (early to moderate  dementia); (4) in a stable medical or mental condition to participate in group activities; (5) able to communicate in  Cantonese, the major Chinese dialect in Hong Kong; and (6) with a primary family caregiver who agreed to join the  programme. | Excluded were those who had suffered from any critical medical or psychiatric illnesses, had uncorrectable  visual and auditory impairments; were unable to sit independently for around 30 minutes; or who had participated in any  music intervention or clinical trial within the past six months. | n=100 | In the 70s | Male 48  Female 52 | NR | Dementia | The programme took place at seven collaborating centres and the participants’ home. To extend the reach of the service,  collaborators were not limited to those who provide community services to older adults but also included a women’s  services centre, and centres offering general community services. |
| Chidester et al., 2016 [55]  USA  Mixed Methods | Secured Memory Care Unit and Long Term Care residents | N | Assisted living residents with cognitive impairment who have limited access to social activities | NR | 62 | NR | NR | NR | Dementia | NR |
| Chu et al., 2017 [56]  Australia  Mixed Methods | Observation of the entire population of eligible residents in the facilities. | Observational to avoid researcher influence. | Suffer from differing levels of dementia and live in residential care facilities in Melbourne. | NR | 139 | Range of 65-90 | 96F, 43M | NR | Dementia | Sample measured over the course of 5 years: 2010-2014. |
| Chu et al., 2021 [57]  Canada  Qualitative | 2 LTC homes located in Toronto, Ontario, Canada. Various recruitment strategies included posters and direct enrollment by an LTC home staff collaborator. | N | LTC resident, 65+, English speaking, 10+ on the MMSE.  Collaterals were eligible if they were (1) a family member, substitute decision maker (SDM), or personally hired caregiver of an OA living in an LTC facility; (2) able to read, speak, and write in English; and (3) aged ≥18 years. LTC home staff were eligible if they were able to communicate in English and were currently working in an LTC facility. | NR | 13 residents  15 staff members & family | Mean age 82.23  Range 66-97 | 8F, 5M | NR | MCI | NR |
| Cruz-Sandoval & Favela, 2019 [58]  Mexico  Quantitative | Geriatric residence where all participants live. | N | Diagnosed with dementia, 70-90, able to speak, appropriate level of diction, adequate level of hearing. | NR | 12 | Mean age 80.25  Range 71-90 | NR | NR | Dementia | The MMSE scores of the participants (mean = 14.10, SD = 4.58) denote mild to moderate-stage dementia. However, we did not have total control over the attendance of the participants, due to factors such as absence, unavailability, and a visit from a family member. Since only 5 of them attended at least 80% of the sessions, our analysis focused on these 5 participants. |
| Cunningham et al., 2019 [59]  UK Mixed Methods Cohort Study | Participants were residents recruited from two care homes in the Pending Park group. | The selection of participants was undertaken with the advice of the staff at Pendine Park Care Organisation. In doing so, participants could be identified based upon their current physical and cognitive characteristics, including the level of dementia with which they are living, resulting in a convenience sample. | Participants were living with dementia, were typically between levels 5 and 6 on the Global Deterioration Scale for Assessment of Primary Degenerative Dementia, and were included in the study to reflect different types and stages of dementia | NR | 14 | Mean age 84.6  Range 69-97 | NR | NR | Alzheimer’s & Vascular dementia | Participants were mainly living with diagnoses of vascular dementia or Alzheimer’s disease and had a mixture of idiosyncrasies and conditions. In many cases, these could be problems with compliance in performing everyday tasks, such as washing, eating, and dressing, or in communication. In some cases, this could relate to being physically vulnerable and at risk of falling or could include mood swings and aggressive behaviours. |
| Dahms et al., 2021 [60]  Germany  Pilot Study | Over a period of 14 weeks, the pilot study was conducted in three  nursing homes with 30 PwD. Three nursing homes, two in Berlin  and one in Stuttgart, Germany, took part in the study (1. nursing  home in city area, no focus on specific diseases, 74 PwD in total; 2. nursing home in city area, focus on PwD with higher support  and security needs, 69 PwD in total; and 3. nursing home in city  area, closed gerontopsychiatry, focus on mental illness in old age,  69 PwD in total). | N | Inclusion criteria of PwD were a minimum age of 65 years, extensive independence (had to be able to participate independently in the musical interventions), and a clinically diagnosed dementia. | The exclusion criterion was a lack of verbal expression (should be  able to express music preferences or dislikes toward music songs). | n=30 PwD  n=9 RP | 52 to 97 | Female – 18  Male - 12 | NR | Dementia | NR |
| Damianakis et al., 2010 [61]  Country not specified  Qualitative | Social workers and other health care professionals from a multiservice geriatric care institution located in a large urban center referred 27 participants with AD or MCI over a 2-year period. | Although purposive sampling is not intended to provide generalizability of findings beyond the sample group, it is selected to ensure that specific characteristics of the population group are relevant to the overall study purpose and research questions. | Diagnosis of AD or MCI | NR | 12 | Mean age 79.6  Range 60-95 | 58% F, 42% M | NR | Alzheimer’s & MCI | 83% lived at home or with family member, 17% living in LTC facility |
| D’Cunha et al., 2021 [62]  Australia  Mixed methods | A single 72-bed high-care RACF (Residential aged-care facilities) in Canberra, Australia, was involved in the study. The lifestyle manager provided a list of all potential participants at the RACF who were believed to  be living with cognitive impairment and would be physically able to participate. Informed written  consent was provided by a family member, and informed verbal and/or written consent was obtained  from each participant. | N | If participants presented  with physical constraints or any of the aforementioned health conditions, they were eligible to  participate if the condition was stable but were identified for monitoring during the peri-exercise and  exercise period.  Participants in a wheelchair were eligible to participate. | Participants at high risk of injury or health complications, who were prone to  wandering, experience motion sickness, vertigo or dizziness, or those with self-reported eye strain  were excluded. | n=10 | Ranging from 75 - 95 | Female n=8  Male n=2 | NR | Dementia | In terms of mobility, 5 participants were walker-independent. 3 were walker-one assist. 1 was independe.t. 1 was in wheelchair. |
| Demiris et al., 2016 [63]  USA  Mixed Methods | Older adults were recruited through 1) posted flyers (at sites such as hospitals, memory clinics, day health programs, memory care groups and retirement communities with permission of those sites); 2) flyers sent to people in memory groups with the permission of memory group leaders; 3) word of mouth and snowball sampling; and 4) with the permission of memory group and day health program leaders, we approach participants directly and told them about the study using language similar to that on the flyers. Older adults interested in participating contacted a member of the research team. | N | Be able to see and hear well enough to interact with the device, reside in Seattle metropolitan area | Unwilling to be audio recorded, unable to speak English | 10 | Mean age 78.3  Range 68-89 | 100% F | 90% Caucasian10% Native American | MCI | One participant chose a family member to participate with them in the study. |
| Dinesen et al., 2022 [64]  Denmark  Exploratory Study | Participants enrolled in the study were diagnosed with dementia  prior to the study and before they moved into the specialized  nursing homes for persons with dementia. | N | Diagnosed with mild dementia.  Lives at 1 of the participating nursing homes in Aalborg, Viborg, and Skive Municipalities  Meets one of the behavioural criteria – lonely, high arousal, introverted behaviour | Refusal to participate.  Diagnosed with a neurological disorder.  Diagnosed with a psychiatric disorder | n=42 | 83 (67-92) 84 (66-96) | Male 1 (8) and 8 (27) (group)  Female 11 (92) and 22 (73) (group) | NR | Dementia | NR |
| D'Onofrio et al., 2019 [65]  Ireland, Italy, & UK  Mixed Methods | PLWD were consecutively recruited in three pilot sites: 1) Residential care (National University of Ireland, Galway, Ireland); 2) Hospital (Complex Structure of Geriatrics, Casa Sollievo della Sofferenza Hospital, San Giovanni Rotondo, Italy); and 3) Community (Stockport Metropolitan Borough Council, Stockport, UK). | N | 1) participants with diagnosis of dementia according to the criteria of the National Institute on Aging-Alzheimer’s Association (NIAAA) and the Diagnostic and Statistical Manual of Mental Disorders - Fifth Edition (DMS-5); 2) presence of cognitive impairment measured by MMSE, and 3) the ability to provide an informed consent or availability of a proxy for informed consent. | Serious comorbidity, tumors and other diseases that could be causally related to cognitive impairment (ascertained blood infections, vitamin B12 deficiency, anemia, disorders of the thyroid, kidneys or liver), history of alcohol or drug abuse, head trauma, psychoactive substance use and other causes of memory impairment | 38 | Mean age 77.08.  Range 55-93 | 24F, 14M | NR | Dementia | Education level ranged from 5-18 years. |
| Dove & Astell, 2019 [28]  Canada  Qualitative | Specialized adult day programme | N | Age related challenges such as significant mobility impairments or dementia that was beyond the mild stage. | NR | 23 | Mean age 77.7.  Range 63-92 | 13F, 10M | NR | Dementia | 16 had a diagnosis of dementia and 7 did not. 7 Had both cognitive and physical difficulty and 9 had just cognitive, 7 just physical. |
| Evans et al., 2016 [66]  UK  Qualitative | Bath Memory Technology Library. The product was then issued to the person affected by dementia and their carers by the librarian, who also provided instructions about its use. After a four-week period, the librarian contacted the carer to assess whether the product had been of use, and completed a review form | N | People affected by dementia and their carers | NR | 24 | Mean age 85.5.  Range 79-93 | 15F, 9M | NR | Dementia | 13 cards were used in nursing home with the rest being used by people living at home on their own or with others. |
| Faw et al., 2021 [67]  UK | Participants, recruited via convenience sampling using flyers  and in-person announcements at local senior living and  recreation centers, could not have a history of seizures or severe  motion sickness. | N | NR | NR | n=16 | 76 – 90 years old | 10 Females  6 Males | NR | Dementia | NR |
| Ferguson et al., 2020 [68]  USA  Quantitative | Using a descriptive design, we conducted a feasibility study with hPLWD receiving hospice care from a midwest hospice agency where N.M. is the medical director. The hospice agency is part of a larger health system with multiple levels of senior living environments. We recruited a convenience sample of n ¼ 25 participants. | N | Chart diagnosis of dementia and lived at one of the health system’s facilities or private home | NR | 25 | Mean age 85  Range 68-103 | 22F, 3M | 21 White  3 Black or African American | Alzheimer’s & Vascular dementia | Most participants were on at least 1 psychotropic medication at the time of the study and had reports of BPSD. |
| Fields et al., 2019 [69]  USA  Quantitative | Two residential care settings in a metropolitan area in North Texas. A staff member assisted with identifying participants who met inclusion criteria. The facility staff members spoke with all potential participants and their primary family caregivers and provided them with a brief overview of the study and background on the research team | N | 65+, living in a residential care setting. | NR | 15 | Mean age 85.7  Range 77-92 | NR | NR | MCI | NR |
| Givon Schaham et al., 2020[70]  Finland  Mixed Methods | This group is part of an ongoing single-blind randomized controlled trial assessing the effectiveness of TECH (experimental group) compared with a control group, for maintaining or improving executive functioning of older adults with MCI. Older adults were recruited from a health care provider, the study was approved by the Healthcare Helsinki Committee, and the University’s Ethics Committee and all participants provided written informed consent. | N | MCI of 19-25,subjective memory complaints by family member, independence in activities of daily living with normal or corrected vision and hearing, speak/write/read the language, able to use touchscreen tablet. | Experiencing severe depressive symptoms, diagnosed with dementia or other neurological or psychiatric conditions | 28 | Mean age 76.3  Range 65-87 | 13F, 15M | NR | MCI | NR |
| Groenewoud et al., 2017 [71]  The Netherlands  Mixed Methods | Two day-care centres for people with dementia and five small-scale living facilities from three health care organizations (Laurens, Aafje and Humanitas) in the city of Rotterdam, The Netherlands. Health care staff who saw the clients on a regular basis determined which clients were eligible. | N | Diagnosed with dementia, potentially interested in playing solo games on a tablet | Serious physical conditions including visual impairment, physical disability to handle the tablet, and severe apraxia | 54 | Mean age 83.5  Range 59.2-94.8 | 30F, 24M | NR | Dementia | NR |
| Hashim et al., 2015 [72]  Malaysia  Quantitative | The patient is suffering from a mild level of cognitive disease. The application was designed and developed specifically for her use. The contents of the application were supplied by her caretaker. The patient chose the pictures that she likes with the help of her caretaker. The application was tested in the living room of the house that she lives in order to provide her with familiar and comfortable feelings. | N | NA | NA | 1 | 74 | Female | Asian | Alzheimer’s | This is a case study of one person. |
| Hebert et al., 2018 [73]  USA  Mixed Methods | The study was conducted in the Community Living Center of the CGVAMC providing LTC and rehabilitation services. Purposively selected residents | N | All residents with a length of stay longer than 3 months. | NR | 3 | Mean age 79.67  Range 66-96 | 100% M | 66% White  1% Black | Dementia | NR |
| Hird et al., 2024  [74]  Japan  Mixed methods pilot study | The participants were recruited from 4 urban Japanese care homes. Potential participants were nominated by managers at each home to the research team for further selection. | N | 1) diagnosis of dementia, 2) display negative behavioural and psychological symptoms in dementia (BPSD), such as anxiety and apathy, and 3) permission provided from family for participation in this study | 1) having hearing or visual impairments that would prevent them from using a tablet, 2) having other mental conditions, and 3) diagnosis of frontotemporal dementia | n=15 | Mean age: 85  Age range: 76-93 | 4 male and 11 female (73% female) | Japanese Asian | Mixed dementia (87% from AD) | The 15 participants were sampled from a total of 4 urban care homes in Japan. |
| Hoel et al., 2022 [75]  Country not specified  Mixed methods | Caregiving dyads were recruited through  local health and care organisations, support groups and information centres in and around  Bremen, Germany. | N | NR | NR | n=9 | Mean: 77 | Seventy-eight per cent  of the care recipients were male, while 89% of the caregivers were female. 1 male with dementia and 8 females. | NR | Dementia | NR |
| Hung et al., 2021 [76]  Canada  Qualitative | Geriatric mental health unit of a large urban hospital in Canada. | Purposive sampling was used to identify patient participants to gather meaningful insights from a diverse group. We selected patients with different kinds of dementia, dissimilar functional disabilities, various ethnic backgrounds, both sexes, different ages, etc. Some participants were more fluent in linguistic expression; other participants had more communication and cognitive difficulties in social interactions. | NR | NR | 10 | 20% 60-75  60% 76-85  20% 85+ | 40% F  60% M | 70% Caucasian  20% South Asian  10% Black | Dementia | 20% in early stage  50% in middle stage  30% in late-stage dementia |
| Jøranson et al., 2016 [77]  Norway | NH units were recruited to the trial through the Centre for Development in NHs in three counties in Norway.  Thirty participants from five adapted units in NHs were recruited to participate in the Paro intervention, each unit forming a group | N | Nursing home residents with dementia | NR | 23 | Mean age 84.7  Range 62-92 | 69.6% F  30.4% M | NR | Dementia | NR |
| Kajiyama et al., 2007 [78]  Country not specified  Qualitative | NR | NR | Family caregiver-care recipient couples with dementia | NR | 23 older adults & 23 family member caregivers | NR | NR | NR | Alzheimer’s | NR |
| Kalantari et al., 2022 [79]  USA  Mixed Method Feasibility study | Using  fliers in local senior living centers and calls for volunteers  on community e-mail lists. | N | NR | NR | n=50 | NR | NR | NR | Cognitive Impairment | NR |
| Kelly et al., 2021 [80]  USA  Mixed Methods | Participants were recruited from the orthopedic, progressive  cardiac care units, and medical-surgical units at an 898 bed  acute-care urban hospital in the Southwestern part of the United  States. Patients were screened for eligibility over 63 days and all eligible were contacted. | N | 65 years  or older with documentation of dementia and/or delirium in the  electronic health record (EHR) and an anticipated hospital stay of  at least two days | Those unable to  physically or cognitively interact with the robotic seal, patients in  isolation and/or with open wounds, and patients with pace-  makers. | 55 | Mean age 85.5  67-104 | 69.1% F  30/9% M | 92.7% White  1.8% Asian  5.5% Unknown | Dementia | 100% had both dementia and delirium status on admission |
| Khosla et al., 2021 [81]  Australia  Mixed Methods | We firstly contact the manager of the aged care facilities in which the potential participants are regularly served. This helps to identify the potential participants. We then contact the family members of the potential participants and inform them of the research objectives and implications. Five older people with dementia who live with at least one family member (in the time of conducting the study) accepted to participate in this study. | N | 75-85 years old living with at least one family member | NR | 5 | 75-85 | NR | NR | Dementia | None |
| Kim et al., 2020 [82]  USA  Case Study | A Bachelor of Science nurse (BSN) recruited residents in long term care. | N | NR | NR | n=10 | 71 – 83 years old | 6 Females and 4 males | Caucasian | Dementia | NR |
| Koh & Kang , 2018 [83]  Korea  Quantitative | A long-term care facility located in J city, Korea | N | People with no mental illness other than dementia, a Korean mini-mental state examination (MMSE-K) score of 10~19 points, and no marked physical limitations on performing the program activities, who voluntarily agreed to participate in the study and signed the informed consent form | NR | 33 | 65+  Mean age 86.8 | 100% F | NR | Dementia, Alzheimer’s,  Vascular dementia | 58.8% of the sample was illiterate, 41.2% had education greater than elementary school |
| Kontos et al., 2021 [84]  Canada  Qualitative | Recreation therapists in collab-  oration with nurse managers facilitated recruitment by  introducing the study to residents living with dementia and  family carers and then connected interested family carers  with the Research Associate, who then obtained consent by  proxy for the residents | N | NR | NR | n=67 residents  n=15 carers | NR | Residents = 19 males and 48 females  Carers = 5 males and 10 females | NR | Dementia | NR |
| Kosurko et al., 2022 [85]  Canada  Qualitative | Community and long-term care settings. Participants were recruited in partnership with local Alzheimer Society support groups, Community Care Peterborough, and the Alzheimer Society of Canada Westman Region office in Brandon. | N | Older people, people living with dementia, and family carers (ranging from 66 to 96 years old); administrators and staff in both community and institutional settings; facilitators and volunteers supporting participants | NR | 289 | 66-69 | NR | NR | Dementia | N/A |
| Kouroupetroglou et al., 2017[86]  Ireland & Italy  Mixed Methods | IRELAND: : firstly, the Director of Nursing of the nursing home was contacted to discuss the project details to determine whether the residents and staff would be willing to participate in the study. Secondly, the Director of Nursing or her nominee identified those residents that would satisfy any of the conditions listed in the inclusion criteria while not falling into the exclusion criteria category, and whom they believed would be able to participate.  ITALY: NR | N | IRELAND: Resident at nursing home for 1+ month, formal diagnosis/any diagnosis of dementia by a medical clinician or nurse, prescribed anti-Alzheimer's medication  ITALY: 65+, Patients with diagnosis of mild dementia according to the criteria of the National Institute on Aging Alzheimer’s Association, the ability to provide an informed consent or availability of a proxy for informed consent | IRELAND: Diagnosed with severe dementia or memory loss, significant sensory impairment, acute physical illness that impairs ability to participate  ITALY: Patients with serious comorbidity, tumors and other diseases that could be causally related to cognitive impairment (ascertained blood infections, vitamin B12 deficiency, anaemia, disorders of the thyroid, kidneys, or liver), history of alcohol or drug abuse, head trauma, psychoactive substance use and other causes of memory impairment. | 10 | 65+ | NR | NR | Dementia | NR |
| Kuot et al., 2021 [87]  Australia  Qualitative | Nursing home in a small country town in South Australia. Ten residents living with moderate or advanced dementia were purposefully recruited by senior aged staff and quality assurance officer/senior registered nurse (TM), as previously diagnosed by their clinicians and recorded in personal care records, to participate in the personalised music program. | N | NR | Immobility | 10 | Mean age 81  Range 67-93 | NR | NR | Dementia | NR |
| Lancioni et al., 2015 [88]  Country not specified  Mixed Methods | They lived in residential social medical centers, in which interventions were largely focused on their personal care and basic forms of communication and engagement (e.g., television and prayers). Outside of these intervention/activity periods, most of them tended to be inactive and withdrawn in a situation of partial detachment. Their families fully supported the view of using stimulation sessions for them. Their families had also signed a formal consent authorizing their involvement in this study, which had been approved by a scientific and ethics committee | N | Not exclusively stated. 65+, diagnosed with Alzheimer’s, living in residential social medical centers | NR | 11 | Mean age 84  Range 65-95 | NR | NR | Alzheimer’s | 7 were diagnosed with severe Alzheimer’s and the other 4 were low-moderate. |
| Lancioni et al., 2015 [89]  Country not specified  Observation | NR | N | Diagnosed with Alzheimer’s, 65+ | NR | 3 | 67, 85, 65 | NR | NR | Alzheimer’s | Considered to function at mild/moderate level of Alzheimer’s with MMSE scores of 19, 22, 21 |
| Lazar et al., 2016 [90]  USA  Mixed Methods | Due to the residents’ cognitive status, those in the R group were not able to provide informed consent for themselves to participate in the research study. Therefore, letters were sent to the legally authorized representative (LAR) inviting study participation. Once LARs consented for their relatives to take part in the study, persons residing in the MCU were approached to discuss study procedures and obtain verbal assent. LARs and residents were also informed that they could choose not to participate or withdraw at any time without affecting care. LARs were asked if they were interested in being a part of the study (family member group). Staff were recruited through on-site information sessions. | N | Older adults in the R group had to be residents of the participating community, 50 years of age or older, and able to understand spoken English. Individuals were excluded if they were legally blind. Cognitive status of residents was not used as inclusion or exclusion criteria. People with any stage of dementia who resided in the MCU could participate in the study; however, weekly sessions were only held with individuals who appeared able to sit and focus for an hour. Family members had to be related to the resident, be aged 18 or older, have visited their relative residing in the MCU at least monthly in the year preceding the study, be willing to meet at the MCU for interviews, and be able to read and speak in English. Staff had to be aged 18 or older and interact directly with the individuals in the MCU. | Family members and staff were excluded if legally blind or had significant auditory impairments. | 5 residents, 4 family members, 7 staff | Residents: mean age 87.8  Staff: mean age 31.7  Family members: mean age 64.3 | Residents: 4F, 1M  Staff: 5F, 2M  Family: 3F, 1M | Staff: 4 Asian/Pacific Islander, 2 White, 1 Other  Family: 3 White, 1 multiracial | Dementia | NR |
| Lazar et al., 2016 [91]  Country not specified  Qualitative | Adults with dementia in an assisted living and memory care facility. | N | NR* No details of the dementia older adults sample are provided - only the prototypes they created for them. | NR | 8 | NR | NR | NR | Dementia | NR |
| Leahey & Singleton, 2011 [92]  Canada  Qualitative | ADP located in Nova Scotia that provided services Monday to Friday from 8:30 a.m. to 5:00 p.m. to clients who were either in early to mid-stages of Alzheimer’s disease or related types of dementia, physically frail (e.g., Parkinson’s Disease, stroke), or socially isolated. | N | Alzheimer's related dementia taking part in therapeutic recreation services offered as part of general programming of APD | None | 1 | 84 | Male | Caucasian | Alzheimer’s | Case study of 1 participant |
| Leuty et al., 2013 [93]  Canada  Mixed Methods | Sunny brook Health Sciences Center in Toronto. | N | Therapists: 2+ years of experience  Participants: 65+, mild to moderate dementia (MMSE score of 10-24), be physically able to participate, and currently participating in the art therapy program at Sunnybrook | NR | 6 therapists 6 older adults | 65+  Mean age 89.2 | NR | NR | Dementia | Participants had a mean MMSE score of 16.5 (range 15-25). |
| Li et al., 2022 [94]  USA  Feasibility trial | Initial enrollment efforts targeted  individuals who lived in Oregon but was expanded to  other states in the U.S. after the first wave of recruitment.  Multiple methods of recruitment were used, including  direct mailing, online promotions (e.g., research website,  social media), word of mouth, and contact with volunteers from previous research projects.  Recruitment was conducted primarily via initial  phone communication, | N | aged 65 years or older who met diagnostic criteria  for having MCI | NR | NR | Mean age 74.6 years (SD  = 5.6; range = 65-92 years old) | Female 16 (69.6) | 1.4% Hispanic 93% white | Cognitive Impairment | NR |
| Liang et al., 2017 [95]  New Zealand  Mixed Methods | 2 Selwyn Foundation dementia day care centers in Auckland New Zealand | N | NR | NR | 30 dyads of care recipients and their informal caregivers | 67-98 | 64% F  36% M | NR | Dementia | Caregivers age range 30-86, 96% female |
| Mandzuk et al., 2018 [96]  Canada  Qualitative | Mental Health program at acute care facility at St. Boniface Hospital in Winnipeg, MB. | Program referrals were made by staff or families. | 65 years of age or older with dementia, delirium, or depression, as diagnosed by their physician or those who seemed like they would enjoy the music sessions. | NR | 10 | Mean age 81 | 50% M  50% F | NR | Dementia | 90% Dementia but other reasons to include persons were depression, anxiety, loneliness |
| Masoud et al., 2021 [97]  USA  Qualitative | Participants were recruited using attendance sheets collected from three Memory Cafés in the Texas Memory Café Network (TMCN). An invitation with study details was emailed to attendees of the three Memory Café sites. Eligibility was verified for those who responded with interest to participate in interviews. | N | (1) self-identified as being a person living with dementia or a care partner to a person living with dementia; (2) exhibited capacity to consent to the study and discuss their experiences in interviews; (3) had attended at least two Memory Café events; (4) could participate over the telephone or video conferencing; and (5) spoke either English or Spanish. | None | 17 | NR | 64.7% F  35.3 % M | NR | Dementia | 88.2% English speaking  11.8% Spanish speaking |
| Massimi et al., 2008 [98]  Country not specified  Mixed Methods | He attended a bimonthly memory clinic at a local hospital and was referred to the study by a clinical neuropsychologist working at this hospital. | N | NA | NA | 1 | 84 | Male | White (British) | Alzheimer’s | Case study of one patient |
| McCarron et al., 2019 [99]  USA  Mixed Methods | Individuals with dementia, memory loss, or memory concerns, as well as their caregivers, were recruited from the University of Minnesota Caregiver Registry (a registry of caregivers who gave permission to be contacted about opportunities to participate in research), the Minnesota State Fair, and through statewide newspaper advertisements from February to October 2017 | N | (1) ability to fill out a survey in English or Spanish; (2) 21 years or older; (3) diagnosis of dementia or mild cognitive impairment, or has a self-identified memory concern (or a caregiver of such an individual); and (4) person with memory loss has sufficient cognitive capacity to provide verbal informed consent | None | 48 older adults  35 caregivers | Mean age 74.9 | 52% F  48% M | 36 (84%) White  2 (5%) Hispanic  1 (2%) Asian  3 (7%) Other race | Dementia | 68% Married  6% Divorced  17% Widowed  6% Separated  2% Never married |
| Merilampi et al., 2018 [100]  China  Qualitative | Three different elderly homes in Changzhou, China | N | NR | NR | 25 | NR | Male and female but NR | NR | MCI | NR |
| Moon & Park, 2020 [101]  Korea  Quantitative | It took several months to recruit participants and obtain consent from their family members because there are few large-scale daycare centers in the two local study areas in Korea, and only a few PWD in each daycare center met the inclusion criteria of this study. Ultimately, 251 candidates from nine daycare centers in two local areas in Korea were screened, 49 participants were enrolled, and 24 and 25 participants were assigned to the intervention and control group, respectively, from February to June 2019. | Justification for recruiting only female participants was included: “because meaningful memories based on familiarity and implicit memory are related to life habits or familiar skills and vary by gender” and the fact that free trials exist already that are based on a single gender sample | Female, 65+, moderate dementia, no impairment of hearing or vision, registered at the daycare center for more than a month | If they changed medication during the intervention period and were diagnosed with a psychiatric disease, except for depression | 49 | 65+  Mean age 83.46 | 100% F | NR | Alzheimer’s, Dementia | 47.4% diagnosed with Alzheimer’s  15.8% diagnosed with vascular dementia  36.8% diagnosed with other diseases |
| Nijhof et al., 2013 [102]  The Netherlands  Mixed Methods | A total of 21 out of 196 PwD residing in the nursing home or visiting the day-care centre were selected to participate in the study because of their capability to play both activities (this judgment was made by the head of the activity facilitators). A total of ten persons were observed (because of the possibility to observe only three or four persons at a time by the researchers). A responsible relative agreed for the participants to participate in the study (informed consent). In total, five participants from the day-care group and five participants residing in the nursing home participated in this study. | N | NR | NR | 10 | Mean age 69  range 52-86 | 6F, 4M | NR | Dementia | MMSE scores ranging from 3-28 with a mean of 18. |
| Obayashi et al., 2020 [103]  Japan  Quantitative | Four nursing homes and two rehab facilities for older people in Japan | N | Whether they were able to express their will, show some interest in com-robots and conduct two-way communications with their carers and family member | None | 78 | Mean age 86.5  Range 67-103 | 68F, 10M | NR | Dementia | NR |
| Olsen et al., 2000 [104]  USA  Qualitative | Senior Care and Activities Center in Montclair, NJ, USA | N | NR | NR | 15 | Mean age 82  Range 76-94 | 12F, 3M | 2 African American  13 Caucasian | Dementia | Of the original 25 clients recruited for the study, only six lasted to the end of the study three years later. This created an attrition rate of 76 percent. |
| Park et al., 2023  [105]  USA  Qualitative | Prospective patients were recruited from the Memory Disorder Centre (MDC) – a diagnostic facility for memory disorders in Florida, USA. Research personnel and facility staff approached prospective patients and provided them with verbal information in addition to flyers and interested caregivers or family members were asked to contact the research team. | N | 1) over 60 years of age, 2) lives in the community (not hospital, care home, etc.), 3) have a formal dementia diagnosis, 4) have a Montreal Cognitive Assessment (MoCA) score of less than 26, 5) can ambulate a minimum of 30ft safely and independently, 6) have a family member or caretaker that can accompany them during the CY session, and 7) have a personal electronic device (computer or tablet) that has access to the internet | 1) have any psychiatric disorder, 2) has an alcohol or drug dependency, 3) has a serious comorbidity that prevents them from participating in yoga, 4) requires constant assistance for ambulation (minimal is acceptable), and 5) uses a wheelchair | n = 17 (8 patients and 9 caregivers – all are stakeholders that participated in data collection) | Mean age: 81  Range: 68-96 | 5 males and 3 female (37.5% female) | 7 (87.5%) Caucasians and 1 (12.5%) African American | Mixed dementia (AD or dementia with Lewy bodies) | Important to note that this was a *qualitative* study where data were collected via focus groups consisting of stakeholders (both patients and caretakers were involved) |
| Peeters et al., 2016 [106]  The Netherlands  Mixed Methods | Five PwD were recruited through Pieter van Foreest care facilities: an organization that provides tailored person-centered healthcare solutions at more than 25 locations in the Netherlands. The PwD were approached by the program coordinators of the  meeting centres. Each PwD participated in the research together  with a close relative . | N | Dementia, older adult | NR | 10 | 50s-80s | All 5 PwD are male and accompanied by daughters or wives. | NR | Dementia | 5 pairs of PwD and close relative so demographics vary. |
| Perugia et al., 2017 [107]  Spain  Mixed Methods | Participants were selected from two nursing homes in the province of Barcelona. Selection was performed together with the psychologists of the two nursing homes. Dementia severity was assessed using the Reisberg Global Deterioration Scale (GDS) | N | Confirmed dementia diagnosis and deterioration level ranging from mild to moderate | Diagnosed Bipolar or schizophrenic disorder, abnormality in the movement of face or hands (ex. Parkinson’s), strong hallucinatory states, bedridden | 14 | Mean age 83.93  Range 69-92 | NR | NR | Dementia | NR |
| Perugia et al., 2017 [108]  Spain  Quantitative | NR for Study 1  Study 2: Residents of two nursing homes, performed together with psychologists of said nursing homes. | Study 1: The decision to include exclusively people with mild and moderate dementia in the study stemmed from the need to create a rich inventory of behaviours strictly connected with the engagement in the activity. In severe dementia, some behaviours could appear during sessions (e.g., sleeping) that are due to the progression of the disease, and not just to the engagement state of the person with dementia. Once a comprehensive inventory of behaviours is created for people with mild and moderate dementia, it could be scaled down to become apt to score engagement in severe dementia, but the opposite is not possible. | Study 1: a score of 4 or 5 on the Reisberg Global Deterioration Scale and a score ranging from 10-23 at MEC.  Study 2: Same as 1 | Study 1: bipolar or schizophrenic disorder, abnormality of face or hands, bedridden, strong visual impairment  Same for study 2. | Study 1: 8  Study 2: 14 | Study 1: Mean = 81, Range 69-92  Study 2: Mean = 84, range 69-92 | Study 1: 6F, 2M  Study 2: 12F, 2M | NR | Dementia | NR |
| Prophater et al., 2021 [109]  United States  Qualitative | 600 personalized Wi-Fi-enabled iN2L tablets were  distributed to 300 senior care communities (55% assisted living communities, 37%  skilled nursing communities, 6% memory care communities, and 2% adult family-care  homes) to connect and engage residents and their families. | N/A | N/A | N/A | N/A | N/A | N/A | N/A | Dementia | N/A |
| Šabanovic et al., 2013 [110]  USA  Mixed Methods | Senior living community in Bloomington, IN, USA. Our participants were recruited from the facility’s rehabilitation wing and all had some level of cognitive impairment, from minor to severe | N | Cognitive impairment | None | 7 | NR | NR | NR | Dementia | NR |
| Santen et al., 2020 [111]  Netherlands  Randomized controlled trial | Recruited a convenience sample of psychogeriatric Digital Competence Centres (DCC) across the Netherlands. | N | Inclusion criteria for participants with  dementia were: a diagnosis of any type of dementia, all ages, community-dwelling and not expected to be admitted into residential care in the next 6 months, visiting the DCC at least twice per week, and an informal caregiver willing to participate. | NR | n=73 | Mean 79.0 | Male 37  Female 36 | NR | Dementia | NR |
| Samuelsson & Ekström, 2019 [112]  Sweden  Qualitative | NR | NA | Dementia | None | 6 | NR | 100% F | NR | Dementia | 3 Swedish speaking dyads of older women with dementia and their professional carers |
| Scase et al., 2018 [113]  Italy & UK  Qualitative | Participants aged 65-80 years old with mild cognitive impairment were recruited to be part of the study. | N | Age 65-80 with mild cognitive impairment | None | 25 | Mean age 75  Range of  65-80 | 3M, 22F | NR | MCI | NR |
| Sixsmith et al., 2010 (a, b, c) [114]  UK  Qualitative | Phase 2 recruited from two residential facilities for people with dementia in South Yorkshire, UK. | NA | Cognitive impairment | NR | Phase 1 = 26  Phase 2 = NR  Phase 3 = 10 | NR | Phase 1 = 18F, 8M  Phase 2 NR  Phase 3 = 8F, 2M | NR | Dementia | NR |
| Smith et al., 2009 [115]  Country not specified  Qualitative | Our participants included individuals in various stages of the progression of AD and MCI. Social workers and other health care professionals referred 27 potential participants to us. Thirteen declined due to personal reasons or the required time commitment. Of the 14 participants, 12 completed the MB production process (1 family did not have time to continue, and 1 participant passed away). | Because the design of MBs involved participants with dementia and often their families, participant selection was an initial design decision of great importance. | Inclusion criteria were that participants were diagnosed with MCI or AD (preferably early to midstage) and had an extended interest to participate. | None | 12 | Mean age of 85  Range  60-95 | 8F, 4M | NR | Alzheimer’s, MCI | On average it took 5.6 months to produce each MB for the sample. |
| Smith & Argentia, 2020 [116]  USA  Qualitative | NR | NA | People with mild cognitive impairment | NR | 16 | 65+ | NR | NR | MCI | NR |
| Subramaniam & Woods, 2016 [117]  UK  Mixed Methods | The participants were recruited from the 23 participants in a recently completed randomized controlled trial where two approaches for developing a conventional life storybook were compared. | Participants for the current study were selected on the basis that they had given positive feedback on their book, with equal numbers having produced a book through the life review process and having received it as a gift. | Mild to moderate dementia living in care homes. | None | 6 | Mean age 82.2  Range  73-90 | 4F, 2M | NR | Dementia | 5 participants had a CDR score of 1, 1 participant had a score of 2. |
| Sweeney et al., 2021 [118]  UK  Qualitative | Participants were recruited from the National Health Service (NHS), which is a publicly funded healthcare system in the United Kingdom, and community dementia support settings via posters, word of mouth through staff and other service users and via the online Join Dementia Research (JDR) database from the North of England. | N | Both participants had to live in the community together, diagnosis of MCI, having a spouse or partner involved in their care, internet access, age 60+ | Lack of fluency in English, not wanting to participate or be able to give consent, having uncorrected impairments in vision and/or hearing or motor skills that may impair engagement with the website | 10 | Mean age 73  Range  74-91 | 5F, 5M | 100% White, British | Dementia | All couples were married, ranging from 24-66 years of marriage |
| Swinnen et al., 2023  [119]  Belgium  Mixed methods | All patients with MNCD living at the long-term care facility de Wingerd in Leuven, Belgium were screened for eligibility. | N | 1) have an official MNCD diagnosis, including vascular dementia, Alzheimer’s disease, mixed dementia, Parkinson’s disease, or Lewy-body dementia, 2) 60 years old or more, 3) has the capacity to provide consent for the study, 4) has the appropriate visual acuity (including with correction) to see the TV screen, and 5) have been living in the facility for at least two weeks | 1) have an unstable health condition that prevents the safe participation in exergames and 2) have a mobility issue that prevents upright exercising | n = 18  7 were in the exergame intervention arm and 11 were in the active control arm (traditional exercise) | Mean: 83  Range: 66-91 | 17 females and 1 male  94.4% female | NR | Dementia | The majority of study participants (11/18) have AD. While patients must be able to ambulate independently, they’re free to use mobility aids. |
| Tak et al., 2013 [120]  USA  Qualitative | Three nursing homes in a Southern state. | N | (a) age 65 years or older; (b) diagnosis of dementia in the medical record; (c) Mini-Mental State Examination (MMSE) score of 4–27; (d) no change in psychoactive medications within the past 30 days; and (f) at least 2 weeks’ residency in the nursing home. | NR | 14 | Mean age 81  Range 61-102 | 8F, 6M | 9 African American  5 White | Dementia | Mean education level of 12th grade. |
| Tak et al., 2015 [121]  USA Mixed Methods | Three nursing homes in an American southern state. | N | (a) ≥ 55 years of age; (b) a diagnosis of dementia documented in their medical record; (c) Mini-Mental State Examination (MMSE) score of 10–25; (d) the ability to express preferences; (e) the ability to click a trackball or touch screen; (f) the ability to read an 18-point letter on a computer screen; (g) the ability to hear a sound at the highest level of volume from a computer speaker; (h) at least 2 months’ residence in the nursing home; and (i) exhibition of agitated behaviors in the past week as reported by staff or in documentation | Participation in a daily formal recreation therapy or taking a new medication (i.e., cholinesterase inhibitors, antidepressants, anxiolytics, and pain medications) for less than 1 month. | 27 | Mean age 85  Range 73-97 | 78% F  22% M | 96% Caucasian | Dementia | On average participants took 1.6 anticholinergic medications |
| Tamura et al., 2004 [122]  Japan  Qualitative | NR | NA | Severe dementia staying in a geriatric health care facility | NR | 13 | Mean age 84 | 12F, 1M | NR | Dementia, | NR |
| Taylor et al., 2021  [123]  Australia  Mixed methods | Aged-Care | NA | No mentioned other than that the *sessions* were held in an aged-care facility in Sydney, Australia | NR | n = 20 | Mean age NR  Range: 65-96 | 5 males and 15 females (75% female) | NR | Dementia | This study was poorly written as it did not mention participant characteristics. |
| Topo et al., 2004 [124]  Finland, Ireland, Norway, & UK  Mixed Methods | One dementia care unit in the UK, Norway, and Ireland, and two units in Finland. | Day care units were selected for the assessment environment for several reasons. First, one stipulation for the PG assessment was access to a PC with a touch screen and a CD-ROM drive. Such PCs are relatively expensive, and more people can use the same computer in a day care centre. Second, day care units support the continuity of home care and, because the main aim of the ENABLE project is to find solutions which could support living at home, they were found to fit in well with the aim of the project. Third, the idea of the Editor program is that it will provide a new tool for dementia care, so day care unit staff were expected to be sufficiently motivated to participate in the study. | Sight and hearing were good enough to hear the music and read the lyrics on the screen and that music had played some kind of positive role in their life | NR | 23 | Mean age 77.5  Range 60-89 | 65% F, 35% M | 17% English, 52% Finnish, 17% Irish, 13% Norwegian | Dementia, Alzheimer’s, Vascular dementia | One participant had great difficulties in talking so that people could understand him, and one could only make himself understood through gestures. In addition, four had at least moderate problems with sleeping and three had marked or severe symptoms of physical discomfort. |
| Travers & Bartlett, 2010 [125]  Australia  Mixed Methods | Community dwelling for older people and residents of RAC facilities. Flyers advertising Silver Memories were widely distributed through community groups (e.g., home and community care services) and social organizations, and advertisements inviting people to participate were placed in local community newsletters. Individual RAC facilities, respite services, and community organizations were also approached directly to invite participation from their residents/members. | N | Age 60 or older, were willing to listen to Silver Memories for at least an hour a day for 3 months, maintain a very simple daily listening diary, and complete baseline and follow-up measures of quality of life, depression, and loneliness. | NR | 114 | Mean age 79.9 | 71.1% F  28.9% M | NR | Dementia | NR |
| Tyack et al., 2017 [24]  UK  Mixed Methods | Participants were recruited from Dementia Cafés with their caregivers in inner city London and rural locations in southeast England | N | Older adult with dementia | NR | 12 older adults  12 caregivers | Mean age 75  Range 64-90 | NR | 100% White | Dementia | NR |
| Ulbrecht et al., 2012 [126]  Germany  Mixed Methods | Due to different organizational and staffing structures in the three nursing homes, we screened different populations in each: in the first home (Home I), we screened all residents; in the second home (Home II), we screened only those residents who were currently participating in occupational therapy; and in the third home (Home III), we screened only those residents who were currently participating in occupational therapy and who were living in a specific residential unit. | N | Residents currently participating in occupational therapy | Bedridden, blind, bilateral paralysis of arms or hands | 79 | Mean age 79.3 | 67.1% F  32.9% M | NR | Dementia | 27% had a medical diagnosis of dementia |
| Unbehaun et al., 2018 [127]  Germany  Qualitative | Our study draws upon research in both day-care facilities and domestic environments. It was conducted over a period of about 8-months and used a co-design approach involving people with dementia, their caregivers, and researchers from a number of domains. | We adopted this approach to get meaningful insights regarding the participants’ and other stakeholders’ daily routines, their biographical background, their memories and social environment, their experience of using technology, and their attitudes and practices. | People with dementia | (1) their dementia was beyond the moderate stage; (2) they were suffering from chronic diseases such as cardiovascular illness or cancer; and (3) their physical fitness was impaired such that they could not walk without assistance | 14 | Range  72-89 | 6F, 8M | NR | Dementia | NR |
| van Santen et al., 2020 [111]  The Netherlands  Quantitative | Recruited in day centres; The investigator  instructed DCC staff in a one-hour meeting about the research  study procedures and, for the exergaming group, the intervention (this could also be done by the provider of the equipment).  Dyads (participant with dementia, informal caregiver) for the study were recruited by DCC staff. | NA | All ages, community-dwelling, a diagnosis of any type of dementia, visits the DCC at least 2 days per week, has an IC willing to participate, and are not expected to be admitted into residential care in the coming 6 months. | Exclusion criteria were as follows: severe physical disorders or (terminal) diseases (other than dementia) that would make participation in an exergaming activity impossible (according to DCC staff). | 112 dyads | Mean age 79 | Exergame group:  36F, 37M  Control group:  16F, 23M | NR | Dementia |  |
| Weybright et al., 2010 [128]  USA  Quantitative | Older adults with MCI from a local community assisted living facility were recruited with help from the facility recreation therapist. | N | (a) expressed an interest in bowling and playing an interactive video game, and (b) had sufficient seated balance, ambulation | None | 2 | 86 & 93 | 100% F | NR | MCI | NR |
| Yu et al., 2019 [129]  USA  Mixed Methods | Participants were recruited using a variety of strategies, including advertisements; study flyer posting and distribution to senior facilities and community centers; referral from community partners; and presentations at community and professional events. Respondents to recruitment were screened over the phone (~5 minutes) and in person (~30 minutes) by a trained graduate research assistant (RA). | N | English speaking; diagnosis of dementia confirmed by healthcare providers; stable on dementia medication(s) for at least 1 month (no dosing changes in the past month); having an identified caregiver; and signed informed consent or surrogate consent/assent | None | 80 | Mean age 82.1  74-90 | 58% F  42% M | NR | Dementia | NR |
| Zamir et al., 2020 [130]  UK  Qualitative | Recruitment of older people and relevant family from three care homes that were part of a larger CAR study for video call use. This was facilitated by staff in the care environment | N | All residents had opportunity to take part | None | 22 | 65+ | 5M, 17F | NR | Dementia, MCI | 12 hearing impaired  9 visually impaired  9 nonverbal  6 frail |
| Zamir et al., 2021 [131]  UK  Qualitative | The recruitment and participation of older people was via a convenience sample of four care homes. | N | Managers of the care homes agreed to participate and trial the use of the device. Participants volunteered to participate. | None | 28 | Mean 80  Range  65-97 | 22F, 6M | 100% Caucasian | Dementia, MCI | All participants spoke English as their first language |
| Zhu et al., 2023  [132]  Taiwan  Quantitative quasi-experiment | “Potential participants were recruited from community settings” – not clear regarding sampling and recruitment strategy | N | 1) 65 years old or greater, 2) able to ambulate independently, and 3) has a Clinical Dementia Rating score of 0.5 and Fried’s frailty phenotype (basically the participant must be cognitively frail) | 1) physically unable to perform the activities of the exergames, 2) diagnosed with dementia, 3) diagnosed with vision- or hearing-related diseases, and 4) unable to communicate verbally | n = 69  35 were in the experimental group and 34 in the control group | Mean age: 73 | 17 males and 52 females  % female: 75.4% | NR | Cognitive frailty (cognitive impairment + physical frailty) | 88.4% of the participants had chronic diseases and the average BMI was 22.27 |

*NR = Not Recorded
