# Supplementary material for: Technology-Enabled Recreation and Leisure Programs and Activities for Older Adults With Cognitive Impairment: Rapid Scoping Review
Source: JMIR Neurotechnol. 2024 Aug 8;3:e53038. doi: 10.2196/53038 (PMC12671325; doi:10.2196/53038)
Supplement: Multimedia Appendix 5 [file neuro_v3i1e53038_app5.docx]

| **Table 4: Types of Activities** |  | | | | | | |
| --- | --- | --- | --- | --- | --- | --- | --- |
|  | Art | Music | Video/Audio (non-music) Entertainment | Socializing | Playing Games | Relaxation & Self-Awareness | Exergaming |
| Abdollahi et al., 2017 [44] |  |  | x | x |  | x |  |
| Álvarez, 2022 [45] |  | x |  |  |  | x |  |
| Appel et al., 2020 [46] |  |  |  |  |  | x |  |
| Appel et al., 2021 [47] |  |  |  |  |  | x |  |
| Assche et al., 2021  [48] |  |  |  | x |  |  |  |
| Astell et al., 2016 [49] |  |  |  | x |  |  |  |
| Barrett et al., 2019 [50] |  |  |  | x |  | x |  |
| Benham et al., 2022 [51] |  | x |  | x |  |  |  |
| Berge et al., 2022 [52] |  |  | x | x |  | x |  |
| Chen et al., 2021 [53] |  | x |  | x |  | x |  |
| Cheung et al., 2023 [54] |  |  | x |  |  |  |  |
| Chidester et al., 2016 [55] |  |  |  | x |  |  |  |
| Chu et al., 2017 [56] |  |  |  |  |  |  | x |
| Chu et al., 2021 [57] |  |  |  | x |  |  |  |
| Cruz-Sandoval & Favela, 2019 [58] |  | x |  |  |  |  |  |
| Cunningham et al., 2019 [59] |  | x |  |  |  |  |  |
| Dahms et al., 2021 [60] | x |  |  |  |  |  |  |
| Damianakis et al., 2010 [61] |  |  |  |  |  |  | x |
| D’Cunha et al., 2021 [62] |  |  |  | x |  |  |  |
| Demiris et al., 2016 [63] |  |  |  | x |  | x |  |
| Dinesen et al., 2022 [64] |  |  |  | x |  |  |  |
| D'Onofrio et al., 2019 [65] |  |  |  |  |  |  | x |
| Dove & Astell, 2019 [28] |  |  |  |  |  | x |  |
| Evans et al., 2016 [66] |  |  |  | x |  |  |  |
| Faw et al., 2021 [67] |  |  |  |  |  | x |  |
| Ferguson et al., 2020 [68] |  |  |  | x |  |  |  |
| Fields et al., 2019 [69] |  |  | x |  |  | x |  |
| Givon Schaham et al., 2020[70] |  |  |  |  | x |  |  |
| Groenewoud et al., 2017 [71] |  |  | x  x |  | x  x | x |  |
| Hashim et al., 2015 [72] |  | x |  |  |  |  |  |
| Hebert et al., 2018 [73] |  |  |  | x |  |  |  |
| Hird et al., 2024  [74] |  |  |  | x |  |  |  |
| Hoel et al., 2022 [75] |  |  |  | x |  |  |  |
| Hung et al., 2021 [76] |  | x |  |  |  | x |  |
| Jøranson et al., 2016 [77] |  |  | x |  |  |  | x |
| Kajiyama et al., 2007 [78] |  |  |  | x |  |  |  |
| Kalantari et al., 2022 [79] |  |  |  | x |  |  |  |
| Kelly et al., 2021 [80] |  | x | x |  |  | x |  |
| Khosla et al., 2021 [81] |  |  |  | x |  |  |  |
| Kim et al., 2020 [82] | x |  |  | x |  |  |  |
| Koh & Kang , 2018 [83] | x |  |  | x |  |  |  |
| Kontos et al., 2021 [84] |  |  |  | x |  |  |  |
| Kosurko et al., 2022 [85] |  | x |  |  |  |  |  |
| Kouroupetroglou et al., 2017[86] |  | x |  |  |  |  |  |
| Kuot et al., 2021 [87] |  | x |  |  |  |  |  |
| Lancioni et al., 2015 [88] |  |  | x | x |  | x |  |
| Lancioni et al., 2015 [89] | x |  |  |  |  |  |  |
| Lazar et al., 2016 [90] |  |  |  |  |  |  | x |
| Lazar et al., 2016 [91] | x |  |  |  |  |  |  |
| Leahey & Singleton, 2011 [92] |  |  | x |  |  |  |  |
| Leuty et al., 2013 [93] |  |  |  | x |  |  |  |
| Li et al., 2022 [94] |  | x |  |  |  |  |  |
| Liang et al., 2017 [95] |  |  |  | x |  |  |  |
| Mandzuk et al., 2018 [96] | x |  |  |  |  |  |  |
| Masoud et al., 2021 [97] |  |  |  | x |  |  |  |
| Massimi et al., 2008 [98] |  |  |  |  |  |  | x |
| McCarron et al., 2019 [99] |  |  |  |  |  | x |  |
| Merilampi et al., 2018 [100] |  | x |  |  |  | x |  |
| Moon & Park, 2020 [101] |  |  |  | x |  |  |  |
| Nijhof et al., 2013 [102] |  | x |  |  |  |  |  |
| Obayashi et al., 2020 [103] |  |  |  | x |  | x |  |
| Olsen et al., 2000 [104] |  | x |  |  |  |  |  |
| Park et al., 2023  [105] |  |  |  |  | x |  |  |
| Peeters et al., 2016 [106] |  |  |  | x |  |  |  |
| Perugia et al., 2017 [107] |  |  |  | x |  |  |  |
| Perugia et al., 2017 [108] |  |  |  | x |  |  |  |
| Prophater et al., 2021 [109] |  |  |  |  |  |  | x |
| Šabanovic et al., 2013 [110] |  |  |  | x |  |  |  |
| Santen et al., 2020 [111] |  |  | x | x |  | x | x |
| Samuelsson & Ekström, 2019 [112] |  | x |  |  |  |  |  |
| Scase et al., 2018 [113] |  |  |  |  |  |  | x |
| Sixsmith et al., 2010 (a, b, c) [114] |  |  |  |  |  |  | x |
| Smith et al., 2009 [115] | x |  |  |  |  |  |  |
| Smith & Argentia, 2020 [116] |  |  |  |  | x |  | x |
| Subramaniam & Woods, 2016 [117] |  |  |  |  |  | x |  |
| Sweeney et al., 2021 [118] |  |  |  |  |  | x | x |
| Swinnen et al., 2023  [119] |  | x | x |  | x |  |  |
| Tak et al., 2013 [120] |  |  | x | x |  | x |  |
| Tak et al., 2015 [121] |  | x |  |  | x |  |  |
| Tamura et al., 2004 [122]  Japan  Qualitative |  | x |  |  |  |  |  |
| Taylor et al., 2021  [123] |  | x |  |  |  |  |  |
| Topo et al., 2004 [124]  Finland, Ireland, Norway, & UK  Mixed Methods | x |  |  |  |  |  |  |
| Travers & Bartlett, 2010 [125]  Australia  Mixed Methods |  |  |  |  |  |  | x |
| Tyack et al., 2017 [24] |  |  |  |  |  |  | x |
| Ulbrecht et al., 2012 [126] |  |  |  |  |  |  | x |
| Unbehaun et al., 2018 [127] |  |  |  |  |  |  | x |
| van Santen et al., 2020 [111] |  |  | x | x |  | x |  |
| Weybright et al., 2010 [128] |  |  | x |  |  |  |  |
| Yu et al., 2019 [129] |  |  |  | x |  |  |  |
| Zamir et al., 2020 [130] |  |  |  |  |  |  | x |

Art includes in art display, engaging platform for art development (ePAD), interactive frames, and art viewing apps.

Music includes MP3 players, radio cabinet, listening to music, portable music player, and personalized iTunes library.

Video/Audio (non-music) Entertainment includes town hall meetings, recorded Chaplain sermons, watching YouTube and sports.

Socializing includes social robots (e.g. PARO, Sophie and Jack, MARIO, NAO, Eva, Cota, Betty), virtual pet companion, social recognition watch, conversational activities, Skype on Wheels (SoW), and Memory Cafes.

Playing Games includes iPad games, toy dog, and Pleo (robot dinosaur).

Relaxation & Self-Awareness includes VR beach scene/nature, personal cards with digitally recorded messages, digital life storybook, and old photographs on a tablet.

Exergaming includes Wii bowling, interactive cycling, MS Kinect, bicycle pedaling unit, exergaming mat, and physical activity app.
