# Supplementary material for: Technology-Enabled Recreation and Leisure Programs and Activities for Older Adults With Cognitive Impairment: Rapid Scoping Review
Source: JMIR Neurotechnol. 2024 Aug 8;3:e53038. doi: 10.2196/53038 (PMC12671325; doi:10.2196/53038)
Supplement: Multimedia Appendix 6 [file neuro_v3i1e53038_app6.docx]

**Table 5: Creation of Technology**

| **Article Title** | **Tech-Delivery** | **Created (In house, OTS off-the-shelf [OTS], both)** |
| --- | --- | --- |
| A pilot study on using an intelligent life-like robot as a companion for elderly individuals with dementia and depression | Robot | Both |
| Neurologic Music Therapy with a Habilitative  Approach for Older Adults with Dementia: A Feasibility Study | Television | Both |
| Older Adults With Cognitive and/or Physical Impairments Can Benefit From Immersive Virtual Reality Experiences: A Feasibility Study | Virtual Reality | OTSOTS |
| Administering Virtual Reality Therapy to Manage Behavioral and Psychological Symptoms in Patients With Dementia Admitted to an Acute Care Hospital: Results of a Pilot Study | Virtual Reality | OTSOTS |
| The role of a socially assistive robot in enabling  older adults with mild cognitive impairment  to cope with the measures of the COVID-19  lockdown: A qualitative study | Robot | OTS |
| Does familiarity affect the enjoyment of touchscreen games for people with dementia? | Tablet | OTSOTS |
| Evaluation of a Companion Robot for Individuals With Dementia: Quantitative Findings of the MARIO Project in an Irish Residential Care Setting | Robot | OTSOTS |
| Effects of Community-Based Virtual Reality on  Daily Activities and Quality of Life | Virtual Reality | Both |
| The acceptability, adoption, and feasibility of a music application developed using participatory design for home-dwelling persons with dementia and their caregivers. The “Alight” app in the [LIVE@Home.Path](mailto:LIVE@Home.Path) trial | Tablet | Both |
| Exploring the acceptance of tablets usage for cognitive training among older people with cognitive impairments: A mixed-methods study | Tablet | OTSOTS |
| A Home-Based Dyadic Music-with-Movement  Intervention for People with Dementia and  Caregivers: A Hybrid Type 2 Cluster-Randomized  Effectiveness-Implementation Design | Not specified | In house |
| Transforming dementia care and quality of life using innovative touch screen computer engagement: a research study-the birdsong initiative | Desktop Computer | Both |
| Exergaming platform for older adults residing in long-term care homes: User-centered design, development and usability study | Game Console | Developed In-House |
| Service innovation through social robot engagement to improve dementia care quality | Robot | Both |
| Incorporating Conversational Strategies in a Social Robot to Interact with People with Dementia | Robot | Developed In-House |
| Assessing Wellbeing in People Living with Dementia Using Reminiscence Music with a Mobile App (Memory Tracks): A Mixed Methods Cohort Study | Mobile Phone | Developed In-House |
| The psychosocial impacts of multimedia biographies on persons with cognitive impairments | Television | Developed In-House |
| Influence of Music Therapy and Music-Based Interventions on Dementia: A Pilot Study | Mobile devices | Both |
| Effects of a virtual group cycling experience on people  living with dementia: A mixed method pilot study | Video - GoPro Hero 7 | OTSOTS |
| Evaluation of a Digital Companion for Older Adults with Mild Cognitive Impairment | Tablet | Developed In-House |
| Use of a Social Robot (LOVOT) for Persons With Dementia: Exploratory Study | Robot | In-house |
| MARIO Project: Validation and Evidence of Service Robots for Older People with Dementia | Robot | Both |
| The Kinect Project: Group motion-based gaming for people living with dementia | Game Console | OTSOTS |
| Personal message cards: An evaluation of an alternative method of delivering simulated presence therapy | MP3 | Developed In-House |
| Being There: Exploring Virtual Symphonic Experience as a Salutogenic Design Intervention for Older Adults | VR | Developed In-house |
| Virtual Reality for Therapeutic Recreation in Dementia Hospice Care: A Feasibility Study | Virtual Reality | OTSOTS |
| Shall I compare thee...to a robot? An exploratory pilot study using participatory arts and social robotics to improve psychological well-being in later life | Robot | Both |
| Electrodermal activity: Explorations in the psychophysiology of engagement with social robots in dementia | Robot | OTSOTS |
| The Development and Feasibility of TECH: Tablet Enhancement of Cognition and Health, a Novel Cognitive Intervention for People with Mild Cognitive Impairment | Tablet | Both |
| People with dementia playing casual games on a tablet | Tablet | Both |
| Sustaining Our Relationship: Dyadic Interactions Supported by Technology for People with Dementia and Their Informal Caregivers | Tablet | Both |
| The development of an individualized digital memory book for Alzheimer's Disease patient: A case study | Mobile Phone | Developed In-House |
| Implementation of Individualized Music in Long-Term Care: Application of the PARiHS Framework | MP3 | OTS |
| Enabling Personalization for Digital Cognitive Stimulation to Support Communication With People With Dementia: Pilot Intervention Study as a Prelude to AI Development | Tablet | Developed in-house |
| Exploring the perceptions of people with dementia about the social robot PARO in a hospital setting | Robot | OTS |
| Exploring the perceptions of people with dementia about the social robot PARO in a hospital setting | Robot | OTS |
| Using a Nature-Based Virtual Reality Environment for  Improving Mood States and Cognitive Engagement in  Older Adults: A Mixed-Method Feasibility Study | VR | Both |
| Effects of Intervention Using PARO on the Cognition, Emotion, Problem Behavior, and Social Interaction of Elderly People with Dementia | Robot | OTS |
| Group activity with Paro in nursing homes: systematic investigation of behaviors in participants | Robot | OTS |
| Improving the quality of life for caregivers and care recipients with personalized video channels | Television | OTS |
| Nature-based video and classical music’s effects on  tranquility among memory care residents with dementia | Television | Both |
| The effect of PARO robotic seals for hospitalized patients with dementia: A feasibility study | Robot | OTS |
| Engagement and experience of older people with socially assistive robots in home care | Robot | Developed In-House |
| Dancing With Dementia: Exploring the Embodied  Dimensions of Creativity and Social Engagement | Remotely streamed videos | Both |
| Interacting with Dementia: The MARIO Approach | Robot | Both |
| Personalised music for residents with dementia in an Australian rural aged‐care setting | MP3 | OTS |
| Effects of response-related music stimulation versus general music stimulation on positive participation of patients with Alzheimer's disease | Desktop Computer | Developed In-House |
| Persons with Alzheimer's disease engage in leisure and mild physical activity with the support of technology-aided programs | Not mentioned | In house |
| Evaluation of a multifunctional technology system in a memory care unit: Opportunities for innovation in dementia care | Desktop Computer | OTS |
| Supporting People with Dementia in Digital Social Sharing | Tablet | Developed In-House |
| Utilizing Therapeutic Recreation to Empower Persons with Alzheimer's in a Day Center | Game Console | OTS |
| Engaging older adults with dementia in creative occupations using artificially intelligent assistive technology | Tablet | Developed In-House |
| A Pilot Randomized Trial of a Companion Robot for People With Dementia Living in the Community | Robot | OTS |
| A cognitively enhanced online Tai Ji Quan  training intervention for community-dwelling  older adults with mild cognitive impairment:  A feasibility trial | Not specified | In-house |
| ﻿Personalized music... so simple yet so powerful: A pilot initiative for hospitalized older adults living with dementia in an acute care facility | MP3 | OTS |
| We Don't Feel so Alone: A Qualitative Study of Virtual Memory Cafes to Support Social Connectedness Among Individuals Living With Dementia and Care Partners During COVID-19 | Laptop | Both |
| An exploratory case study of the impact of ambient biographical displays on identity in a patient with Alzheimer's disease | Tablet | Both |
| A Web-Based Mobile App With a Smartwatch to Support Social Engagement in Persons With Memory Loss: Pilot Randomized Controlled Trial | Mobile Phone | OTS |
| Activation game for older adults Development and initial user experiences | Game Console | Developed In-House |
| The effect of digital reminiscence therapy on people with dementia: a pilot randomized controlled trial | Tablet | Developed In-House |
| The behavioral outcomes of a technology-supported leisure activity in people with dementia | Television | OTS |
| Measuring the impact of age, gender and dementia on communication-robot interventions in residential care homes | Robot | Developed In-House |
| ‘Media Memory Lane' interventions in an Alzheimer's day care center | Multi, MP3, Video | Developed In-House |
| ‘Now I can bend and meet people virtually in my home’: The experience of a remotely supervised online chair yoga intervention and visual socialisation among older adults with dementia | Zoom via Tablet or computer | OTS |
| Feasibility and benefits of computerized cognitive exercise to adults with chronic moderate-to-severe cognitive impairments following an acquired brain injury: A pilot study | Desktop Computer | OTS |
| Designing a personal music assistant that enhances the social, cognitive, and affective experiences of people with dementia | Tablet | OTS |
| Alzheimer’s Association Project VITAL: A Florida Statewide Initiative Using Technology to Impact Social Isolation and Well-being | Tablet | OTS |
| Modelling engagement in dementia through behaviour. Contribution for socially interactive robotics | Social Robot | OTS |
| PARO robot affects diverse interaction modalities in group sensory therapy for older adults with dementia | Robot | OTS |
| Digital communication support in interaction involving people with dementia | Tablet | OTS |
| Development and Evaluation of Cognitive Games to Promote Health and Wellbeing in Elderly People with Mild Cognitive Impairment | Tablet | Both |
| Developing a music player for people with dementia | Music Player | Developed In-house |
| Multimedia biographies: a reminiscence and social stimulus tool for persons with cognitive impairment | Multi, MP3, Video | OTS |
| The Usability of Physical Activity and Cognitive Training Applications in People with Mild Cognitive Impairment | Tablet | OTS |
| Cost-effectiveness of exergaming compared to regular  day-care activities in dementia: Results of a randomised  controlled trial in The Netherlands | Not specified | Developed in-house |
| Digital life storybooks for people with dementia living in care homes: an evaluation | Television | OTS |
| Understanding the shared experiences of creating a digital life story with individuals with dementia and their spouse | Tablet | OTS |
| The feasibility of a stepping exergame prototype for older adults with major neurocognitive disorder residing in a long-term care facility: a mixed methods pilot study | Laptop projection to TV | OTS |
| Feasibility of providing computer activities for nursing home residents with dementia | Desktop Computer | OTS |
| Computer Activities for Persons with Dementia | Desktop Computer | OTS |
| Is an entertainment robot useful in the care of elderly people with severe dementia? | Robot | OTS |
| New musical interfaces for older adults in residential care: assessing a user-centred design approach | Tablet or physical DMI | In-house |
| Assessment of a music-based multimedia program for people with dementia | Desktop Computer | Both |
| An exploratory study of carers' and care staff's perspectives of Silver Memories---a unique radio program for older people | MP3 | OTS |
| Viewing Art on a Tablet Computer: A Well-Being Intervention for People with Dementia and Their Caregivers | Tablet | OTS |
| Exergames and Their Acceptance Among Nursing Home Residents | Game Console | OTS |
| Exploring the Potential of Exergames to affect the Social and Daily Life of People with Dementia and their Caregivers | Television | Both |
| Effects of Exergaming on Cognitive and Social Functioning of People with Dementia: A Randomized Controlled Trial | Television | OTS |
| Effects of an Interactive Video Game (Nintendo Wii™) on Older Women with Mild Cognitive Impairment | Game Console | OTS |
| Memory matters in dementia: Efficacy of a mobile reminiscing therapy app | Telephone | Both |
| Intergroup 'Skype' Quiz Sessions in Care Homes to Reduce Loneliness and Social Isolation in Older People | Tablet | OTS |
| Aesthetically Designing Video-Call Technology with Care Home Residents: A Focus Group Study | Tablet | OTS |
| Dance Wherever You Are: The Evolution of Multimodal Delivery for Social Inclusion of Rural Older Adults | Tablet and TV | OTS |
| Effects of exergaming on cognitive functions and loneliness of older adults with cognitive frailty | Laptop projection to TV | OTS |
