# Supplementary material for: Technology-Enabled Recreation and Leisure Programs and Activities for Older Adults With Cognitive Impairment: Rapid Scoping Review
Source: JMIR Neurotechnol. 2024 Aug 8;3:e53038. doi: 10.2196/53038 (PMC12671325; doi:10.2196/53038)
Supplement: Multimedia Appendix 7 [file neuro_v3i1e53038_app7.docx]

**Table 6: Study Outcomes and Measurements**

| **First Author, Year, Country & Design** | **Outcome of Interest** | **Measurements** |
| --- | --- | --- |
| Abdollahi et al., 2017 [44]  USA  Qualitative | Neutral | Data collection via robot (Ryan). Survey |
| Álvarez, 2022 [45]  USA  Prospective, single-anonymized, crossover-  group design | Positive | Treatment acceptability was measured by the group facilitators tracking attendance: acceptance of treatment and length of stay in session. Behavior, mood, and cognition were measured at the same time for all cohorts through three research assistant-administered instruments. |
| Appel et al., 2020 [46]  Canada  Mixed Methods | Positive | Time spent in VR noted. Using detailed guide to observe participant during VR experience. |
| Appel et al., 2021 [47]  Canada  Mixed Methods | Positive | Time spent in VR noted. Using detailed guide to observe participant during VR experience. |
| Assche et al., 2021  [48]  Belgium  Qualitative | Positive | Pre-intervention questionnaire and post-intervention structured interviews |
| Astell et al., 2016 [49]  UK  Mixed Methods | Positive | Two Sony HD Handycam digital video recorders with tripods were used to record all data collection sessions. |
| Barrett et al., 2019 [50]  Ireland  Mixed Methods | Mixed | Observations and questionnaires |
| Benham et al., 2022 [51]  Country not specified  Exploratory study | Mixed | Virtual Reality sessions using Oculus Go |
| Berge et al., 2022 [52]  Country not specified  Mixed methods | Positive | Control group |
| Chen et al., 2021 [53]  China  Mixed Methods | Positive | A convergent parallel mixed-methods design, comprising a post-usage questionnaire survey and focus groups |
| Cheung et al., 2023 [54]  China  Clustered randomized controlled trial | Positive | Readiness assessment, audit and providing feedback |
| Chidester et al., 2016 [55]  USA  Mixed Methods | Mixed | Blood pressure readings from medical records. Saliva tests. Observation and documentation by staff. Scoring on Montreal Cognitive Assessment (MoCA). Affect Balance Scale. Geriatric Depression Scale. |
| Chu et al., 2017 [56]  Australia  Mixed Methods | Positive | Data collection via robot (Sophie and Jack) |
| Chu et al., 2021 [57]  Canada  Qualitative | Positive | User-Centered Design (UCD) process that consisted of 4 rounds of usability testing. Semi-structured interviews. Standardized and validated scales. |
| Cruz-Sandoval & Favela, 2019 [58]  Mexico  Quantitative | Positive | Observations from conversations |
| Cunningham et al., 2019 [59]  UK Mixed Methods Cohort Study | Positive | Interviews with care staff only. Surveys. Observations. |
| Dahms et al., 2021 [60]  Germany  Pilot Study | Mixed | Questionnaires |
| Damianakis et al., 2010 [61]  Country not specified  Qualitative | Mixed | Observational and in-depth interviews. Strategies typically used by computer scientists |
| D’Cunha et al., 2021 [62]  Australia  Mixed methods | Mixed | Pre and post condition questions, interviews, video data evaluation |
| Demiris et al., 2016 [63]  USA  Mixed Methods | Positive | Phone screening form that also included the administration of the Memory Impairment Screen-Telephone instrument |
| Dinesen et al., 2022 [64]  Denmark  Exploratory Study | Positive | Observation |
| D'Onofrio et al., 2019 [65]  Ireland, Italy, & UK  Mixed Methods | Mixed | Observation and use of a scale |
| Dove & Astell, 2019 [28]  Canada  Qualitative | Mixed | Descriptive field notes |
| Evans et al., 2016 [66]  UK  Qualitative | Positive | Reviews |
| Faw et al., 2021 [67]  UK | Mixed | Focus Groups |
| Ferguson et al., 2020 [68]  USA  Quantitative | Positive | Documentation of total "wear time" recorded in minutes. Pain Assessment IN Advanced Dementia Scale. |
| Fields et al., 2019 [69]  USA  Quantitative | Positive | Quantitative surveys: FaceScale, UCLA loneliness scale, Geriatric Depression scale |
| Givon Schaham et al., 2020[70]  Finland  Mixed Methods | Positive | "Screen Time" app. Questionnaire. |
| Groenewoud et al., 2017 [71]  The Netherlands  Mixed Methods | Mixed | Observations. Interviews at end of each session. |
| Hashim et al., 2015 [72]  Malaysia  Quantitative | Positive | Evaluation form (questionnaire) |
| Hebert et al., 2018 [73]  USA  Mixed Methods | Positive | Qualitative descriptive evaluation of resident and staff stakeholders' perspectives at initiation and completion of the implementation period. Quantitative data collected at end of the implementation period. |
| Hird et al., 2024  [74]  Japan  Mixed methods pilot study | Positive | Personal profile creation based on interview with family members. Video and audio recording from patient’s sessions and user inputs and engagement. MENFIS, Engagement of a Person with Dementia Scale (EPWDS), and Neuropsychiatric Inventory – Nursing Home edition (NPI-NH). Social engagement and communication as observed by care home staff. |
| Hoel et al., 2022 [75]  Country not specified  Mixed methods | Positive | A paired t-test was used to assess the differences in scores between baseline and post-intervention |
| Hung et al., 2021 [76]  Canada  Qualitative | Positive | Video ethnography |
| Jøranson et al., 2016 [77]  Norway | Positive | Ethnogram. |
| Kajiyama et al., 2007 [78]  Country not specified  Qualitative | Positive | Observed Emotion Rating Scale. |
| Kalantari et al., 2022 [79]  USA  Mixed Method Feasibility study | Positive | The researchers developed a novel VR environment that combined 360-degree videos of natural areas and botanical gardens with interactive digital features that allowed users to engage with aspects of the environment. |
| Kelly et al., 2021 [80]  USA  Mixed Methods | Positive | Single group pre-post PARO intervention physiological measurements |
| Khosla et al., 2021 [81]  Australia  Mixed Methods | Positive | Observation. Survey questionnaire. |
| Kim et al., 2020 [82]  USA  Case Study | Positive | Observation |
| Koh & Kang , 2018 [83]  Korea  Quantitative | Mixed | Questionnaire. Direct observation. Video recording. |
| Kontos et al., 2021 [84]  Canada  Qualitative | Positive | The larger study involved qualitative data collection (i.e.,  participant observation, video recordings, diaries, focus  groups, interviews, and reflections) |
| Kosurko et al., 2022 [85]  Canada  Qualitative | Positive | Observations, research team reflections, focus groups, and interviews |
| Kouroupetroglou et al., 2017[86]  Ireland & Italy  Mixed Methods | Mixed | Two questionnaires (one for observational data from researcher, one for the participant) |
| Kuot et al., 2021 [87]  Australia  Qualitative | Positive | Transcript analysis. |
| Lancioni et al., 2015 [88]  Country not specified  Mixed Methods | Mixed | Observation. |
| Lancioni et al., 2015 [89]  Country not specified  Observation | Positive | Observation. |
| Lazar et al., 2016 [90]  USA  Mixed Methods | Mixed | Mini-Mental State Examination (MMSE), Quality of Life in Alzheimer's Disease (QOL-AD), Cornell Scale for Depression and Dementia (CSDD), Resource Utilization Scale |
| Lazar et al., 2016 [91]  Country not specified  Qualitative | Mixed | Observations of interactions between people living with dementia and art therapists. Interviews with family members and friends of people living with dementia, and art therapists. |
| Leahey & Singleton, 2011 [92]  Canada  Qualitative | Positive | Observation. Field notes. |
| Leuty et al., 2013 [93]  Canada  Mixed Methods | Mixed | Written comments. Questionnaires that used five-point Likert rating scales. |
| Li et al., 2022 [94]  USA  Feasibility trial | Positive | Randomized blinding |
| Liang et al., 2017 [95]  New Zealand  Mixed Methods | Positive | Observation. |
| Mandzuk et al., 2018 [96]  Canada  Qualitative | Positive | Observation. |
| Masoud et al., 2021 [97]  USA  Qualitative | Positive | Interviews. |
| Massimi et al., 2008 [98]  Country not specified  Mixed Methods | Mixed | Standardized tests. Custom interview. Custom questionnaire. |
| McCarron et al., 2019 [99]  USA  Mixed Methods | Negative | Qualitative interviews following completion of 6-month survey. |
| Merilampi et al., 2018 [100]  China  Qualitative | Positive | Observation and note taking of play and players' comments |
| Moon & Park, 2020 [101]  Korea  Quantitative | Positive | Digital RT group compared to control group (conventional storytelling) |
| Nijhof et al., 2013 [102]  The Netherlands  Mixed Methods | Positive | Observations during sessions of both activities using Oshkosh Social Behaviour Coding (OSBC) scale. Semi-structured interviews with activity facilitators |
| Obayashi et al., 2020 [103]  Japan  Quantitative | Positive | WHO International Classification of Functioning, Disability, and Health. |
| Olsen et al., 2000 [104]  USA  Qualitative | Positive | Observed activity therapists. |
| Park et al., 2023  [105]  USA  Qualitative | Positive | Focus group interviews both pre- and post-intervention with stakeholders (dementia patients and their caretakers) |
| Peeters et al., 2016 [106]  The Netherlands  Mixed Methods | Positive | Observation. |
| Perugia et al., 2017 [107]  Spain  Mixed Methods | Mixed | Observation. |
| Perugia et al., 2017 [108]  Spain  Quantitative | Mixed | Observation. |
| Prophater et al., 2021 [109]  United States  Qualitative | Positive | Observation from the tablet usage |
| Šabanovic et al., 2013 [110]  USA  Mixed Methods | Positive | Observation. |
| Santen et al., 2020 [111]  Netherlands  Randomized controlled trial | Mixed | Observation. Regular activity (music, signing, arts, and crafts) program and control group |
| Samuelsson & Ekström, 2019 [112]  Sweden  Qualitative | Positive | Twenty-one video recorded activities |
| Scase et al., 2018 [113]  Italy & UK  Qualitative | Positive | Semi-structured interviews. Individual interviews. |
| Sixsmith et al., 2010 (a, b, c) [114]  UK  Qualitative | Positive | Interviews. Observations. Biographical data. Floor plans for each of the two homes. Observational and semi-structured interviews. |
| Smith et al., 2009 [115]  Country not specified  Qualitative | Positive | Video recording. Semi-structured interviews. |
| Smith & Argentia, 2020 [116]  USA  Qualitative | Mixed | Observational data of computer activity sessions. Interviews prior to and after computer activity program. |
| Subramaniam & Woods, 2016 [117]  UK  Mixed Methods | Positive | Qualtiative participatory acion and questionnaires. |
| Sweeney et al., 2021 [118]  UK  Qualitative | Positive | Interviews |
| Swinnen et al., 2023  [119]  Belgium  Mixed methods | Mixed | Qualitative: post-intervention semi-structured interviews.  Quantitative: various tests for cognitive, mental, and physical health and quality of life both pre- and post-intervention. |
| Tak et al., 2013 [120]  USA  Qualitative | Mixed | . Observational data of computer activity sessions. Interviews prior to and after computer activity program.  Field notes. |
| Tak et al., 2015 [121]  USA Mixed Methods | Mixed | . Observational data of computer activity sessions. Interviews prior to and after computer activity program.  Field notes. |
| Tamura et al., 2004 [122]  Japan  Qualitative | Positive | Field notes. |
| Taylor et al., 2021  [123]  Australia  Mixed methods | Positive | Quantitative scales of enjoyment and engagement measured by facilitators and staff. Qualitative data collected via post-intervention interviews with participants. |
| Topo et al., 2004 [124]  Finland, Ireland, Norway, & UK  Mixed Methods | Mixed | MM evaluation study. Interviews. Surveys. Observations. |
| Travers & Bartlett, 2010 [125]  Australia  Mixed Methods | Positive | Survey. Interviews. |
| Tyack et al., 2017 [24]  UK  Mixed Methods | Positive | Pen-and-paper versions of the Quality of Life-Alzheimer's Disease (QoL-AD). Semi-structured interviews. |
| Ulbrecht et al., 2012 [126]  Germany  Mixed Methods | Mixed | Scales. Questionnaire. |
| Unbehaun et al., 2018 [127]  Germany  Qualitative | Mixed | Semi-structured interviews. Observation. Field notes. |
| van Santen et al., 2020 [111]  The Netherlands  Quantitative | Positive | physical activity, mobility  of the participants with dementia (Short Physical Performances Battery, SPPB), and  Quality-Adjusted Life-Years (QALYs) |
| Weybright et al., 2010 [128]  USA  Quantitative | Positive | Video recording. Staff surveys. Participant interviews. |
| Yu et al., 2019 [129]  USA  Mixed Methods | Positive | Data collectors blinded to group allocations. semi-structured one-on-one exit interviews with participants. Intention-to-treat analysis. Analysis of covariance. |
| Zamir et al., 2020 [130]  UK  Qualitative | Mixed | Observations. Feedback forms. Recorded information (who was called, length of call, number of residents engaged with and any technical problems, if residents understood video calls, enjoyed their use, if they used the telephone handset and if they would like to continue using video  calls). Field notes. Informal unstructured feedback. Memo writing. Semi-structured interviews |
| Zamir et al., 2021 [131]  UK  Qualitative | Positive | Skype Quiz. |
| Zhu et al., 2023  [132]  Taiwan  Quantitative quasi-experiment | Mixed | Cognitive function was measured through the Montreal Cognitive Assessment (MoCA) and loneliness was measured with the Chinese adaptation/translation of the Loneliness Scale. |
